# Glycoproteomic Analysis of the Aortic Extracellular Matrix in Marfan Patients

**DOI:** 10.1161/ATVBAHA.118.312175

**Published:** 2019-06-13

**Authors:** Xiaoke Yin (殷晓科), Shaynah Wanga, Adam L. Fellows, Javier Barallobre-Barreiro, Ruifang Lu, Hongorzul Davaapil, Romy Franken, Marika Fava, Ferheen Baig, Philipp Skroblin, Qiuru Xing, David R. Koolbergen, Maarten Groenink, Aeilko H. Zwinderman, Ron Balm, Carlie J.M. de Vries, Barbara J.M. Mulder, Rosa Viner, Marjan Jahangiri, Dieter P. Reinhardt, Sanjay Sinha, Vivian de Waard, Manuel Mayr

**Affiliations:** 1 From the King’s British Heart Foundation Centre, King’s College London, United Kingdom (X.Y., A.L.F., J.B.-B., R.L., M.F., F.B., P.S., Q.X., M.M.); 2 Department of Medical Biochemistry, Amsterdam Cardiovascular Sciences (S.W., C.J.M.d.V., V.d.W.), Amsterdam UMC, University of Amsterdam, the Netherlands; 3 Department of Cardiology (S.W., R.F., M.G., B.J.M.M.), Amsterdam UMC, University of Amsterdam, the Netherlands; 4 Department of Cardiothoracic Surgery (D.R.K.), Amsterdam UMC, University of Amsterdam, the Netherlands; 5 Department of Radiology (M.G.), Amsterdam UMC, University of Amsterdam, the Netherlands; 6 Department of Clinical Epidemiology, Biostatistics and Bioinformatics (A.H.Z.), Amsterdam UMC, University of Amsterdam, the Netherlands; 7 Department of Surgery (R.B.), Amsterdam UMC, University of Amsterdam, the Netherlands; 8 Department of Medicine, Wellcome-MRC Cambridge Stem Cell Institute, University of Cambridge, United Kingdom (H.D., S.S.); 9 Netherlands Heart Institute, Utrecht (B.J.M.M.); 10 Thermo Fisher Scientific, San Jose, CA (R.V.); 11 St George’s, University of London, United Kingdom (M.J.); 12 Faculty of Medicine, Department of Anatomy and Cell Biology and Faculty of Dentistry, McGill University, Montreal, Canada (D.P.R.).

**Keywords:** elastin, extracellular matrix, glycoproteins, Marfan syndrome, proteomics

## Abstract

Marfan syndrome (MFS) is caused by mutations in FBN1 (fibrillin-1), an extracellular matrix (ECM) component, which is modified post-translationally by glycosylation. This study aimed to characterize the glycoproteome of the aortic ECM from patients with MFS and relate it to aortopathy.

**Approach and Results:**

ECM extracts of aneurysmal ascending aortic tissue from patients with and without MFS were enriched for glycopeptides. Direct N-glycopeptide analysis by mass spectrometry identified 141 glycoforms from 47 glycosites within 35 glycoproteins in the human aortic ECM. Notably, MFAP4 (microfibril-associated glycoprotein 4) showed increased and more diverse N-glycosylation in patients with MFS compared with control patients. MFAP4 mRNA levels were markedly higher in MFS aortic tissue. MFAP4 protein levels were also increased at the predilection (convexity) site for ascending aorta aneurysm in bicuspid aortic valve patients, preceding aortic dilatation. In human aortic smooth muscle cells, MFAP4 mRNA expression was induced by TGF (transforming growth factor)-β1 whereas siRNA knockdown of MFAP4 decreased FBN1 but increased elastin expression. These ECM changes were accompanied by differential gene expression and protein abundance of proteases from ADAMTS (a disintegrin and metalloproteinase with thrombospondin motifs) family and their proteoglycan substrates, respectively. Finally, high plasma MFAP4 concentrations in patients with MFS were associated with a lower thoracic descending aorta distensibility and greater incidence of type B aortic dissection during 68 months follow-up.

**Conclusions:**

Our glycoproteomics analysis revealed that MFAP4 glycosylation is enhanced, as well as its expression during the advanced, aneurysmal stages of MFS compared with control aneurysms from patients without MFS.

## Abstract 2

Supplemental Digital Content is available in the text.

**Highlights**

- Glycoproteomic analysis revealed enhanced MFAP4 (microfibril-associated glycoprotein 4) levels in the extracellular matrix of the ascending aorta from patients with Marfan syndrome compared to aneurysm patients without Marfan syndrome.
- Silencing MFAP4 expression in aortic smooth muscle cells had a profound impact on the expression of elastin and fibrillin-1, as well as ADAMTS (a disintegrin and metalloproteinase with thrombospondin motifs) proteases.
- High levels of plasma MFAP4 might be associated with reduced distensibility of the descending aorta and predict the probability of a type B aortic dissection.

Marfan syndrome (MFS) is an autosomal genetic disease, with at least 90% of patients exhibiting mutations within the gene encoding for the extracellular matrix (ECM) glycoprotein fibrillin-1 (*FBN1*).^1^ In the aorta, FBN1 is linked to vascular smooth muscle cells (SMCs) by integrins as well as within elastin microfibrils within the ECM.^2,3^ *FBN1* mutations are either haploinsufficient or dominant negative, which seems to influence the MFS phenotype.^1^ In addition to aneurysms, aortic dissections constitute a significant number of life-threatening events in patients with MFS.^4^ While dissections of the ascending aorta (type A) are largely prevented by aggressive prophylactic aortic surgery, the dissections of the thoracic descending aorta (type B) usually present asymptomatically later in life, at an aortic diameter far below the 4.5 cm cutoff value for surgery.^5^

One proposed mechanism involved in MFS aorto-pathy is the function of FBN1 as a regulator of TGF-β (transforming growth factor β) bioavailability, whereby defective FBN1 production leads to heightened TGF-β signaling and eventual aortic dilatation and aneurysms in mice. However, clinical studies showed that losartan had no superior therapeutic effect, when compared with or used as an additive to standard β-blocker administration in undifferentiated MFS cohorts.^6–8^ Thus, a better understanding of the downstream consequences of defective FBN1 on ECM remodeling that leads to the structural failure of the vessel wall in MFS is required. ECM remodeling may involve the canonical function of FBN1 in elastic fiber synthesis and cross-linking^9^ as well as post-translational modifications (PTMs) that regulate the function of ECM proteins.

Given that MFS results in high mortality because of aortopathy, a strong focus has been on elastin and collagen, the 2 predominant ECM components governing the structural integrity of the vascular wall.^3^ MFS aortas display not only abnormal organization and metabolism of collagen and elastin fibers with an abnormal appearance of microfibrils^10^ but also a significant component of ECM degradation,^11,12^ as confirmed using 3-dimensional confocal imaging and atomic force microscopy.^13^

Interestingly, mutations in *FBN1* causing MFS include those affecting PTMs of the protein, for example, the generation of an extra N-glycosylation site on FBN1.^14^ Aberrant glycosylation of proteins alters their folding, solubility, binding, and degradation.^15^ Interestingly, a large proportion of ECM proteins contain glycosylation sites, and the glycosylation profiles of FBN1 and other ECM constituents could, therefore, be relevant to the pathology of MFS. Given the technical challenges associated with the study of glycosylation, this key PTM has long been neglected. Only recent advances in glycoproteomics have made it feasible to directly analyze glycopeptides using mass spectrometry (MS).^16,17^ Over recent years, we have developed extraction methods to enrich for ECM proteins in cardiovascular tissues^18^ that are also amenable for direct analysis of glycosylation by MS.^19^

In the present study, we have applied this MS method to analyze the ECM glycoproteome in thoracic aortic aneurysms from patients with and without MFS. For the first time, we demonstrate aberrant glycosylation alongside elevated levels of the FBN1-interacting MFAP4 (microfibril-associated glycoprotein 4) in MFS and provide evidence for the application of MFAP4 as a potential plasma biomarker to identify patients with MFS at risk for type B aortic dissection.

## Materials and Methods

The data that support the findings of this study are available from the corresponding author upon reasonable request.

### Sample Collection From Patients With MFS

For glycoproteomic analysis, aortic aneurysmal tissue was collected during surgery from 11 patients with MFS and 6 patients without MFS who had undergone aortic root and ascending aorta replacement (Table I in the online-only Data Supplement). Of the 6 patients without MFS, 3 had a known mutation, namely for TGF-β receptor type-1 (*TGFBR1*), elastin (*ELN*), and aortic smooth muscle actin (*ACTA2*). No mutation was found for the other 3 patients based on current mutation screening. A local Research Ethics Committee gave approval for the use of these samples when used anonymously (Ref number W16_037 no. 16.052). For plasma analysis, the same patients with MFS, who were from a subset of the COMPARE trial (Cozaar in Marfan Patients Reduces Aortic Enlargement; NTR1423)^20^ and included in a study on plasma TGF-β1 in aortic disease,^21,22^ were used to measure plasma MFAP4 concentration. Inclusion criteria of the COMPARE trial were the diagnosis of MFS was according to the Ghent criteria^23^ relevant at the time and an age of >18 years. The exclusion criteria were angiotensin-converting enzyme inhibitor usage and previous replacement of more than one part of the aorta. However, in this substudy, all patients were included irrespective of the medication they used, and patients with prior aortic surgery were also allowed.^20^ At the time of inclusion, plasma samples (n=96) from patients with MFS were obtained, and aortic root diameters were measured. Written consent was obtained from all patients included in this study.

### ECM Extraction From Aortic Tissue

Human aortic tissues were diced into small pieces and subjected to our 3-step extraction.^18^ First, the tissues were incubated in a sodium chloride (NaCl, 10X volume: weight, ie, 10 µL per 1 mg) buffer (0.5 mol/L NaCl, 10 mmol/L Tris, pH=7.5, 25 mmol/L EDTA, supplemented with Protease Inhibitor Cocktail from Sigma, P8340), with slow agitation for 4 hours. The NaCl fraction was transferred into a new tube and kept at −80°C for later use. Subsequently, the tissues were decellularized by incubation in a sodium dodecyl sulphate (SDS, 10X volume: weight) buffer (0.08% SDS, 25 mmol/L EDTA, supplemented with Proteinase Inhibitor Cocktail) with slow agitation for 4 hours. The SDS fraction was transferred into a new tube and kept at −80°C for later use. Finally, the samples were incubated in a guanidine hydrochloride (GuHCl, 5X volume: weight) buffer (4 mol/L GuHCl, 50 mmol/L sodium acetate, pH=5.8, 25 mmol/L EDTA supplemented with Protease Inhibitor Cocktail) with vigorous agitation for 48 hours. The GuHCl fraction was recovered and stored at −80°C for later use. Protein concentrations of NaCl and GuHCl fractions were estimated using Nanodrop spectrophotometer (ND-1000, Thermo Fisher Scientific) at 280 nm.

### Glycopeptide Enrichment

One hundred micrograms of protein from the GuHCl fraction per sample were precipitated using 100% ethanol overnight at −20°C. After centrifugation at 14000*g* for 30 minutes at 4°C, the pellets were briefly dried using a SpeedVac (Thermo Fisher Scientific, Savant SPD131DDA) and resuspended in 0.1% SDS, 50 mmol/L Tris-HCl, pH=8.8. After reduction with 10 mmol/L dithiothreitol at 37°C for 1 hour, 240 rpm, the samples were cooled down to room temperature before being alkylated by 50 mmol/L iodoacetamide in the dark for 1 hour. Prechilled (−20°C) acetone (8× volume) was used to precipitate the samples overnight at −20°C. Samples were centrifuged at 14000*g* for 30 minutes at 4°C and the supernatant subsequently discarded. Protein pellets were briefly dried using SpeedVac and resuspended in 100 µL of 0.1 mol/L triethylammonium bicarbonate (pH=8.2) then digested with 2 µg of trypsin (MS Grade, TPCK-treated to eliminate chymotryptic activity, Thermo Fisher Scientific) at 37°C overnight. Peptides were labeled with Tandem Mass Tag zero reagent (TMT^0^, Thermo Scientific Scientific) following the manufacturer’s instructions. Labeled peptides were further enriched for glycopeptides using zwitterionic hydrophilic interaction liquid chromatography resin (ZIC-HILIC, Merck, 72103-3).^17^ Glycopeptide fractions were dried using SpeedVac and resuspended in 5% acetonitrile, 0.1% trifluoroacetic acid in H_2_O.

### Glycoproteomics Analysis

The glycopeptide enriched fraction was separated using the EASY-nLC liquid chromatography (LC) system (Thermo Fisher Scientific) with a Magic C18 spray tip 15 cm×75 µm column (Bruker-Michrom, Auburn, CA). Peptides were eluted using 4% to 30% acetonitrile, 0.1% formic acid within 1 hour at a flow rate of 300 nL/min and directly analyzed with an Orbitrap Elite hybrid MS (Thermo Fisher Scientific) with electron transfer dissociation (ETD). Two MS/MS methods were used. For the alternating higher-energy collisional dissociation (HCD) and ETD MS/MS (HCD-alt-ETD) method, the top 10 ions were analyzed via HCD and ETD. For the HCD product-dependent ETD (HCD-pd-ETD) method, the top 10 ions were analyzed via HCD, and product-dependent ETD acquisition was triggered by product (oxonium) ions (m/z 163.0812 for hexose; m/z 204.0864 and 138.0554 for N-acetyl-hexosamine and its fragment ion, respectively). Glycopeptides were identified using Byonic software (version 2.9.30, Proteome Metrics).^17^ Identified glycoforms were quantified using Pinnacle software (version 1.0.65.0, Optys Tech Corporation, Boston, MA). The precursor peak area was used for quantification and normalized to the total ion current. The 2 MS runs from the same sample using different fragmentation methods were used as technical replicates for quantitation.

### Proteomics Analysis of Aortic Samples From Patients With Bicuspid Aortic Valve

Aortic samples from bicuspid aortic valve (BAV) patients and tricuspid aortic valve (TAV) patients were collected during aortic surgery at St George’s Hospital (London, UK). All procedures were approved by the Regional Ethics Committee Board (London, REC number 08/H0803/257). The diagnosis of BAV was confirmed by direct visualization of the valve phenotype. For each patient, 2 aortic specimens were collected, one from the concave area and one from the convex area of the ascending aorta and immediately frozen at −80°C for proteomic analysis (Table II in the online-only Data Supplement). The samples were anonymized directly after surgery and before the experimental procedure and analysis.

ECM proteins were extracted using the same method as described above. Twenty micrograms of proteins from GuHCl fractions were precipitated in 100% ethanol, and the pellets were resuspended in deglycosylation buffer (0.2 mol/L Tris, 0.2 mol/L sodium acetate, 0.1 mol/L EDTA, 50 mmol/L sodium phosphate, pH=6.8). The following deglycosylation enzymes were used: endo-α-N-acetylgalactosaminidase, β1,4-galactosidase, β-N-acetylglucosaminidase, α-2-3,6,8,9-Neuraminidase (all from Merck-Millipore Glycoprotein Deglycosylation Kit, 362280), Chondroitinase ABC (Sigma-Aldrich, C3667), Heparinase II (Sigma-Aldrich, H6512), and Endo-β1,4-galactosidase (Sigma-Aldrich, G6920). Samples were incubated for 1 hour at 25°C, followed by 24 hours at 37°C in agitation then dried using SpeedVac. Subsequently, samples were reconstituted in O18 water containing N-Glycosidase F (PNGase F, from the Merck-Millipore Glycoprotein Deglycosylation Kit, 362280) and incubated at 37°C with agitation for 24 hours.

After deglycosylation, proteins were denatured using 6 mol/L urea, 2 mol/L thiourea, reduced with 10 mmol/L dithiothreitol, and alkylated with 50 mmol/L iodoacetamide. Proteins were precipitated by prechilled acetone overnight, resuspended in 0.1 mol/L triethylammonium bicarbonate and digested with 0.4 µg trypsin overnight. The digestion was stopped by using 1% trifluoroacetic acid. Peptide samples were purified using a 96-well C18 spin plate (Harvard Apparatus). The eluates were frozen at −80°C for 2 hours before being immediately lyophilized (Christ Alpha 1–2 LD plus) at −55°C overnight. The peptides of BAV samples were reconstituted with 2% acetonitrile, 0.05% trifluoroacetic acid in H_2_O, and separated on a nanoflow LC system (UltiMate 3000 RSLCnano). Samples were injected onto a trap column (Acclaim PepMap100 C18 Trap, 5 mm×300 µm) at a flow rate of 25 µL/min for 3 minutes, using 2% acetonitrile, 0.1% formic acid in H_2_O. The following LC gradient was then used to separate the peptides at 300 nL/min: 0 to 10 minutes, 2% to 10% B; 10 to 200 minutes, 10% to 30% B; 200 to 210 minutes, 30% to 40% B; 210 to 220 minutes, 99% B, 220 to 240 minutes 2% B, where A=0.1% formic acid in H_2_O, B=80% acetonitrile, 0.1% formic acid in H_2_O. The reverse phase column (Acclaim PepMap100 C18, 50 cm×75 µm) was set at 40°C and coupled to a nanospray source (Picoview, New Objective, US). Spectra were collected from a Thermo Q Exactive HF Mass Spectrometer (QE HF MS, Thermo Fisher Scientific) using full MS mode over the mass-to-charge (m/z) range 350 to 1600. Data-dependent MS/MS scan was performed on the most abundant 15 ions in each full MS scan with dynamic exclusion enabled.

Proteome Discoverer (version 2.2.0.388, Thermo Fisher Scientific) was used to search raw data files against the human database (UniProtKB/Swiss-Prot version 2018_05, 20349 protein entries) using Mascot (version 2.6.0, Matrix Science). The mass tolerance was set at 10 ppm for precursor ions and 20 mmu for fragment ions. Trypsin was used as the enzyme with 2 missed cleavages being allowed. Carbamidomethylation of cysteine was chosen as a fixed modification; oxidation of methionine, lysine and proline, and deamidation of asparagine in the presence of O18 water were chosen as variable modifications. Scaffold (version 4.8.7, Proteome Software Inc, Portland, OR) was used to validate MS/MS-based peptide and protein identifications with the following filters: a peptide probability of >95.0%, a protein probability of >99.0%, and at least 2 independent peptides per protein. The normalized total ion current was used for quantification.^24^

Selected proteins were quantified in the BAV samples, as well as TAV samples, using multiple reaction monitoring on an UltiMate 3000 RSLCnano system with the same setup, coupled to a triple quadrupole MS (TSQ Vantage, Thermo Fisher Scientific). The TSQ Vantage was set in positive ion mode with ion transfer capillary temperature at 275°C and spray voltage at 3200 V. Both Q1 and Q3 were set at unit mass resolution (0.7Th FWHM), and argon was used as the collision gas at 1.0 mTorr. For the scheduled multiple reaction monitoring method, a retention time window of 8 minutes and a cycle time of 1.43 seconds were used. Skyline software (version 3.1, MacCoss Lab Software) was used to generate the transition list with predicted collision energies as well as optimized transitions (Table XI in the online-only Data Supplement). Multiple reaction monitoring data were analyzed by Skyline. All peaks were reviewed and integrated manually. Total fragment peak areas were used for quantification.

### Immunoblotting

Proteins from the GuHCl and NaCl fractions were precipitated in 100% ethanol or acetone, respectively, overnight at −20°C and deglycosylated as described in BAV sample preparation method, except that PNGase F was added in parallel to the other enzymes, and the incubation was performed in one step, for 48 hours at 37°C. Deglycosylated proteins were denatured with reducing Laemmli sample buffer at 97°C for 5 minutes, separated by SDS-PAGE using 4% to 12% Bis-Tris gels (Invitrogen), transferred to nitrocellulose membranes (GE Healthcare), and subjected to immunoblotting following standard protocols. The following primary antibodies were used: MFAP4 (Abcam, ab169757), ACAN (aggrecan) neoepitope (Thermo Fisher Scientific, PA1-1746), VCAN (versican) neoepitope (Abcam, ab19345), and TGF-β1 (Abcam, ab179695) (please see the Major Resources Table in the online-only Data Supplement). Primary antibodies were detected by anti-rabbit light-chain specific secondary antibodies linked to horseradish peroxidase (Jackson ImmunoResearch Europe Ltd, 211-032-171). Protein bands were detected by enhanced chemiluminescence (GE Healthcare) on an x-ray film. The film was scanned and quantified using Image Quant TL (version 8.1, GE Healthcare).

### SMC Culture With TGF-β1

Primary human aortic SMCs (PromoCell, C-12533, 3 different donors, see Major Resources Table in the online-only Data Supplement) were cultured in DMEM with 2 mmol/L L-glutamine, 100 U/mL penicillin, 100 μg/mL streptomycin, and 20% fetal bovine serum. Murine aortic SMCs were cultured similarly, yet with DMEM/F12, from wild-type C57Bl6/J aortas after enzymatic digestion as described previously.^25^ Cell experiments were initiated with a subconfluent SMC layer at passage 6 to 10. SMCs were preincubated with serum-free medium for 24 hours, followed by a 24-hour incubation with 10 ng/mL recombinant TGF-β1 (R&D, 240-B-002) with or without 10 µmol/L of the ALKi (activin receptor-like kinase 4,5,7 inhibitor) SB 431542 (R&D, 1614) to block TGF-β type I receptors. Independent culture experiments were performed and normalized to the controls (no TGF-β1 and no ALKi) for comparison of fold change after TGF-β1 or simultaneous TGF-β1 and ALKi treatment.

### Silencing of *MFAP4* in Human Aortic SMCs

Human aortic SMCs (PromoCell, C-12533, 3 different donors) were cultured on 6-well plates (1.5×10^5^ cells/well) using SMC Growth Medium 2 (PromoCell C-22062) until 70% confluent. MFAP4 siRNA (50 nmol/L, Life Technologies, AM16708, Assay ID: 11386) and control siRNA (50 nmol/L, Life Technologies, AM4613) were transfected by using lipofectamine in different passages of different donors (2 for donor 1, 7 for donor 2, and 5 for donor 3) for 6 hours and subsequently in M199 with 100 U/mL penicillin, 100 µg/mL streptomycin, and 2.5% fetal bovine serum overnight. After replaced with complete medium (M199 with 100 U/mL penicillin, 100 µg/mL streptomycin, and 20% fetal bovine serum) and cultured for 48 hours, the cells were washed using M199 3 times, and further cultured in M199 for 24 hours. The cells were harvested for RNA extraction as detailed below. In total, 14 individual experiments using 3 different cell lines were performed for each condition. The experiment was repeated with a different MFAP4 siRNA at a lower concentration (25 nmol/L, Life Technologies, 4392420, Assay ID: s8716).

### Correction of *FBN1* in Induced Pluripotent Stem Cell-SMCs From a Patient With MFS

Dermal fibroblasts from a patient with MFS (Coriell Institute, GM21943) were reprogrammed into human induced pluripotent stem cells (iPSCs), and the mutation was corrected by CRISPR-Cas9 to generate an isogenic control.^26^ Control and Marfan iPSCs were grown on Vitronectin XF (STEMCELL Technologies) and maintained in mTeSR E8 media (STEMCELL Technologies). They were then differentiated into neural crest SMCs using the methods described previously.^27,28^ After differentiation, the resulting SMCs were maintained in DMEM supplemented with 10% fetal bovine serum for at least 2 weeks before seeding onto UniFlex Culture Plates (FlexCell International Corporation). After 4 days, the SMCs were stretched for 48 hours using a Cyclic Stress Unit (FX5000 Tension System, FlexCell International Corporation), using a cyclic sine wave (60 cycles/min) and 10% elongation.

### Gene Expression Analysis

Total RNA was isolated from aortic tissues (n=5 for control group and n=11 for MFS group), human aortic SMCs from the TGF-β1 experiment (3 different donors with 6 technical replicates for control, TGF-β1, and TGF-β1+ALKi groups), human aortic SMCs from the siRNA experiment (3 different donors with 14 technical replicates for both control siRNA and MFAP4 siRNA group) and iPSC-SMCs using the miRNeasy Mini kit (Qiagen) as described previously.^29^ Briefly, 700 μL of QIAzol reagent was added to samples. A homogenization step in a Precellys 24-Dual homogenizer (Peqlab) using Lysing Matrix D ceramic beads (MP Biomedicals) was performed for tissues only. After a brief incubation at room temperature, 140 μL of chloroform was added, and the solution was mixed vigorously. The samples were then centrifuged at 16000*g* for 15 minutes at 4°C. The aqueous phase was carefully transferred to a new tube and mixed with 1.5 volumes of 100% ethanol. The samples were then applied directly to columns and washed according to the manufacturer’s instructions. Total RNA was eluted in 25 μL of nuclease-free H_2_O. RNA was reverse-transcribed into cDNA using the High Capacity Reverse Transcriptase kit (Life Technologies). Quantitative polymerase chain reaction (qPCR) was performed using Taqman Assays to assess the gene expression levels with GAPDH as a normalization control.

For experiments concerning TGF-β1 stimulation of mouse aortic SMCs, RNA was isolated from SMCs with the Aurumn total RNA mini kit (BioRad) according to the manufacturer’s instructions. cDNA synthesis was performed with the iScript cDNA synthesis kit (BioRad). Specific primers were designed using Primer3 to analyze *MFAP4* gene expression. The 60S acidic ribosomal protein P0 (*Rplp0*) gene was used for normalization. Quantitative real-time PCR was performed on a LightCycler 480 system (Roche) using the SensiFAST SYBR No-ROX Kit (Bioline). All primers used for qPCR analysis are listed in Major Resources Tables in the online-only Data Supplement.

### Histological Analysis and Immunohistochemistry

Alcian blue staining was performed in aortic tissues from patients with MFS (n=27) and controls (n=7) to visualize acidic polysaccharide accumulation, such as glycosaminoglycans and quantified (corrected for medial area) with QWin software (version 3.0, Leica Microsystem). Nuclear Fast red was used as counterstain for nuclei. Immunohistochemistry was performed on 7 µm thick formalin-fixed paraffin embedded aortic tissue sections from patients with MFS. Sections were first deparaffinized in xylene followed by rehydration. To quench the endogenous peroxidases, sections were treated with 2% hydrogen peroxide for 20 minutes, followed by a 30 minutes incubation with 10% normal goat serum and 1% bovine serum albumin diluted in Tris-buffered saline to block nonspecific binding. The sections were then incubated overnight at 4°C with a polyclonal rabbit anti-MFAP4 antibody (Abcam, ab80319) diluted 1:500 in Tris-buffered saline. Control sections were treated with rabbit IgG. The following day the slides were incubated for 30 minutes with an horseradish peroxidase-conjugated anti-rabbit immunoglobulin polymer (Immunologic) diluted 1:1 with Tris-buffered saline followed by development with bright 3,3′-diaminobenzidine (Immunologic). The sections were counterstained with Gill’s hematoxylin and mounted with Pertex.

### MFAP4 Quantification in Plasma Samples From Patients With MFS

For the analysis of plasma protein levels of MFAP4, we used the same samples as described earlier (n=96).^21^ A human MFAP4 ELISA kit (Elabscience, E-EL-H0581) was used to determine MFAP4 protein levels in plasma following the manufacturer’s instructions. Briefly, plasma samples from patients with MFS were diluted 1:50 with the standard and sample diluent provided. The samples were incubated for 90 minutes at 37°C in the provided wells coated with anti-MFAP4 antibody. Second, the samples were removed followed by a 1-hour incubation at 37°C with the anti-MFAP4 biotinylated detection antibody. After washing, a 30 minutes incubation with an horseradish peroxidase conjugate was followed by another washing step. Last, a horseradish peroxidase substrate was added for 5 minutes with subsequent addition of the stop solution. Optical density was measured at a wavelength of 450 nm with the ELx808 ultra microplate reader (BioTek Instruments, Inc, Winooski, VT).

### Ultrasonic and MRI Measurements of the Aorta

In the COMPARE patients, the aortic root diameter was measured at time of inclusion and during follow-up yearly by echocardiography. The aortic growth rate (mm/y) was determined after 38 months of follow-up, by subtracting the root diameter at baseline from the most recent diameter, dividing this by the COMPARE follow-up period in years. Occurrence of events, such as prophylactic aortic surgery or aortic dissection, were recorded during the follow-up of 68 months. Aortic distensibility at 4 levels throughout the aorta was calculated: (1) the ascending aorta, (2) the descending thoracic aorta at the level of the bifurcation of the pulmonary artery, (3) the descending aorta at the level of the diaphragm, and (4) the abdominal aorta just above the bifurcation. Measurements were performed by a single analyst using cine magnetic resonance images, as previously described.^5,21,30^

### Statistical Analysis

Continuous data are presented as mean with SD or median with range, as described in the figure legends. Categorical variables were summarized using proportions.

The quantities of the proteins/peptides were scaled using log2 transformation. The data sets were further filtered to keep only measurements with <30% missing values or with >90% missing values one of the examined conditions and <10% for the rest of the conditions. In the latter case, missing values of the condition which presented >90% of missing values were imputed with zeros. All remaining missing values were imputed with KNN-Impute method with k equal to the minimum among 20 and the sample size of the phenotype with the fewest samples.

Before performing statistical comparison, we performed the Shapiro-Wilk normality test to check whether the relative quantities were normally distributed.

For continuous variables with a normal distribution, differences between groups were analyzed with a Student *t* test. When the data were not normally distributed, nonparametric methods were used. In specific, for paired comparisons, the Wilcoxon signed-rank test and Mann-Whitney *U* test for unpaired comparisons were used. *P* values were adjusted using the Benjamini-Hochberg method, and a threshold of 0.05 was used for the adjusted *P* values to infer statistically significant changes.

Differences in categorical variables were analyzed with a χ^2^ or Fisher Exact test. The Kaplan-Meier analysis was used for comparisons of event-free survival. Statistical analysis was performed using SPSS software (version 24.0, IBM) or Python (version 3.7.2) scripts. Graphs were created with Graphpad Prism software (version 7.04).

## Results

### Glycoproteomics of the Aortic ECM in Patients With MFS

**Figure 1.**
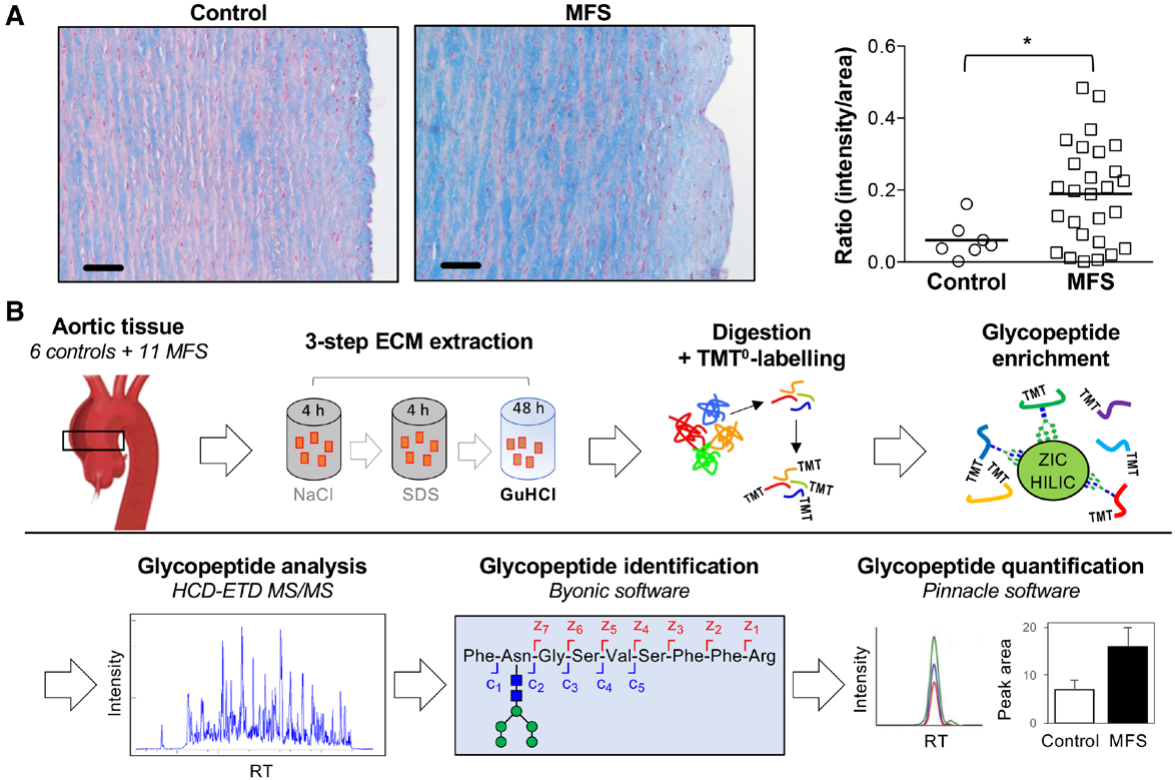
**Glycoprotein content in aneurysms of patients with Marfan syndrome (MFS).** **A**, Alcian blue staining of aortic tissue sections from patients with MFS (n=27) and controls (n=7). Stronger blue staining indicates buildup of glycoproteins as well as mucopolysaccharides. Scale bars are 50µm. *P<0.05 by Student *t* test. **B**, Glycoproteomics workflow showing the 3-step extraction, enrichment and liquid chromatography-tandem mass spectrometry (LC-MS/MS) analysis of extracellular matrix (ECM)-derived glycopeptides from thoracic aortic aneurysm tissue of control (n=6) and MFS patients (n=11). ETD indicates electron transfer dissociation; and HCD, higher-energy collisional dissociation; RT, retention time; and SDS, sodium dodecyl sulphate.

**Figure 2.**
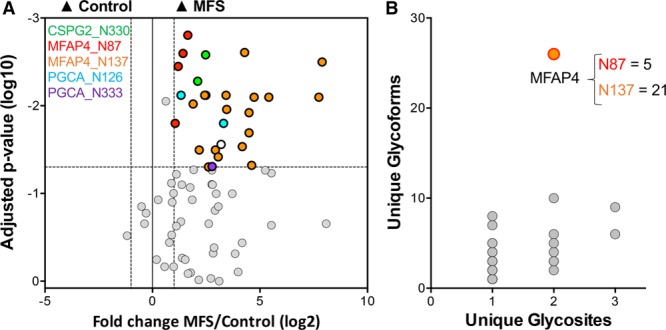
**Glycoproteomic analysis of the aortic extracellular matrix (ECM) in aneurysms of patients with Marfan syndrome (MFS).** **A**, Volcano plot showing the difference in abundance of glycopeptides derived from ECM proteins in thoracic aneurysms of MFS vs control patients. Versican (CSPG2), MFAP4 (microfibril-associated glycoprotein 4), and PGCA (aggrecan) have been highlighted with their accompanying glycosylation sites. Different data points for the same color represent alternative glycans at the same glycosylation site. Mann-Whitney *U* tests were used with multiple testing adjustment. Thresholds for significance are indicated by dotted lines (*P*<0.05 and fold change <0.5 or >2). **B**, All identified glycoproteins are plotted according to their number of unique glycosites and number of unique glycoforms. MFAP4 possesses the highest number of unique glycoforms.

Enhanced glycoprotein levels in the aorta of patients with MFS were demonstrated by Alcian blue staining, compared with aneurysmal patients without MFS (Figure 1A). For glycoproteomics, thoracic aortic aneurysm samples from patients with and without MFS (n=11 and n=6, respectively, Table I in the online-only Data Supplement) were compared. The ECM was extracted using our 3-step extraction protocol. The glycopeptides in the GuHCl fraction were enriched and analyzed by a glycoproteomic approach on an Orbitrap Elite MS (Figure 1B). After manual validation of the identified glycopeptides, a total of 215 unique spectra, corresponding to 35 different glycoproteins, were confirmed. A total of 141 glycoforms on 47 glycosites were identified (Table III in the online-only Data Supplement). Among these, 119 unique spectra were deemed suitable for further quantitative analysis using their precursor peak area. In total, 38 N-linked glycopeptides from 8 ECM proteins were differentially expressed. The most significant differences between MFS and non-MFS were observed for MFAP4, as well as for the 2 large aggregating proteoglycans PGCA (aggrecan) and CSPG2 (versican; Figure 2A, Table IV in the online-only Data Supplement). MFAP4 has 2 N-glycosites at asparagine (N) positions N87 and N137. Interestingly, only 5 glycoforms were identified at N87 compared with 21 glycoforms at N137. Compared with all other identified glycoproteins, MFAP4 exhibited the greatest number of unique glycoforms overall (Figure 2B).

### Glycan Heterogeneity of MFAP4

**Figure 3.**
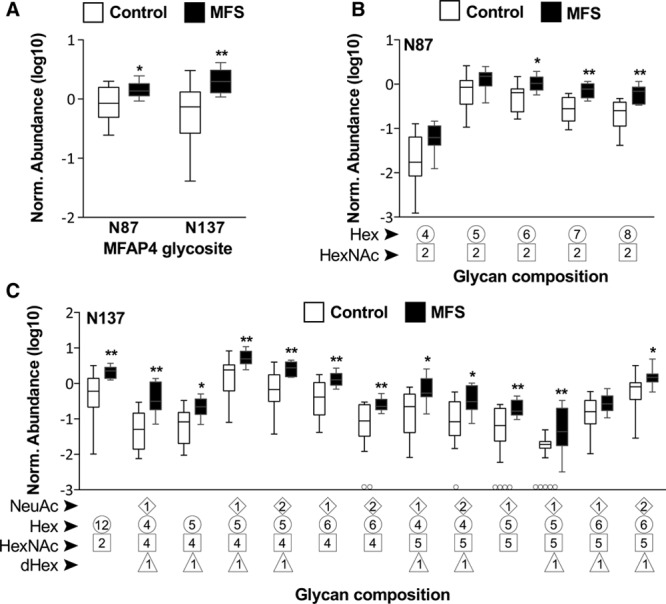
**Increased MFAP4 (microfibril-associated glycoprotein 4) glycopeptides in patients with Marfan syndrome (MFS).** **A**, Quantification of the overall glycopeptide abundance on the 2 MFAP4 glycosites between MFS and control groups. **B**, Quantification of glycopeptides with different high-mannose glycans on N87 of MFAP4. **C**, Quantification of glycopeptides with different glycans on N137. Box and whisker plots represent the second and third quartiles and error bars expand from the 10th to the 90th percentiles. Circled data points along the x-axis represent no detection (n=6 for control and n=11 for MFS, with duplicate liquid chromatography-tandem mass spectrometry [LC-MS/MS] runs for each sample). dHex indicates L-Fucose; Hex, Hexose; HexNAc, N-Acetyl-hexosamine; and NeuAc, N-Acetylneuraminic acid. *P<0.05, **P<0.01 by Mann-Whitney *U* test with multiple testing adjustment using Benjamini-Hochberg method.

Next, we focused on the glycoform diversity present at the 2 glycosites of MFAP4. Representative MS/MS spectra from glycopeptides derived from the N87 and N137 sites are provided (Figure I in the online-only Data Supplement). We discovered that glycopeptides corresponding to both glycosites displayed significantly higher abundance in the aortic ECM of MFS patients compared to non-MFS controls (Figure 3A). Notably, glycans linked to N87 all belonged to the high-mannose class. Of these, glycopeptides consisting of 8 to 10 monosaccharides were increased in samples from patients with MFS while no significant changes were observed for glycopeptides with 6 or 7 monosaccharides (Figure 3B). More diverse glycans were attached to the N137 glycosite. Contrary to N87, only one out of 13 glycans was high-mannose, and the corresponding glycopeptide was increased in patients with MFS. All other glycans were of a hybrid/complex class, of which 11 corresponding glycopeptides were significantly increased in patients with MFS (Figure 3C).

### *MFAP4* Gene Expression in Patients With MFS

**Figure 4.**
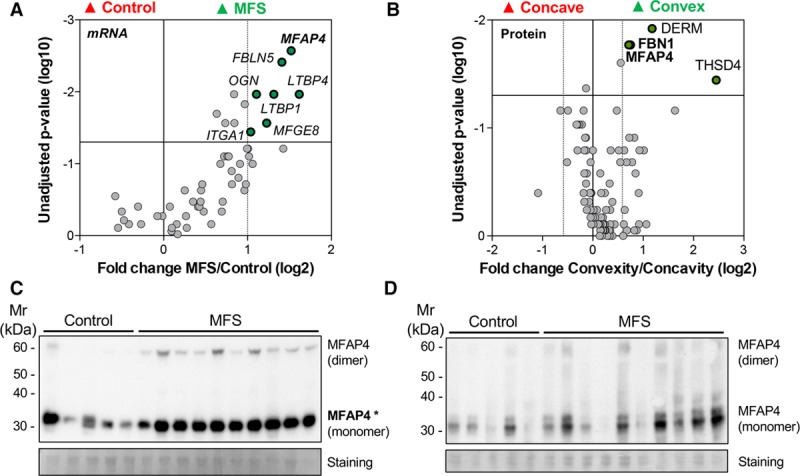
**MFAP4 (microfibril-associated glycoprotein 4) expression is upregulated in Marfan syndrome (MFS).** **A**, Volcano plot showing the differences in gene expression of selected extracellular matrix (ECM) proteins between aneurysmal thoracic aortic tissue from patients with MFS (n=10) and controls (n=5). *P* values were calculated using Mann-Whitney *U* test. **B**, Volcano plot showing the ECM protein differences between concave and convex area of the same thoracic aorta from nonaneurysmal BAV patients (n=8). *P* values were calculated using Wilcoxon signed-rank test. Immunoblotting for MFAP4 in the NaCl (**C**) and guanidine hydrochloride (GuHCl) samples (**D**) between aneurysmal thoracic aortic tissue from patients with MFS (n=10) and controls (n=5). Please note the control sample in lane 1 with high MFAP4 in the NaCl fraction is from a patient with a TGFBR1 (transforming growth factor β receptor type 1) mutation. FBN1 indicates fibrillin-1. Student *t* test: *P<0.05.

To complement the proteomics data, we determined the corresponding levels of gene expression for a select panel of ECM proteins. From a subset of the same aortic specimens used for glycoproteomics analysis (n=5 for control group and n=11 for MFS group), the mRNA levels of 55 ECM protein genes were quantified by qPCR. The mRNA levels of several elastin fiber-related proteins were significantly higher in MFS patients compared with controls, including fibulin-5 (*FBLN5*), latent-transforming growth factor-β-binding protein 1 (*LTBP1*), and 4 (*LTBP4*) as well as *MFAP4* (Figure 4A, Table V in the online-only Data Supplement).

### MFAP4 in Patients With BAV

The presence of a BAV is a predisposing factor for the development of thoracic aortic aneurysms.^31^ To explore whether MFAP4 may be upregulated in early stages of this disease, we obtained aortic specimens from patients with BAV not presenting with aortic aneurysms (Table II in the online-only Data Supplement). Using proteomics, we compared the ECM of the convex and concave area within the same nonaneurysmal aortas (n=8). The convex area of the ascending aorta is more prone to aneurysmal development, most likely because of shear stress exposure, which is altered in BAV patients compared with individuals with TAV.^32^ Interestingly, we found that although no aortic aneurysms were present, the abundance of MFAP4 protein was higher in the convex area when analyzed by LC-MS/MS. Notably, the increase in MFAP4 was accompanied by a rise in FBN1 (Figure 4B, Table VI in the online-only Data Supplement). MFAP4 and FBN1 were validated by a targeted multiple reaction monitoring method in GuHCl fraction of concave and convex samples of aortas from both nonaneurysmal BAV patients and nonaneurysmal TAV patients (n=7). These 2 proteins were significantly increased (MFAP4: 1.54-fold increase, *P*=0.025; FBN1: 1.67-fold increase, *P*=0.029) in the convex area of BAV patients compared to the concave area, but this was not the case in TAV patients (MFAP4: 1.35-fold increase, *P*=0.231; FBN1: 2.19-fold increase, *P*=0.075; Figure II and Table VII in the online-only Data Supplement).

### MFAP4, Proteoglycans, and ADAMTS Proteases in MFS

Using an N-terminal antibody for MFAP4, we confirmed an increase in MFAP4 levels in the NaCl fraction (Figure 4C) but not in the GuHCl fraction (Figure 4D) of MFS aortic tissue. Alongside MFAP4, the large aggregating proteoglycans CSPG2 and PGCA were increased in the glycopeptide analysis without corresponding changes in gene expression. Large aggregating proteoglycans are cleaved by members of the ADAMTS (a disintegrin and metalloproteinase with thrombospondin motifs) family. Two anti-neoepitope antibodies were used to detect ADAMTS-specific cleavage of aggrecan and versican in MFS: the aggrecan neoepitope antibody detects the neoepitope sequence NITEGE, generated by ADAMTS-cleavage at the position Glu392-Ala393 in the core protein; the versican neoepitope antibody detects the neoepitope sequence DPEAAE, generated by ADAMTS-cleavage at the position Glu441-Ala442 of the protein core of versican isoform V1.^33^ Detection of both neoepitopes was higher in the GuHCl fraction of patients with MFS compared with control but only versican reached statistical significance. Interestingly, levels of TGF-β1 remained unaltered between MFS and control patients (Figure III in the online-only Data Supplement).

### Regulation of MFAP4 Expression

**Figure 5.**
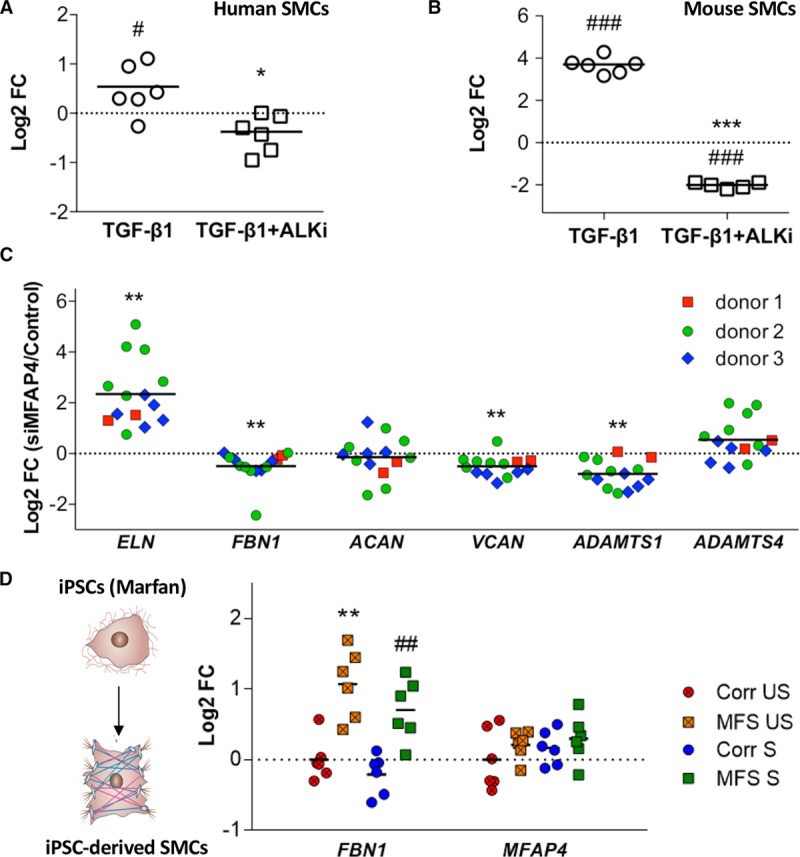
**Changes of MFAP4 (microfibril-associated glycoprotein 4) in relation to TGF-β1 (transforming growth factor β receptor type 1), ELN (elastin), and FBN1 (fibrillin-1).** *MFAP4* gene expression was quantified in human (**A**) and murine (**B**) aortic smooth muscle cell (SMCs) after incubation with TGF-β1 in the absence or presence of an ALKi (activin-like kinase 4,5,7 inhibitor). Data points are individual replicates with horizontal bars representing the average. The dashed line represents the control (no TGF-β1, no ALKi) average, which was set to 1. Log2 fold change (FC) as compared to control group. *P* values were calculated using Student *t* test. *P<0.05, ***P<0.001 vs TGF-β1 group; #*P*<0.05, ###*P*<0.001 vs Control group. **C**, Silencing of MFAP4 by siRNA in human aortic SMCs (from 3 different donors) resulted in mRNA changes of various extracellular matrix (ECM) proteins and members of the ADAMTS (a disintegrin and metalloproteinase with thrombospondin motifs) family. Gene expression was normalized to control group (dotted line). Wilcoxon signed-rank test was performed and adjusted *P* value was calculated. **P<0.01. **D**, SMCs were differentiated from iPSCs (induced pluripotent stem cell) derived from a Marfan syndrome (MFS) patient and the *FBN1* mutation was corrected by CRISPR/Cas9 (Corr). Cells were cultured under unstretched (US) or stretched (S) conditions. Gene expression was normalized to corrected unstretched (Corr US) group (dotted line). In the absence of elastin expression, no changes in MFAP4 were observed. Unpaired Student *t* test was performed and adjusted *P* value was calculated. *P<0.05, **P<0.01 compared to Corr US group; ##*P*<0.01 compared to Corr S group.

TGF-β1 has been implicated in the pathophysiology of aortic aneurysms in patients with and without MFS, via both direct genetic mutations in the TGF-β pathway (Loeys-Dietz Syndrome) and as a consequence of *FBN1* mutations (MFS) leading to dysregulated TGF-β1 levels in the pericellular environment and excessive TGF-β1 signaling through ALK receptors. In human and murine aortic SMCs, administration of TGF-β1 upregulated *MFAP4* gene expression. This effect was reversed by the presence of an ALK receptor inhibitor (Figure 5A and 5B, Table VIII in the online-only Data Supplement). Therefore, TGF-β1 may function as a transcriptional regulator of MFAP4 in arterial SMCs and contribute to MFAP4 regulation during aortopathy.

### Silencing of *MFAP4* in Human Aortic SMCs

To investigate the functional consequences of alterations in *MFAP4* expression, we used a gene silencing approach to inhibit *MFAP4* translation in cultured human aortic SMCs. Efficient knockdown of *MFAP4* (Log2 fold change =−4.34 compared with control, *P*=0.004) resulted in increased expression of elastin (*ELN*, Log2 fold change =2.35, *P*=0.004) and reduced expression of *FBN1* (Log2 fold change =−0.50, *P*=0.005). Interestingly, silencing of *MFAP4* also significantly attenuated the expression of versican (*VCAN*, Log2 fold change =−0.50, *P*=0.007), whereas aggrecan (*ACAN*) expression was variable between different donors (Figure 5C, Figure IV and Table IX in the online-only Data Supplement). We have recently highlighted the importance of aggrecan and the ADAMTS family in stent-induced vascular injury.^34^ Moreover, ADAMTS family members have been implicated in murine thoracic aortic aneurysm formation.^24,35^ To further investigate whether MFAP4 may be linked to ADAMTS enzymes, we tested the expression levels of *ADAMTS-1* and *-4*. While *ADAMTS-1* (Log2 fold change =−0.80, *P*=0.004) was significantly decreased after MFAP4 knockdown, *ADAMTS-4* (Log2 fold change =0.54, *P*=0.055) appeared to be marginally increased (Figure 5C). Unlike ADAMTS proteases, the expression of 3 MMPs (matrix metalloproteinases), namely *MMP2* (collagenase), *MMP9* (collagenase), and *MMP12* (elastase), did not show significant changes in response to MFAP4 inhibition (Table IX in the online-only Data Supplement).

### Induced Pluripotent Stem Cells-Derived SMCs

It is worth noting that apart from MFAP4 other elastic fiber-interacting proteins were increased as well in gene expression data from MFS aortas, such as *FBLN5*, *LTBP* 1 and 4, and lactadherin (*MFGE8*; Figure 4A). To further investigate the functional links between *FBN1*, *ELN*, and *MFAP4*, dermal fibroblasts of a patient with MFS were reprogrammed into iPSCs. The *FBN1* gene mutation was corrected using CRISPR-Cas9 to provide an isogenic control.^26^ Both the *FBN1*-mutant iPSCs and *FBN1*-corrected iPSCs were further differentiated into neural crest vascular SMCs.^26^ Gene expression was assessed by qPCR under static and stretch conditions. *FBN1* expression was higher in the SMCs from the patients with MFS than in the corrected isogenic control under both conditions (*P*<0.01 for both comparison). As elastin expression was undetectable in these iPSC-derived SMCs (Ct >35 cycles), expression levels of *MFAP4* were unchanged (Figure 5D).

### Localization of MFAP4

**Figure 6.**
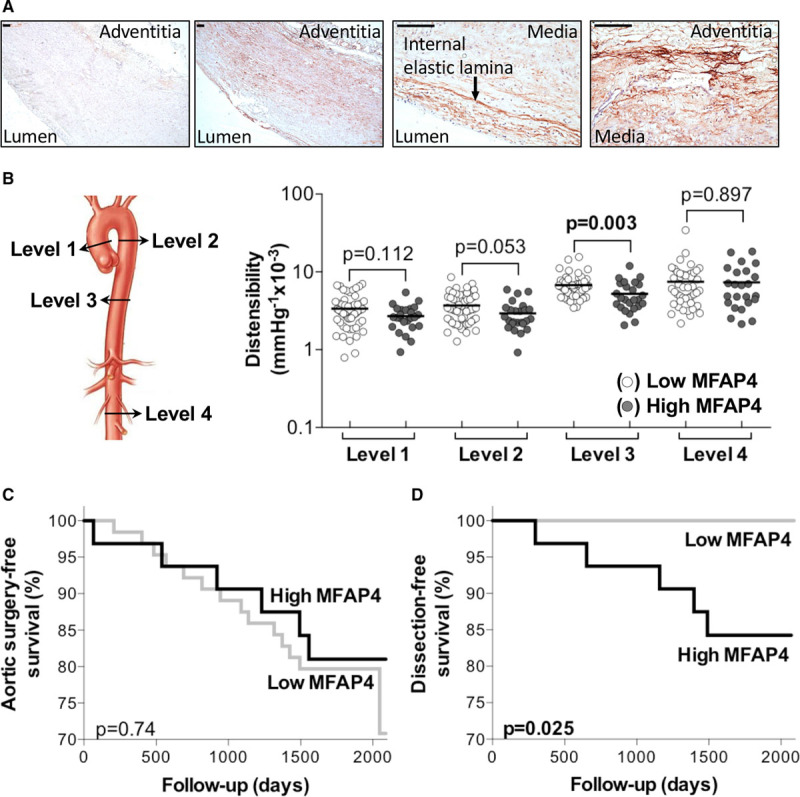
**Plasma levels of MFAP4 (microfibril-associated glycoprotein 4) predict the occurrence of type B dissections.** **A**, Aortic tissues from patients with Marfan syndrome (MFS) were stained for MFAP4 (red-brown) with hematoxylin (blue) nuclear counterstaining. MFAP4 is localized throughout the vessel wall with prominent staining of the internal elastic lamina (arrow), the adventitia and the medial layer. Scale bars are 100 µm. **B**, The distensibility measurements in patients with MFS are shown at the ascending (level 1; n=54 vs n=25), proximal descending (level 2; n=53 vs n=25), distal descending (level 3; n=53 vs n=25) thoracic aorta and abdominal aorta (level 4; n=46 vs n=24), according to low (bottom 2 tertiles) vs high (upper tertile) plasma MFAP4 concentrations. Survival curve showing the aortic surgery incidence (**C**) and aortic dissection incidence (**D**) in the 68-mo follow-up period in MFS patients with low (bottom 2 tertiles; n=64) vs high (upper tertile; n=32) plasma MFAP4 levels. High plasma MFAP4 levels were associated with a high dissection incidence. The Kaplan-Meier analysis was used for comparisons of event-free survival.

To determine the localization of MFAP4, we performed immunostaining of histological sections from aortas of patients with MFS. MFAP4 colocalized with the internal elastic lamina in the aortic wall and in the adventitia at the external elastic lamina and possibly also with collagen fibrils (Figure 6A).

### MFAP4 Plasma Levels Associate With Aortic Distensibility and Dissection in MFS

In addition to its expression in the aortic ECM, MFAP4 was detectable in plasma obtained from a subset of patients with MFS from the COMPARE trial (n=96).^21^ To determine whether plasma MFAP4 associates with aortic events, the 96 patients with MFS were subdivided into 3 equal tertiles (lower, middle, and upper tertile) based on their MFAP4 protein levels. Patient demographics of each tertile are summarized in Table X in the online-only Data Supplement. No significant differences were observed for sex, age, usage of blood pressure lowering agents at time of inclusion or the percentage of patients who have undergone prophylactic aortic surgery before or during follow-up. Twenty aortic root replacements and 5 type B dissections (ie, affecting the descending thoracic aorta) occurred during the 68 months follow-up period. Interestingly, all type B dissections occurred in patients from the upper tertile (high plasma MFAP4). Because there was no significant difference in plasma MFAP4 levels between patients with or without previous aortic root replacement, MFAP4 seems to be independent from this well-known risk factor for type B dissections. Moreover, high plasma MFAP4 levels or type B dissections were not related to any of the blood pressure lowering drugs used.

To study local aorta characteristics, the aortic distensibility, which is a measure for aortic wall elasticity, was measured at 4 locations: the ascending aorta (level 1, n=79), the descending thoracic aorta at the level of the bifurcation of the pulmonary artery (level 2, n=78), the descending aorta at the level of the diaphragm (level 3, n=78), and the abdominal aorta just above the bifurcation (level 4, n=70). Lower distensibility was observed at the proximal (level 2, *P*=0.053) and the distal (level 3, *P*=0.003) descending thoracic aorta in patients with high plasma MFAP4 levels (the upper tertile; Figure 6B, Table X in the online-only Data Supplement). These are the 2 locations where type B dissections occur in patients with MFS. While in our previous study high plasma TGF-β1 correlated to the incidence of prophylactic aortic root surgery during follow-up (as surrogate for aortic root growth),^21^ high plasma MFAP4 was not associated with prophylactic aortic root surgery (*P*=0.74, Figure 6C). However, it was associated with a reduction in dissection-free survival in this cohort (*P*=0.025, Figure 6D).

## Discussion

ECM degradation and remodeling are hallmarks of aortic aneurysm pathology.^36^ Previously, we established an extraction methodology for ECM proteins and performed the first ECM proteomic comparison between control human aortas and abdominal aortic aneurysms.^18,36^ To further study the involvement of ECM proteins in aortic aneurysm formation, we provide a glycoproteomics characterization of MFS, a condition strongly associated with premature aortopathy. We provide evidence for altered glycosylation of MFAP4, a protein involved in elastic fiber assembly. Thus, changes in ECM proteins in MFS are not confined to abundance only, but involve PTMs, which have been far less studied. To substantiate the clinical relevance of MFAP4 in patients with MFS, we demonstrate that increased plasma levels of MFAP4 associate with reduced aortic distensibility and a higher incidence of type B dissection.

### Glycosylation of MFAP4 in the Human Aorta

Glycosylation is one of the most prominent and complex PTMs. Glycans have the ability to alter the mass, charge, and electrophoretic properties of proteins, influencing protein folding, stability, activity, distribution, targeting, and recognition.^37^ Moreover, gain-in-glycosylation mutations have been shown to comprise a significant number of pathogenic mutations, presenting in pathologies such as MFS.^38^ Several glycopeptides displayed altered levels in patients with MFS, the highest number of which were glycopeptides from MFAP4. MFAP4 contains 2 N-glycosylation sites and evidence for MFAP4 glycosylation was previously established by N-terminal sequencing of the recombinant glycoprotein.^39^ The functional impact of MFAP glycosylation changes has been suggested previously, since this may result in a more negatively charged glycoprotein, which confers a stronger electrostatic affinity to positively charged proteins, such as tropoelastin.^40^ A greater negative charge in MFAP4 could be achieved on accumulation of sialic acid (NeuAc), the only glycan-building monomer bearing a net negative charge.^41^ Moreover, the presence of sialic acid is restricted to glycans attached to the N137 position of MFAP4, and the majority of corresponding glycopeptides were increased in the aortic ECM of MFS patients. Therefore, certain glycoforms might be favored at specific glycosylation sites, which could underlie a compensatory response that enhances the interaction of MFAP4 with elastin fibers during aneurysmal development to strengthen the integrity of the vessel wall. This concept of adaptive regulation in response to changes in elastic fiber content has previously been observed in a proteomic analysis of aortic aneurysms.^42^

### MFAP4 in Elastic Fibre Composition

Elastic fiber breakage is a key characteristic of aortopathy, as which occurs in MFS.^43^ Underlying elastin microfibrils contain FBN1, LTBPs, fibulins, MFAPs, and other microfibril-associated glycoproteins.^44^ MFAP4 mainly localizes at sites rich in elastic fibers and is known to be involved in their assembly and maintenance.^45^ It is noteworthy that mRNA levels of *MFAP4* are higher in MFS compared with control patients and also that mRNA levels of *ELN* were upregulated when *MFAP4* knockdown was performed in human aortic SMCs. Interestingly, a 2-fold increase in MFAP4 abundance in the ascending aorta of patients with MFS has been previously reported using difference in-gel electrophoresis.^46^ The coordinated expression with *FBN1* may be expected, given that MFAP4 has been demonstrated to bind and colocalize with FBN1 to aid in elastin fiber formation.^45^ However, correction of the *FBN1* mutation in iPSC-derived vascular SMCs had no direct effect on *MFAP4* levels, as these SMCs produced little elastin in culture. In some iPSC-derived SMCs with detectable elastin expression (Ct <35 cycles), however, a corresponding increase in MFAP4 expression was observed (data not shown). Thus, the rise in MFAP4 is a compensatory response to the abnormal elastin formation in patients with MFS rather than a direct consequence of the *FBN1* mutation. Apart from MFAP4, compensatory changes were also observed for other elastin accessory proteins at the predilection site of nonaneurysmal aortas from BAV patients. While these early compensatory mechanisms may transiently improve interactions between fiber components, they might constitute defective long-term remodeling of the vascular ECM. It is noteworthy that MFAP4 has been shown to promote SMC proliferation and migration during vascular injury,^45,47^ supporting the concept that ECM remodeling also provides direct signals to cells within the ECM.

### MFAP4 and TGF-β1 Signaling

It has been suggested that mutant FBN1 expressed in the ECM leads to excessive TGF-β signaling, thereby coordinating the pathophysiological sequelae of MFS.^48^ This hypothesis has recently been challenged by clinical trials showing no superiority of losartan, an angiotensin II type 1 receptor antagonist and inhibitor of TGF-β production, over or alongside conventional β-blocker therapy.^6–8^ In arterial SMCs from humans and mice, TGF-β1 was a potent inducer of *MFAP4* expression. However, plasma TGF-β1 and plasma MFAP4 each predicted different aortic events in patients with MFS, suggesting that there will be additional inducers of MFAP4 to be discovered. In contrast to other studies,^49–51^ we failed to observe a significant change in TGF-β1 protein levels in the aortic ECM of patients with MFS versus controls.

### MFAP4 and Proteoglycan Turnover

Changes in the large aggregating proteoglycans versican and aggrecan at the glycopeptide level accompanied those observed for MFAP4 in patients with MFS. Our laboratory has previously highlighted the role of ADAMTS proteases in the proteolysis of aggrecan and versican in the vasculature.^34^ In patients with MFS, we observed an increase in the ADAMTS-mediated cleavage product of versican. This may reflect a buildup of proteoglycan substrate, which has been shown to occur in patients with thoracic aortic aneurysm or dissection, including those with MFS.^52^ Notably, our experiments showed for the first time that a reduction of *MFAP4* in SMCs leads to a parallel reduction in the expression of versican, as well as several different changes in the ADAMTS enzyme family. Changes in ECM may act as signaling mechanisms affecting *ADAMTS* expression and activity on the large aggregating proteoglycans. Reduced expression of ADAMTS-1 in mice and humans has previously been associated with MFS-related aortopathy,^35^ but our most recent data point towards a greater role for ADAMTS-5, at least in mice.^25^ The contribution of proteoglycans and their remodeling to the pathology of MFS in aortas will require further investigation, especially since mutations in ADAMTS family members were found to phenocopy genetic disorders caused by mutations affecting *FBN1*.^53,54^

### MFAP4 as Plasma Biomarker for Vascular Remodeling

In the present study, we demonstrated that MFAP4 is abundant in the aortic vessel wall of patients with MFS and that type B dissections occurred during follow-up in the patients with high plasma MFAP4. Higher plasma MFAP4 levels also associated with a lower descending thoracic aorta distensibility. The association of aortic distensibility of the thoracic descending aorta and type B dissections has been made previously.^5^ It is especially interesting that MFAP4 is not associated with prophylactic aortic root surgery, which is a known risk factor for type B dissections. Plasma TGF-β1 levels associate with aortic root growth and, therefore, may predict prophylactic aortic root surgery to prevent type A dissections,^21^ whereas plasma MFAP4 levels seem related to reduced distensibility of the descending aorta and predict the probability of a type B dissection. Although these findings await confirmation in larger cohorts, MFAP4 is an interesting candidate biomarker to identify patients with MFS at higher risk for type B dissection, who could be monitored more closely.

### Conclusions

The majority of patients with MFS have a greater propensity to experience thoracic aortic aneurysms and dissections. Until now, no in-depth glycoproteomics characterization of the vascular ECM in patients with MFS has been performed. Our glycoproteomics analysis revealed that MFAP4 glycosylation is enhanced in MFS patients with aortic aneurysms compared with aneurysms in patients without MFS. In addition to its altered glycosylation profile in MFS, knockdown of *MFAP4* expression in aortic SMCs also had a profound impact on the expression of key ECM components such as elastin and FBN1 as well as ADAMTS proteases and versican. Taken together, our findings reinforce the notion of an interplay between elastic fiber-associated proteins and large aggregating proteoglycans during aortic pathology.^55^ ECM remodeling is a hallmark of many vascular pathologies, and the application of glycoproteomics to the analysis of clinical samples provides insights into the changes in essential ECM components which occur during disease, which may lead to the discovery of novel biomarkers and potential therapeutic interventions.

## Acknowledgments

We acknowledge the COMPARE study group for their collaboration. We thank Chae Syng Lee for expert technical help and Dr Marieke Rienks for the graphical abstract.

## Sources of Funding

Dr Mayr is a British Heart Foundation (BHF) Chair Holder (CH/16/3/32406) with BHF programme grant support (RG/16/14/32397). S. Sinha is a BHF Senior Research Fellow (FS/18/46/33663) with BHF programme grant support (RG/17/5/32936). This work was supported by a joint BHF Project Grant (PG/17/48/32956). The research was also supported by National Institute of Health Research (NIHR) Biomedical Research Centre based at Guy’s and St Thomas’ NHS (National Health Service) Foundation Trust and King’s College London in partnership with King’s College Hospital and by the excellence initiative VASCage (Centre for Promoting Vascular Health in the Ageing Community), an R&D K-Centre (COMET program—Competence Centers for Excellent Technologies) funded by the Austrian Ministry for Transport, Innovation and Technology, the Austrian Ministry for Digital and Economic Affairs and the federal states Tyrol, Salzburg, and Vienna. V. de Waard is supported by Horstingstuit Foundation and Swaenenburgh Foundation. S. Wanga is supported by KNAW Ter Meulen Grant, AMC Young Talent Grant, Company of Biologists Grant, De Drie Lichten Foundation Grant, and VVAO JoKolk Grant. R. Balm is supported by private funding via the AMC Foundation. D.P. Reinhardt was supported by the Heart and Stroke Foundation of Canada (G-16-00014634) and the Canadian Institutes of Health Research (PJT-162099).

## Disclosures

None.

## Supplementary Material

## References

[1] Franken R, Heesterbeek TJ, de Waard V, Zwinderman AH, Pals G, Mulder BJM, Groenink M (2014). Diagnosis and genetics of Marfan syndrome.. Expert Opin Orphan Drugs.

[2] Ramirez F, Sakai LY (2010). Biogenesis and function of fibrillin assemblies.. Cell Tissue Res.

[3] Jondeau G, Michel JB, Boileau C (2011). The translational science of Marfan syndrome.. Heart.

[4] Mimoun L, Detaint D, Hamroun D, Arnoult F, Delorme G, Gautier M, Milleron O, Meuleman C, Raoux F, Boileau C (2011). Dissection in Marfan syndrome: the importance of the descending aorta.. Eur Heart J.

[5] den Hartog AW, Franken R, Zwinderman AH, Timmermans J, Scholte AJ, van den Berg MP, de Waard V, Pals G, Mulder BJ, Groenink M (2015). The risk for type B aortic dissection in Marfan syndrome.. J Am Coll Cardiol.

[6] Milleron O, Arnoult F, Ropers J, Aegerter P, Detaint D, Delorme G, Attias D, Tubach F, Dupuis-Girod S, Plauchu H (2015). Marfan Sartan: a randomized, double-blind, placebo-controlled trial.. Eur Heart J.

[7] Lacro RV, Dietz HC, Sleeper LA, Yetman AT, Bradley TJ, Colan SD, Pearson GD, Selamet Tierney ES, Levine JC, Atz AM, Pediatric Heart Network Investigators (2014). Atenolol versus losartan in children and young adults with Marfan’s syndrome.. N Engl J Med.

[8] Forteza A, Evangelista A, Sánchez V, Teixidó-Turà G, Sanz P, Gutiérrez L, Gracia T, Centeno J, Rodríguez-Palomares J, Rufilanchas JJ (2016). Efficacy of losartan vs. atenolol for the prevention of aortic dilation in Marfan syndrome: a randomized clinical trial.. Eur Heart J.

[9] Sakai LY, Keene DR, Renard M, De Backer J (2016). FBN1: the disease-causing gene for Marfan syndrome and other genetic disorders.. Gene.

[10] Halme T, Savunen T, Aho H, Vihersaari T, Penttinen R (1985). Elastin and collagen in the aortic wall: changes in the Marfan syndrome and annuloaortic ectasia.. Exp Mol Pathol.

[11] Perrucci GL, Rurali E, Gowran A, Pini A, Antona C, Chiesa R, Pompilio G, Nigro P (2017). Vascular smooth muscle cells in Marfan syndrome aneurysm: the broken bricks in the aortic wall.. Cell Mol Life Sci.

[12] Lee JJ, Galatioto J, Rao S, Ramirez F, Costa KD (2016). Losartan attenuates degradation of aorta and lung tissue micromechanics in a mouse model of severe Marfan syndrome.. Ann Biomed Eng.

[13] Lindeman JH, Ashcroft BA, Beenakker JW, van Es M, Koekkoek NB, Prins FA, Tielemans JF, Abdul-Hussien H, Bank RA, Oosterkamp TH (2010). Distinct defects in collagen microarchitecture underlie vessel-wall failure in advanced abdominal aneurysms and aneurysms in Marfan syndrome.. Proc Natl Acad Sci USA.

[14] Lönnqvist L, Karttunen L, Rantamäki T, Kielty C, Raghunath M, Peltonen L (1996). A point mutation creating an extra N-glycosylation site in fibrillin-1 results in neonatal Marfan syndrome.. Genomics.

[15] Lynch M, Barallobre-Barreiro J, Jahangiri M, Mayr M (2016). Vascular proteomics in metabolic and cardiovascular diseases.. J Intern Med.

[16] Saba J, Dutta S, Hemenway E, Viner R (2012). Increasing the productivity of glycopeptides analysis by using higher-energy collision dissociation-accurate mass-product-dependent electron transfer dissociation.. Int J Proteomics.

[17] Yin X, Bern M, Xing Q, Ho J, Viner R, Mayr M (2013). Glycoproteomic analysis of the secretome of human endothelial cells.. Mol Cell Proteomics.

[18] Didangelos A, Yin X, Mandal K, Baumert M, Jahangiri M, Mayr M (2010). Proteomics characterization of extracellular space components in the human aorta.. Mol Cell Proteomics.

[19] Barallobre-Barreiro J, Baig F, Fava M, Yin X, Mayr M (2017). Glycoproteomics of the extracellular matrix: A method for intact glycopeptide analysis using mass spectrometry.. J Vis Exp.

[20] Radonic T, de Witte P, Baars MJ, Zwinderman AH, Mulder BJ, Groenink M, COMPARE study group (2010). Losartan therapy in adults with Marfan syndrome: study protocol of the multi-center randomized controlled COMPARE trial.. Trials.

[21] Franken R, den Hartog AW, de Waard V, Engele L, Radonic T, Lutter R, Timmermans J, Scholte AJ, van den Berg MP, Zwinderman AH (2013). Circulating transforming growth factor-β as a prognostic biomarker in Marfan syndrome.. Int J Cardiol.

[22] Groenink M, den Hartog AW, Franken R, Radonic T, de Waard V, Timmermans J, Scholte AJ, van den Berg MP, Spijkerboer AM, Marquering HA (2013). Losartan reduces aortic dilatation rate in adults with Marfan syndrome: a randomized controlled trial.. Eur Heart J.

[23] De Paepe A, Devereux RB, Dietz HC, Hennekam RC, Pyeritz RE (1996). Revised diagnostic criteria for the Marfan syndrome.. Am J Med Genet.

[24] Fava M, Barallobre-Barreiro J, Mayr U, Lu R, Didangelos A, Baig F, Lynch M, Catibog N, Joshi A, Barwari T (2018). Role of ADAMTS-5 in aortic dilatation and extracellular matrix remodeling.. Arterioscler Thromb Vasc Biol.

[25] Geisterfer AA, Peach MJ, Owens GK (1988). Angiotensin II induces hypertrophy, not hyperplasia, of cultured rat aortic smooth muscle cells.. Circ Res.

[26] Granata A, Serrano F, Bernard WG, McNamara M, Low L, Sastry P, Sinha S (2017). An iPSC-derived vascular model of Marfan syndrome identifies key mediators of smooth muscle cell death.. Nat Genet.

[27] Cheung C, Bernardo AS, Trotter MW, Pedersen RA, Sinha S (2012). Generation of human vascular smooth muscle subtypes provides insight into embryological origin-dependent disease susceptibility.. Nat Biotechnol.

[28] Serrano F, Bernard WG, Granata A, Iyer D, Steventon B, Kim M, Vallier L, Gambardella L, Sinha S (2019). A novel human pluripotent stem cell-derived neural crest model of treacher collins syndrome shows defects in cell death and migration.. Stem Cells Dev.

[29] Zampetaki A, Kiechl S, Drozdov I, Willeit P, Mayr U, Prokopi M, Mayr A, Weger S, Oberhollenzer F, Bonora E (2010). Plasma microRNA profiling reveals loss of endothelial miR-126 and other microRNAs in type 2 diabetes.. Circ Res.

[30] Groenink M, de Roos A, Mulder BJ, Spaan JA, van der Wall EE (1998). Changes in aortic distensibility and pulse wave velocity assessed with magnetic resonance imaging following beta-blocker therapy in the Marfan syndrome.. Am J Cardiol.

[31] Nistri S, Sorbo MD, Marin M, Palisi M, Scognamiglio R, Thiene G (1999). Aortic root dilatation in young men with normally functioning bicuspid aortic valves.. Heart.

[32] Nathan DP, Xu C, Plappert T, Desjardins B, Gorman JH, Bavaria JE, Gorman RC, Chandran KB, Jackson BM (2011). Increased ascending aortic wall stress in patients with bicuspid aortic valves.. Ann Thorac Surg.

[33] Sandy JD, Westling J, Kenagy RD, Iruela-Arispe ML, Verscharen C, Rodriguez-Mazaneque JC, Zimmermann DR, Lemire JM, Fischer JW, Wight TN (2001). Versican V1 proteolysis in human aorta *in vivo* occurs at the Glu441-Ala442 bond, a site that is cleaved by recombinant ADAMTS-1 and ADAMTS-4.. J Biol Chem.

[34] Suna G, Wojakowski W, Lynch M, Barallobre-Barreiro J, Yin X, Mayr U, Baig F, Lu R, Fava M, Hayward R (2018). Extracellular matrix proteomics reveals interplay of aggrecan and aggrecanases in vascular remodeling of stented coronary arteries.. Circulation.

[35] Oller J, Méndez-Barbero N, Ruiz EJ, Villahoz S, Renard M, Canelas LI, Briones AM, Alberca R, Lozano-Vidal N, Hurlé MA (2017). Nitric oxide mediates aortic disease in mice deficient in the metalloprotease Adamts1 and in a mouse model of Marfan syndrome.. Nat Med.

[36] Didangelos A, Yin X, Mandal K, Saje A, Smith A, Xu Q, Jahangiri M, Mayr M (2011). Extracellular matrix composition and remodeling in human abdominal aortic aneurysms: a proteomics approach.. Mol Cell Proteomics.

[37] Rienks M, Papageorgiou AP, Frangogiannis NG, Heymans S (2014). Myocardial extracellular matrix: an ever-changing and diverse entity.. Circ Res.

[38] Vogt G, Vogt B, Chuzhanova N, Julenius K, Cooper DN, Casanova JL (2007). Gain-of-glycosylation mutations.. Curr Opin Genet Dev.

[39] Lausen M, Lynch N, Schlosser A, Tornoe I, Saekmose SG, Teisner B, Willis AC, Crouch E, Schwaeble W, Holmskov U (1999). Microfibril-associated protein 4 is present in lung washings and binds to the collagen region of lung surfactant protein D.. J Biol Chem.

[40] Mecham RP, Gibson MA (2015). The microfibril-associated glycoproteins (MAGPs) and the microfibrillar niche.. Matrix Biol.

[41] Rachmilewitz J (2010). Glycosylation: an intrinsic sign of “danger”.. Self Nonself.

[42] Chiarini A, Onorati F, Marconi M, Pasquali A, Patuzzo C, Malashicheva A, Irtyega O, Faggian G, Pignatti PF, Trabetti E (2018). Studies on sporadic non-syndromic thoracic aortic aneurysms: II. Alterations of extra-cellular matrix components and focal adhesion proteins.. Eur J Prev Cardiol.

[43] Chung AW, Au Yeung K, Sandor GG, Judge DP, Dietz HC, van Breemen C (2007). Loss of elastic fiber integrity and reduction of vascular smooth muscle contraction resulting from the upregulated activities of matrix metalloproteinase-2 and -9 in the thoracic aortic aneurysm in Marfan syndrome.. Circ Res.

[44] Kielty CM, Sherratt MJ, Shuttleworth CA (2002). Elastic fibres.. J Cell Sci.

[45] Pilecki B, Holm AT, Schlosser A, Moeller JB, Wohl AP, Zuk AV, Heumüller SE, Wallis R, Moestrup SK, Sengle G (2016). Characterization of microfibrillar-associated protein 4 (MFAP4) as a tropoelastin- and fibrillin-binding protein involved in elastic fiber formation.. J Biol Chem.

[46] Pilop C, Aregger F, Gorman RC, Brunisholz R, Gerrits B, Schaffner T, Gorman JH, Matyas G, Carrel T, Frey BM (2009). Proteomic analysis in aortic media of patients with Marfan syndrome reveals increased activity of calpain 2 in aortic aneurysms.. Circulation.

[47] Schlosser A, Pilecki B, Hemstra LE, Kejling K, Kristmannsdottir GB, Wulf-Johansson H, Moeller JB, Füchtbauer EM, Nielsen O, Kirketerp-Møller K (2016). MFAP4 promotes vascular smooth muscle migration, proliferation and accelerates neointima formation.. Arterioscler Thromb Vasc Biol.

[48] Holm TM, Habashi JP, Doyle JJ, Bedja D, Chen Y, van Erp C, Lindsay ME, Kim D, Schoenhoff F, Cohn RD (2011). Noncanonical TGFβ signaling contributes to aortic aneurysm progression in Marfan syndrome mice.. Science.

[49] Gomez D, Al Haj Zen A, Borges LF, Philippe M, Gutierrez PS, Jondeau G, Michel JB, Vranckx R (2009). Syndromic and non-syndromic aneurysms of the human ascending aorta share activation of the Smad2 pathway.. J Pathol.

[50] Kim KL, Yang JH, Song SH, Kim JY, Jang SY, Kim JM, Kim JA, Sung KI, Kim YW, Suh YL (2013). Positive correlation between the dysregulation of transforming growth factor-β1 and aneurysmal pathological changes in patients with Marfan syndrome.. Circ J.

[51] Nataatmadja M, West J, Prabowo S, West M (2013). Angiotensin II receptor antagonism reduces transforming growth factor beta and smad signaling in thoracic aortic aneurysm.. Ochsner J.

[52] Cikach FS, Koch CD, Mead TJ, Galatioto J, Willard BB, Emerton KB, Eagleton MJ, Blackstone EH, Ramirez F, Roselli EE (2018). Massive aggrecan and versican accumulation in thoracic aortic aneurysm and dissection.. JCI Insight.

[53] Hubmacher D, Apte SS (2015). ADAMTS proteins as modulators of microfibril formation and function.. Matrix Biol.

[54] Aviram R, Zaffryar-Eilot S, Hubmacher D, Grunwald H, Mäki JM, Myllyharju J, Apte SS, Hasson P (2019). Interactions between lysyl oxidases and ADAMTS proteins suggest a novel crosstalk between two extracellular matrix families.. Matrix Biol.

[55] Rienks M, Barallobre-Barreiro J, Mayr M (2018). The emerging role of the ADAMTS family in vascular diseases.. Circ Res.

