# Supplementary figures and images for: Glycoproteomic Analysis of the Aortic Extracellular Matrix in Marfan Patients
Source: Arterioscler Thromb Vasc Biol. 2019 Jun 13;39(9):1859–73. doi: 10.1161/ATVBAHA.118.312175 (PMC6727943; doi:10.1161/ATVBAHA.118.312175)

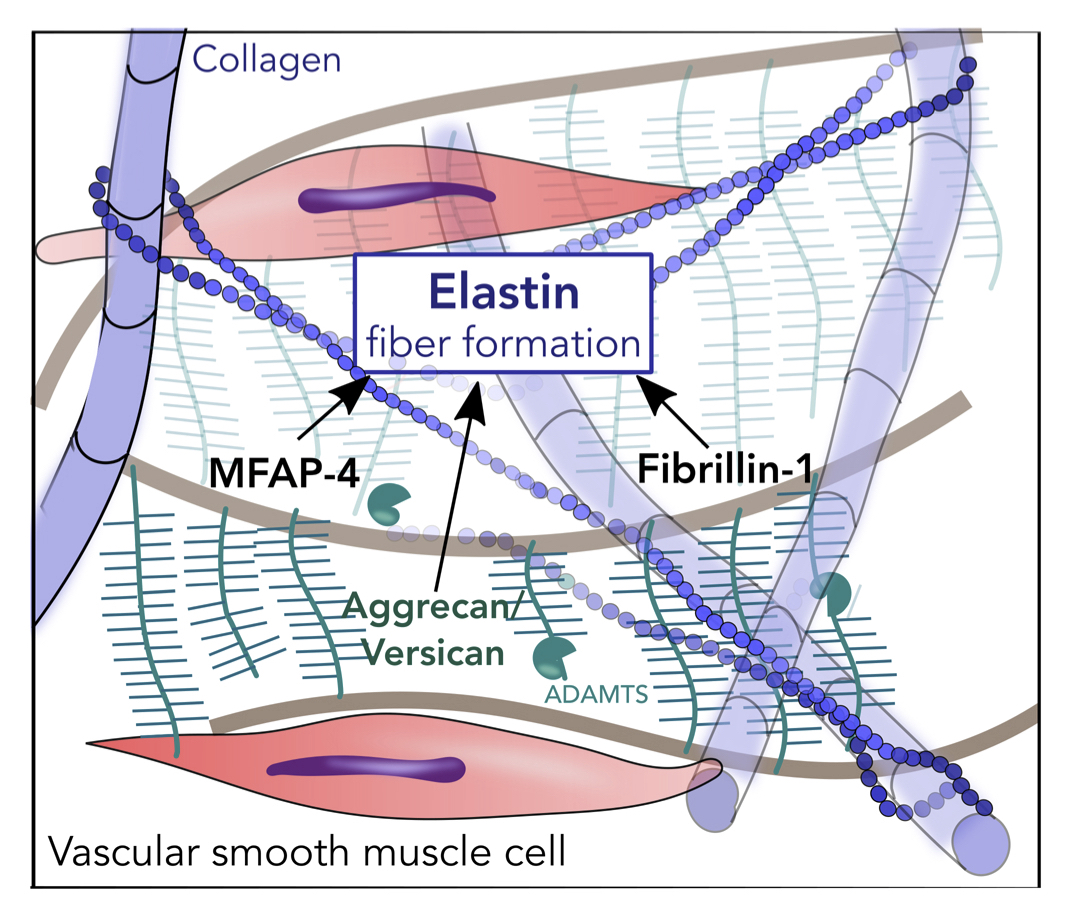

Supplement: Supplementary file 1 [file atv-39-1859-s001.jpg]
