# Supplementary material for: Glycoproteomic Analysis of the Aortic Extracellular Matrix in Marfan Patients
Source: Arterioscler Thromb Vasc Biol. 2019 Jun 13;39(9):1859–73. doi: 10.1161/ATVBAHA.118.312175 (PMC6727943; doi:10.1161/ATVBAHA.118.312175)
Supplement: Supplementary file 2 [file atv-39-1859-s002.pdf]

Major Resources Tables

Primary Antibodies

Target antigen	Vendor or Source	Catalog #	Working concentration
Aggrecan neo-epitope	Thermo Fisher Scientific	PA1-1746	1µg/mL
Versican neo-epitope	Abcam	ab19345	1µg/mL
MFAP4 N-terminal	Abcam	ab169757	1:1000
MFAP4 N-terminal	Abcam	ab80319	1µg/mL
TGF-β1	Abcam	ab179695	0.5µg/mL

Secondary Antibody

Vendor or Source	Target Species	Catalog #	Working concentration
Jackson ImmunoResearch Europe Ltd	Rabbit	211-032-171	80ng/mL

Cultured Cells

Name	Vendor or Source	Sex (F, M, or unknown)
Human aortic smooth muscle cells	PromoCell, C-12533, donor 1	unknown
Human aortic smooth muscle cells	PromoCell, C-12533, donor 2	unknown
Human aortic smooth muscle cells	PromoCell, C-12533, donor 3	unknown
Fibroblast from Skin of MFS patient	Coriell Institute, GM21943	Male

Enzymes

Name	Vendor or Source	Catalog Number
Endo-α-N-acetylgalactosaminidase	Merk-Millipore Glycoprotein Deglycosylation Kit	362280
β1,4-galactosidase		
β-N-acetylglucosaminidase		
α2-3,6,8,9-neuraminidase		
N-Glycosidase F		
Chondroitinase ABC	Sigma-Aldrich	C3667
Heparinase II	Sigma-Aldrich	H6512
Endo-β1,4-galactosidase	Sigma-Aldrich	G6920
Trypsin	Thermo Fisher Scientific	90057

Taqman Assay Probes

Human Gene Name	Assay ID
ACAN	Hs00153936_m1
ADAMTS1	Hs00199608_m1
ADAMTS4	Hs00192708_m1
ADAMTS5	Hs00199841_m1
ADAMTS7	Hs00276223_m1
AEBP1	Hs00937468_m1
BGN	Hs00959141_g1
CLU	Hs00971656_m1
COL12A1	Hs00189184_m1
COL4A1	Hs00266237_m1
COL6A3	Hs00915125_m1
CTGF	Hs01026927_g1
CTSA	Hs01563955_m1
CTSG	Hs01113415_g1
CTSK	Hs00166156_m1
CTSL	Hs00964650_m1
CTSS	Hs01080395_m1
DCN	Hs00754870_s1
EFEMP1	Hs01013942_m1
ELN	Hs00355783_m1
FBLN1	Hs00972609_m1
FBLN5	Hs00197064_m1
FBN1	Hs00171191_m1
FMOD	Hs00157619_m1
GAPDH *	Hs99999905-m1
GSN	Hs00609272_m1
HTRA1	Hs01016151_m1
ITGA1	Hs00235006_m1
ITGB1	Hs00559595_m1
LRP1	Hs00233856_m1
LTBP1	Hs00386448_m1
LTBP2	Hs00166367_m1
LTBP4	Hs00186025_m1
LUM	Hs00929860_m1
MFAP4	Hs00412974_m1
MFGE8	Hs00170712_m1
MGP	Hs00179899_m1
MMP14	Hs01037009_g1

MMP2	Hs01548727_m1
MMP9	Hs00957562_m1
NID1	Hs00159600_m1
OGN	Hs00247901_m1
POSTN	Hs01566734_m1
PRELP	Hs01941580_s1
TGFB1	Hs00998133_m1
TGFB2	Hs00234244_m1
TGFB3	Hs01086000_m1
TGFBR1	Hs00610320_m1
TGFBR2	Hs00234253_m1
TIMP1	Hs00355335_g1
TIMP2	Hs00234278_m1
TIMP3	Hs00927214_m1
TNC	Hs01115665_m1
TPSB1	Hs02576518_gH
VCAN	Hs00171642_m1
ZYX	Hs00170299_m1
18s *	Hs99999901_s1

* *GAPDH* and *18s* were used for normalization.

Primers for qPCR using SYBR green

**Mouse Gene**	**Forward primer (5'to 3')**	**Reverse primer (5'to 3')**
Mfap4	CTCCACGCTTTACCCACAAT	AGCTGTCGTGAAGGGGTAGA
Rplp0 *	GGACCCGAGAAGACCTCCTT	GCACATCACTCAGAATTTCAATGG

* *Rplp0* was used for normalization.

Si-RNA for *MFAP4* knockdown in HAoSMC

**Name**	**Vendor**	**Catalog Number**	**Assay ID**
Silencer Negative Control No. 2 siRNA	Life Technologies	AM4613	-
Silencer siRNA targeting MFAP4	Life Technologies	AM16708	11386
Silencer Select siRNA targeting MFAP4	Life Technologies	4392420	s8716
