# Supplementary material for: Glycoproteomic Analysis of the Aortic Extracellular Matrix in Marfan Patients
Source: Arterioscler Thromb Vasc Biol. 2019 Jun 13;39(9):1859–73. doi: 10.1161/ATVBAHA.118.312175 (PMC6727943; doi:10.1161/ATVBAHA.118.312175)
Supplement: Supplementary file 3 [file atv-39-1859-s003.pdf]

SUPPLEMENTAL MATERIAL

GLYCOPROTEOMIC ANALYSIS OF THE AORTIC EXTRACELLULAR MATRIX IN MARFAN PATIENTS

Xiaoke Yin*[1], Shaynah Wanga*[2,3], Adam Fellows[1], Javier Barallobre-Barreiro[1], Ruifang Lu[1], Hongorzul Davaapil[4], Romy Franken[3], Marika Fava[1], Ferheen Baig[1], Philipp Skroblin[1], Qiuru Xing[1], David R. Koolbergen[5], Maarten Groenink[3,6], Aeilko H. Zwinderman[7], Ron Balm[8], Carlie J.M. de Vries[2], Barbara J.M. Mulder[3,9], Rosa Viner[10], Marjan Jahangiri[11], Dieter P. Reinhardt[12], Sanjay Sinha[4], Vivian de Waard*[2], Manuel Mayr*[1]

* These authors contributed equally as shared first and shared senior authors.

[1] King's British Heart Foundation Centre, King's College London, London, UK.

[2] Department of Medical Biochemistry, Amsterdam Cardiovascular Sciences, Amsterdam UMC, University of Amsterdam, Amsterdam, The Netherlands.

[3] Department of Cardiology, Amsterdam UMC, University of Amsterdam, Amsterdam, The Netherlands.

[4] Wellcome-MRC Cambridge Stem Cell Institute & Department of Medicine, University of Cambridge, Cambridge, UK

[5] Department of Cardiothoracic Surgery, Amsterdam UMC, University of Amsterdam, Amsterdam, The Netherlands.

[6] Department of Radiology, Amsterdam UMC, University of Amsterdam, Amsterdam, The Netherlands.

[7] Department of Clinical Epidemiology, Biostatistics & Bioinformatics, Amsterdam UMC, University of Amsterdam, Amsterdam, The Netherlands.

[8] Department of Surgery, Amsterdam UMC, University of Amsterdam, Amsterdam, The Netherlands.

[9] Netherlands Heart Institute, Utrecht, The Netherlands.

[10] Thermo Fisher Scientific, San Jose, USA.

[11] St George's, University of London, London, UK

[12] Faculty of Medicine, Department of Anatomy and Cell Biology and Faculty of Dentistry, McGill University, Montreal, Canada

ADDRESS FOR CORRESPONDENCE:

Prof. Manuel Mayr, MD, PhD, King's British Heart Foundation Centre, King's College London, 125 Coldharbour Lane, London, SE5 9NU, UK. Telephone: +44 20 7848 5446, Fax: 020 7848 5489, Email: manuel.mayr@kcl.ac.uk

Supplemental Figure I

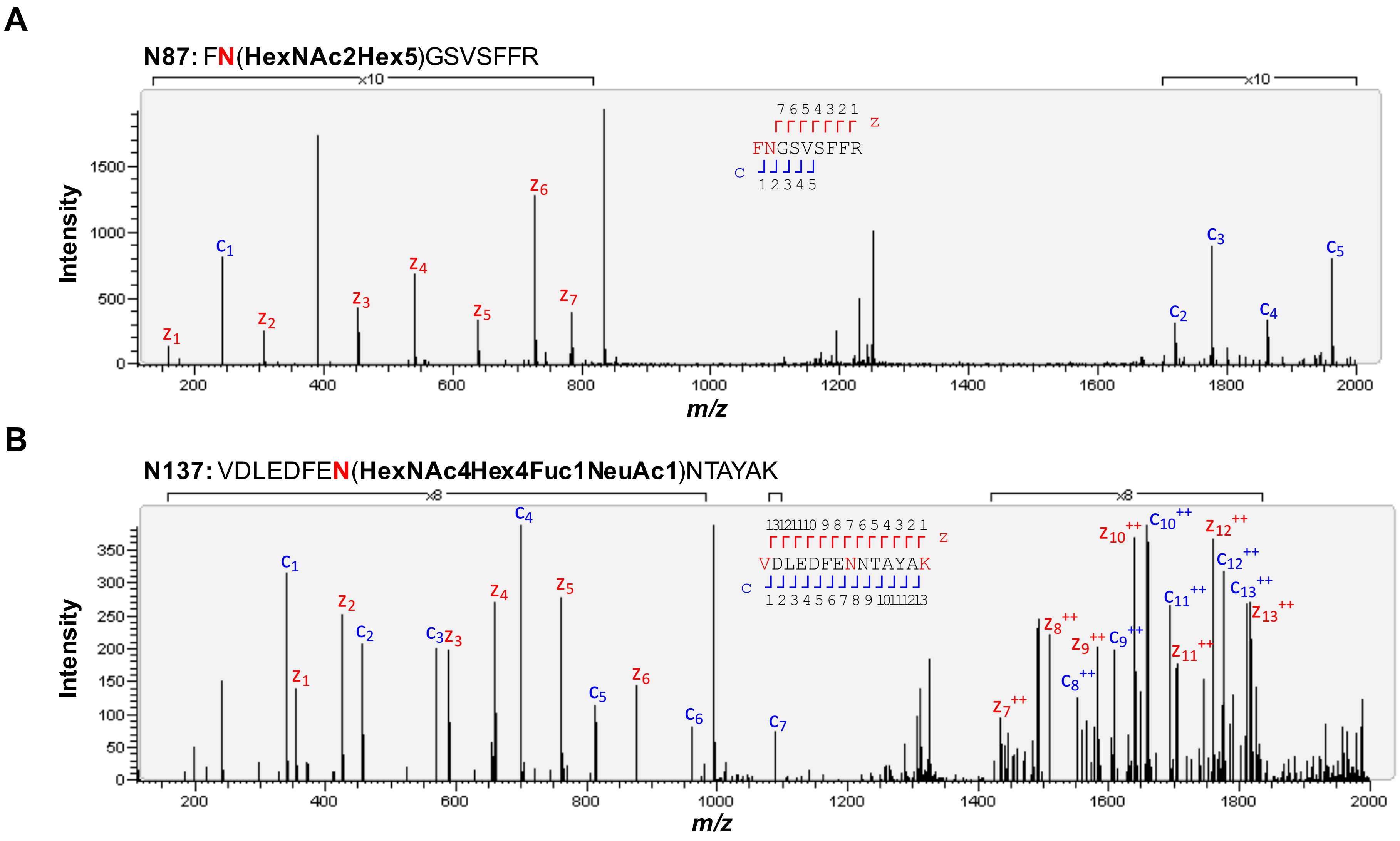

Supplemental Figure I. Representative MS/MS spectra for the identified peptide sequences and glycoforms of MFAP4 at glycosylation site N87 **(A)** and N137 **(B)**. Electron transfer dissociation (ETD)was used to preserve the glycosylation while fragmenting peptides. c ions and z ions were used to calculate the peptide sequences and the glycan compositions were calculated based on the mass added to the peptides by Byonic software. Low signal area were zoomed 10x or 8x as indicated on the top of each spectra.

Supplemental Figure II

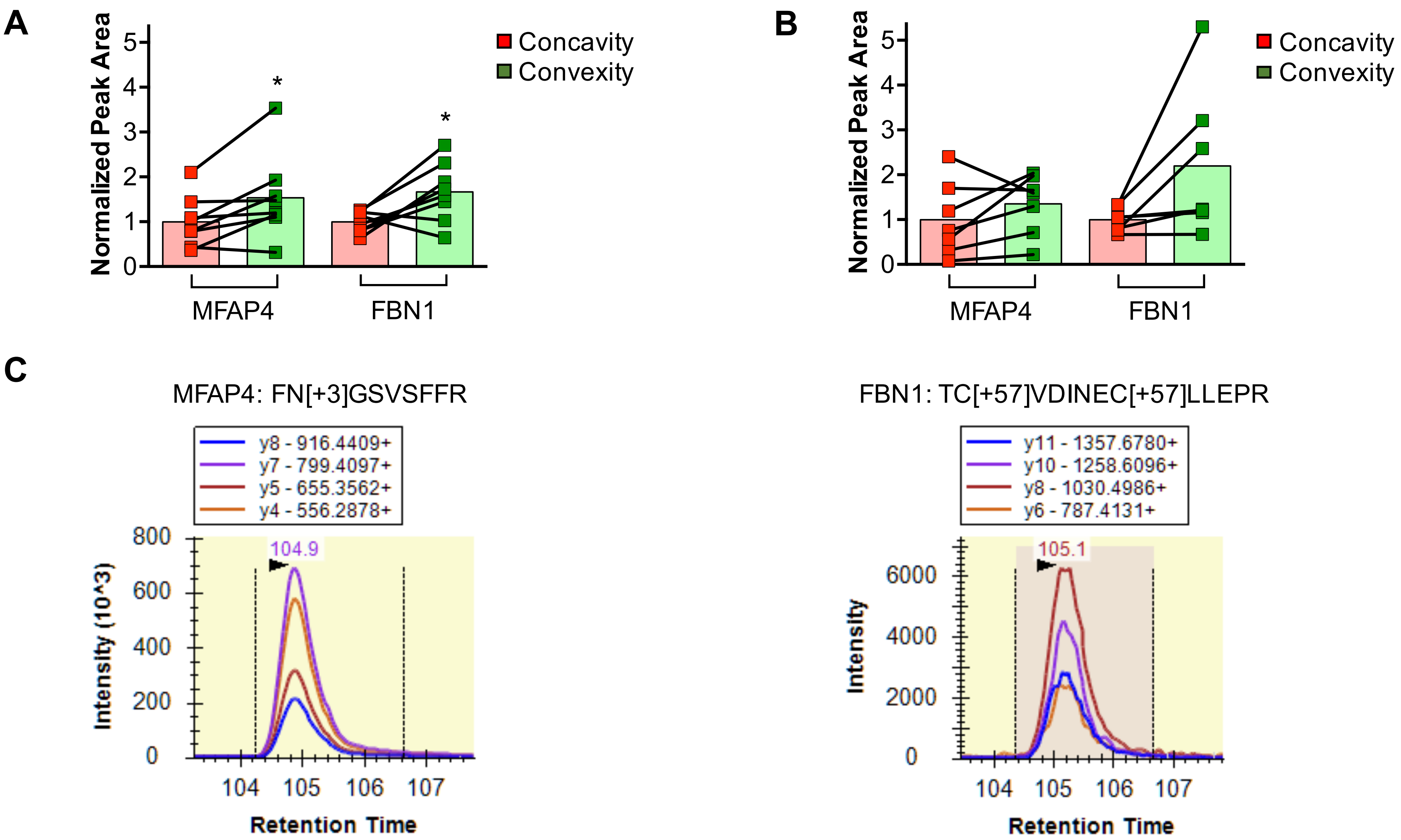

Supplemental Figure II. The tissues from the concave area and convex area of the same aorta from non-aneurysmal BAV patients (n=8) were enriched for ECM proteins, deglycosylated, and digested. The selected peptides were analysed using scheduled MRM method on nanoflow LC-MS/MS (U3000 RSLCnano coupled with TSQ Vantage triple quadrupole MS, Thermo Fisher Scientific). The MRM results confirmed the label-free quantitation results using data dependant analysis **(A)**. The same method was applied to non-aneurysmal TAV patients (n=7) and there was no significant changes of MFAP4 between concave area and convex area **(B)**. Representative peptide and transition peaks were shown in **(C)**. * p<0.05 using paired Student t-Test. [+3] is O18-deglycosylation, [+57] is carbamidomethylation.

Supplemental Figure III

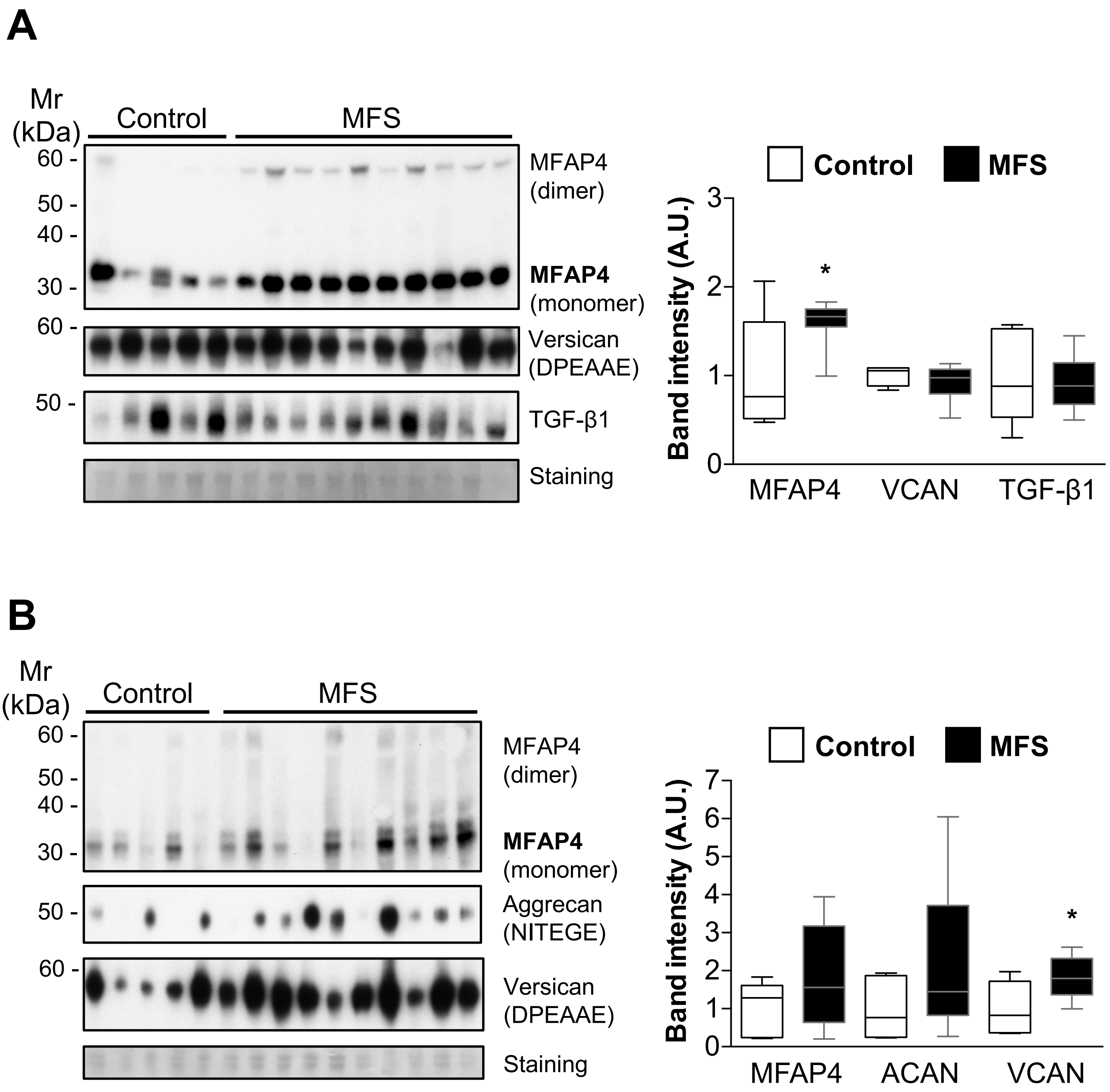

Supplemental Figure III. Immunoblotting and the densitometry analysis of NaCl **(A)** and GuHCl **(B)** extracts of human aorta tissue samples. Bands intensities were measured using Image Quant TL (version 8.1, GE healthcare) and Student's *t*-Test was performed to determine the significance between the control group and MFS group. * P<0.05.

Supplemental Figure IV

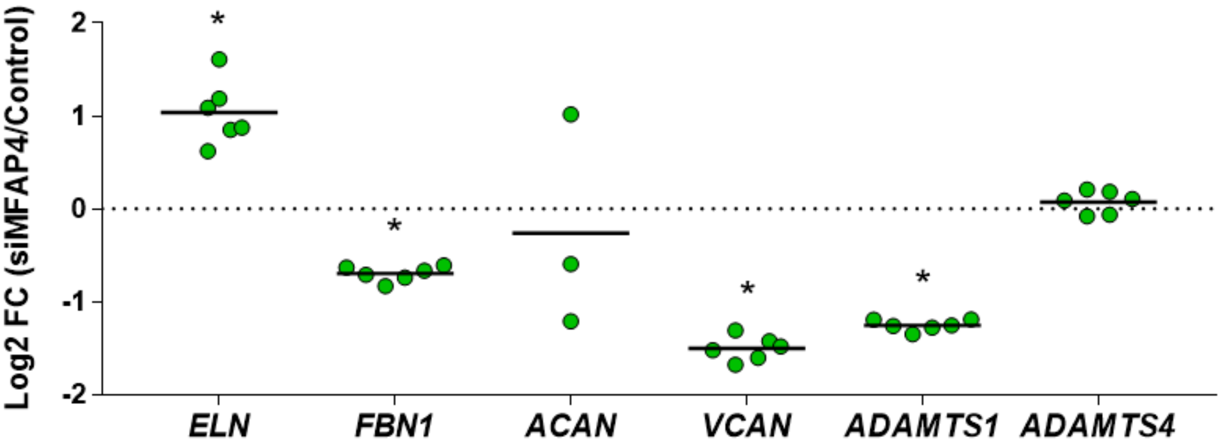

Supplemental Figure IV. Silencing of MFAP4 by a different si-RNA targeting MFAP4 in human aortic SMCs confirmed the mRNA changes as shown in Figure 5C. Gene expression was normalised to control si-RNA group (dotted line). Non-parametric paired test was performed and adjusted *P* value was calculated. *P<0.05.

Supplemental Table I. Patient characteristics for samples used for glycoproteomics.

Group	Sample	Gender	Age	Disorder
Control	ascending aorta	Male	52	Familial thoracic aortic aneurysm and dissection
Control	ascending aorta	Female	18	Loeys-Dietz syndrome
Control	ascending aorta	Female	31	Connective tissue disorder
Control	ascending aorta	Male	73	Thoracic aortic aneurysm disease
Control	ascending aorta	Female	81	Thoracic aortic aneurysm disease
Control	ascending aorta	Female	82	Thoracic aortic aneurysm disease
MFS	ascending aorta	Female	21	MFS
MFS	ascending aorta	Female	56	MFS
MFS	ascending aorta	Male	35	MFS
MFS	ascending aorta	Male	39	MFS
MFS	ascending aorta	Female	48	MFS
MFS	ascending aorta	Female	24	MFS
MFS	ascending aorta	Female	47	MFS
MFS	ascending aorta	Male	54	MFS
MFS	ascending aorta	Female	48	MFS
MFS	ascending aorta	Male	25	MFS
MFS	ascending aorta	Male	28	MFS

Supplemental Table II. BAV and TAV patients characteristics.

	BAV patients (n=8)	TAV patients (n=7)
Male (%)	37.5	57.1
Age (years)	63 ± 4.4	65.6 ± 15.3
Ascending aorta dilatation (cm)	3.2 ± 0.2	3.7 ± 0.2
Stenosis (%)	100	42.9
Regurgitation (%)	37.5	57.1

Supplemental Table III. Glycopeptides identified by LC-MS/MS

Protein Name	UniProt Entry Name	Sequence	Positions	Modifications	Glycan composition	Glycosite	# PSMs	Theo. MH+ [Da]	# Missed Cleavages	Off By X	|Log Prob|	Byonic Score	Delta Byonic Score	Delta Mod Score	PEP 2D	q-Value 2D	FDR 2D	Peptide Group FDR 2D	PEP 1D	q-Value 1D	FDR 1D	Peptide Group FDR 1D
Aggrecan core protein	PGCA_HUMAN	SNDSGVYR	125-132	TMT0 [N-Term]; HexNAc(2)Hex(5) [N2]	HexNAc(2)Hex(5)	126	5	2337.9814	0	0	7.18	383.9	383.9	383.9	6.53E-08	4.36E-09	0	0	2.56E-07	1.70E-08	0	0
Aggrecan core protein	PGCA_HUMAN	SNDSGVYR	125-132	TMT0 [N-Term]; HexNAc(2)Hex(6) [N2]	HexNAc(2)Hex(6)	126	5	2500.0343	0	0	5.63	361.0	361.0	361.0	2.35E-06	1.73E-07	0	0	9.21E-06	6.79E-07	0	0
Aggrecan core protein	PGCA_HUMAN	TVYVHANQTGYPDPSSR	327-343	TMT0 [N-Term]; HexNAc(2)Hex(12) [N7]	HexNAc(2)Hex(12)	333	1	4466.8384	0	0	6.54	472.2	430.8	3.8	2.90E-07	1.87E-08	0	0	1.14E-06	7.39E-08	0	0
Aggrecan core protein	PGCA_HUMAN	TVYVHANQTGYPDPSSR	327-343	TMT0 [N-Term]; HexNAc(4)Hex(5)Fuc(1)NeuAc(1) [N7]	HexNAc(4)Hex(5)Fuc(1)NeuAc(1)	333	1	4175.7807	0	0	8.48	466.4	394.3	18.0	3.32E-09	3.63E-10	0	0	1.30E-08	1.42E-09	0	0
Aggrecan core protein	PGCA_HUMAN	TVYVHANQTGYPDPSSR	327-343	TMT0 [N-Term]; HexNAc(4)Hex(5)Fuc(1)NeuAc(2) [N7]	HexNAc(4)Hex(5)Fuc(1)NeuAc(2)	333	2	4466.8761	0	0	6.57	392.8	360.4	4.0	2.70E-07	1.75E-08	0	0	1.06E-06	6.90E-08	0	0
Alpha-2-HS-glycoprotein	FETUA_HUMAN	VCQDCPLLAPLNDTR	145-159	TMT0 [N-Term]; Carbamidomethyl [C2]; Carbamidomethyl [C5]; HexNAc(4)Hex(5)NeuAc(2) [N12]	HexNAc(4)Hex(5)NeuAc(2)	156	11	4200.7715	0	0	6.37	361.8	348.0	137.2	4.22E-07	2.85E-08	0	0	1.66E-06	1.13E-07	0	0
Alpha-2-HS-glycoprotein	FETUA_HUMAN	AALAAFNAQNNGSNFQLEEISR	166-187	TMT0 [N-Term]; HexNAc(4)Hex(5)NeuAc(2) [N11]	HexNAc(4)Hex(5)NeuAc(2)	176	1	4794.0780	0	-1	6.03	366.5	354.6	354.6	9.24E-07	6.78E-08	0	0	3.62E-06	2.67E-07	0	0
Basement membrane-specific heparan sulfate proteoglycan core protein	PGBM_HUMAN	ALVNFTR	86-92	TMT0 [N-Term]; HexNAc(3)Hex(4)NeuAc(1) [N4]	HexNAc(3)Hex(4)NeuAc(1)	89	2	2593.1649	0	0	4.86	283.0	283.0	283.0	1.38E-05	8.27E-07	0	0	5.42E-05	3.08E-05	2.97E-05	3.67E-04
Basement membrane-specific heparan sulfate proteoglycan core protein	PGBM_HUMAN	ALVNFTR	86-92	TMT0 [N-Term]; HexNAc(4)Hex(5)NeuAc(1) [N4]	HexNAc(4)Hex(5)NeuAc(1)	89	18	2958.2971	0	0	5.50	368.2	334.4	334.4	3.17E-06	2.30E-07	0	0	1.24E-05	9.01E-07	0	0
Basement membrane-specific heparan sulfate proteoglycan core protein	PGBM_HUMAN	ALVNFTR	86-92	TMT0 [N-Term]; HexNAc(4)Hex(5)NeuAc(2) [N4]	HexNAc(4)Hex(5)NeuAc(2)	89	32	3249.3925	0	0	5.03	369.9	266.4	266.4	9.43E-06	5.96E-07	0	0	3.70E-05	3.08E-05	3.03E-05	3.73E-04
Basement membrane-specific heparan sulfate proteoglycan core protein	PGBM_HUMAN	ALVNFTR	86-92	TMT0 [N-Term]; HexNAc(5)Hex(6)NeuAc(2) [N4]	HexNAc(5)Hex(6)NeuAc(2)	89	4	3614.5247	0	0	3.89	228.3	225.6	225.6	1.29E-04	2.83E-05	2.64E-05	3.31E-04	5.04E-04	3.08E-05	2.67E-05	3.31E-04
Basement membrane-specific heparan sulfate proteoglycan core protein	PGBM_HUMAN	ALVNFTR	86-92	TMT0 [N-Term]; HexNAc(5)Hex(6)NeuAc(3) [N4]	HexNAc(5)Hex(6)NeuAc(3)	89	3	3905.6201	0	0	3.74	261.3	228.5	228.5	1.82E-04	5.26E-05	5.21E-05	3.26E-04	7.15E-04	5.33E-05	5.26E-05	3.26E-04
Basement membrane-specific heparan sulfate proteoglycan core protein	PGBM_HUMAN	NLHQSNTSR	1750-1758	TMT0 [N-Term]; HexNAc(2)Hex(5) [N6]	HexNAc(2)Hex(5)	1755	3	2497.0934	0	0	5.26	270.2	270.2	270.2	5.54E-06	3.80E-07	0	0	2.17E-05	1.49E-06	0	0
Basement membrane-specific heparan sulfate proteoglycan core protein	PGBM_HUMAN	NLHQSNTSR	1750-1758	TMT0 [N-Term]; HexNAc(4)Hex(5)NeuAc(1) [N6]	HexNAc(4)Hex(5)NeuAc(1)	1755	1	3194.3476	0	0	2.04	159.0	159.0	159.0	9.18E-03	5.45E-04	5.36E-04	5.83E-04	2.48E-02	2.59E-04	2.59E-04	2.33E-03
Basement membrane-specific heparan sulfate proteoglycan core protein	PGBM_HUMAN	SLTQGSLIVGDLAPVNGTSQGK	3765-3786	TMT0 [N-Term]; HexNAc(3)Hex(5)Fuc(1)NeuAc(1) [N16]	HexNAc(3)Hex(5)Fuc(1)NeuAc(1)	3780	1	4222.9481	0	0	5.02	328.1	297.7	83.8	9.52E-06	5.99E-07	0	0	3.73E-05	3.08E-05	3.03E-05	3.73E-04
Basement membrane-specific heparan sulfate proteoglycan core protein	PGBM_HUMAN	SLTQGSLIVGDLAPVNGTSQGK	3765-3786	TMT0 [N-Term]; HexNAc(4)Hex(5)Fuc(1)NeuAc(1) [N16]	HexNAc(4)Hex(5)Fuc(1)NeuAc(1)	3780	5	4426.0275	0	0	6.80	432.9	349.4	103.4	1.57E-07	9.80E-09	0	0	6.16E-07	3.85E-08	0	0
Beta-2-glycoprotein 1	APOH_HUMAN	VYKPSAGNNSLYR	155-167	TMT0 [N-Term]; HexNAc(4)Hex(5)Fuc(1)NeuAc(2) [N8]	HexNAc(4)Hex(5)Fuc(1)NeuAc(2)	162	2	4043.7371	0	0	4.70	352.7	173.6	6.9	2.01E-05	1.16E-06	0	0	7.87E-05	3.08E-05	2.91E-05	3.61E-04
Beta-2-glycoprotein 1	APOH_HUMAN	VYKPSAGNNSLYR	155-167	TMT0 [N-Term]; TMT0 [K3]; Deamidated [N9]; HexNAc(4)Hex(5)Fuc(1)NeuAc(2) [N8]	HexNAc(4)Hex(5)Fuc(1)NeuAc(2)	162	1	4268.8736	0	0	5.20	366.4	273.4	12.6	6.24E-06	4.18E-07	0	0	2.45E-05	1.64E-06	0	0
Beta-2-glycoprotein 1	APOH_HUMAN	VYKPSAGNNSLYR	155-167	TMT0 [N-Term]; TMT0 [K3]; HexNAc(4)Hex(5)Fuc(1)NeuAc(2) [N8]	HexNAc(4)Hex(5)Fuc(1)NeuAc(2)	162	3	4267.8896	0	0	5.69	373.8	326.9	13.9	2.05E-06	1.51E-07	0	0	8.05E-06	5.95E-07	0	0
Beta-2-glycoprotein 1	APOH_HUMAN	VYKPSAGNNSLYR	155-167	TMT0 [N-Term]; TMT0 [K3]; Deamidated [N9]; HexNAc(4)Hex(5)NeuAc(2) [N8]	HexNAc(4)Hex(5)NeuAc(2)	162	2	4122.8157	0	0	5.61	373.6	266.0	29.6	2.47E-06	1.82E-07	0	0	9.68E-06	7.14E-07	0	0
Beta-2-glycoprotein 1	APOH_HUMAN	VYKPSAGNNSLYR	155-167	TMT0 [N-Term]; Deamidated [N9]; HexNAc(4)Hex(5)NeuAc(2) [N8]	HexNAc(4)Hex(5)NeuAc(2)	162	3	3898.6632	0	0	7.60	427.2	354.2	123.7	2.51E-08	2.06E-09	0	0	9.83E-08	8.04E-09	0	0
Beta-2-glycoprotein 1	APOH_HUMAN	VYKPSAGNNSLYR	155-167	TMT0 [N-Term]; TMT0 [K3]; HexNAc(4)Hex(5)NeuAc(2) [N8]	HexNAc(4)Hex(5)NeuAc(2)	162	8	4121.8317	0	0	8.35	531.0	431.4	120.6	4.42E-09	4.72E-10	0	0	1.73E-08	1.85E-09	0	0
Beta-2-glycoprotein 1	APOH_HUMAN	VYKPSAGNNSLYR	155-167	TMT0 [N-Term]; HexNAc(4)Hex(5)NeuAc(2) [N8]	HexNAc(4)Hex(5)NeuAc(2)	162	8	3897.6792	0	0	9.27	519.5	423.9	143.5	5.31E-10	8.16E-11	0	0	2.08E-09	3.21E-10	0	0
Beta-2-glycoprotein 1	APOH_HUMAN	VYKPSAGNNSLYR	155-167	TMT0 [N-Term]; Deamidated [N9]; HexNAc(6)Hex(5)Fuc(1)NeuAc(1) [N8]	HexNAc(6)Hex(5)Fuc(1)NeuAc(1)	162	1	4159.7845	0	0	3.91	240.9	209.8	14.7	1.23E-04	2.83E-05	2.65E-05	3.32E-04	4.83E-04	3.08E-05	2.68E-05	3.32E-04
Beta-2-glycoprotein 1	APOH_HUMAN	LGNWSAMPSCK	251-261	TMT0 [N-Term]; TMT0 [K11]; Carbamidomethyl [C10]; HexNAc(4)Hex(5)NeuAc(1) [N3]	HexNAc(4)Hex(5)NeuAc(1)	253	3	3612.5476	0	0	4.20	295.2	235.2	109.6	6.29E-05	2.83E-05	2.73E-05	3.42E-04	2.46E-04	3.08E-05	2.76E-05	3.42E-04
Beta-2-glycoprotein 1	APOH_HUMAN	LGNWSAMPSCK	251-261	TMT0 [N-Term]; TMT0 [K11]; Oxidation [M7]; Carbamidomethyl [C10]; HexNAc(4)Hex(5)NeuAc(1) [N3]	HexNAc(4)Hex(5)NeuAc(1)	253	1	3628.5425	0	0	3.66	276.4	240.7	240.7	2.19E-04	1.30E-04	1.29E-04	3.24E-04	8.58E-04	5.33E-05	5.23E-05	3.24E-04
Beta-2-glycoprotein 1	APOH_HUMAN	LGNWSAMPSCK	251-261	TMT0 [N-Term]; TMT0 [K11]; Oxidation [M7]; Carbamidomethyl [C10]; HexNAc(4)Hex(5)NeuAc(2) [N3]	HexNAc(4)Hex(5)NeuAc(2)	253	4	3919.6380	0	0	4.94	340.9	237.8	237.8	1.15E-05	7.00E-07	0	0	4.53E-05	3.08E-05	3.00E-05	3.70E-04
Beta-2-glycoprotein 1	APOH_HUMAN	LGNWSAMPSCK	251-261	TMT0 [N-Term]; Carbamidomethyl [C10]; HexNAc(4)Hex(5)NeuAc(2) [N3]	HexNAc(4)Hex(5)NeuAc(2)	253	8	3679.4906	0	0	4.89	285.4	285.4	141.1	1.28E-05	7.61E-07	0	0	5.00E-05	3.08E-05	2.98E-05	3.68E-04
Beta-2-glycoprotein 1	APOH_HUMAN	LGNWSAMPSCK	251-261	TMT0 [N-Term]; Oxidation [M7]; Carbamidomethyl [C10]; HexNAc(4)Hex(5)NeuAc(2) [N3]	HexNAc(4)Hex(5)NeuAc(2)	253	5	3695.4855	0	0	4.37	276.6	222.2	222.2	4.23E-05	2.83E-05	2.78E-05	3.47E-04	1.66E-04	3.08E-05	2.80E-05	3.47E-04
Beta-2-glycoprotein 1	APOH_HUMAN	LGNWSAMPSCK	251-261	TMT0 [N-Term]; TMT0 [K11]; Carbamidomethyl [C10]; HexNAc(4)Hex(5)NeuAc(2) [N3]	HexNAc(4)Hex(5)NeuAc(2)	253	11	3903.6430	0	0	5.45	410.9	296.2	112.5	3.54E-06	2.55E-07	0	0	1.39E-05	1.00E-06	0	0
Beta-2-glycoprotein 1	APOH_HUMAN	LGNWSAMPSCK	251-261	TMT0 [N-Term]; TMT0 [K11]; Carbamidomethyl [C10]; HexNAc(4)Hex(6)Fuc(1)NeuAc(1) [N3]	HexNAc(4)Hex(6)Fuc(1)NeuAc(1)	253	1	3920.6584	0	0	2.01	249.6	244.9	244.9	9.68E-03	5.45E-04	5.33E-04	5.80E-04	2.64E-02	2.59E-04	2.57E-04	2.32E-03
Biglycan	PGS1_HUMAN	MIENGSLSFLPTLR	267-280	Oxidation [M1]; HexNAc(6)Hex(3)Fuc(1)NeuAc(1) [N4]	HexNAc(6)Hex(3)Fuc(1)NeuAc(1)	270	1	3735.6185	0	0	4.90	236.4	229.2	17.9	1.26E-05	7.49E-07	0	0	4.92E-05	3.08E-05	2.99E-05	3.68E-04
Biglycan	PGS1_HUMAN	MIENGSLSFLPTLR	267-280	TMT0 [N-Term]; Oxidation [M1]; HexNAc(6)Hex(3)Fuc(1)NeuAc(2) [N4]	HexNAc(6)Hex(3)Fuc(1)NeuAc(2)	270	6	4250.8664	0	0	5.49	300.4	280.9	280.9	3.21E-06	2.32E-07	0	0	1.26E-05	9.09E-07	0	0
Biglycan	PGS1_HUMAN	MIENGSLSFLPTLR	267-280	Oxidation [M1]; HexNAc(6)Hex(3)Fuc(1)NeuAc(2) [N4]	HexNAc(6)Hex(3)Fuc(1)NeuAc(2)	270	5	4026.7140	0	0	6.17	384.8	294.9	12.2	6.74E-07	4.81E-08	0	0	2.64E-06	1.90E-07	0	0
Biglycan	PGS1_HUMAN	LLQVVYLHSNNITK	301-314	TMT0 [N-Term]; HexNAc(3)Hex(11) [N11]	HexNAc(3)Hex(11)	311	5	4257.9039	0	1	4.61	344.8	286.7	13.7	2.44E-05	1.36E-06	0	0	9.57E-05	3.08E-05	2.88E-05	3.56E-04
Biglycan	PGS1_HUMAN	LLQVVYLHSNNITK	301-314	TMT0 [N-Term]; HexNAc(4)Hex(5)Fuc(1)NeuAc(2) [N11]	HexNAc(4)Hex(5)Fuc(1)NeuAc(2)	311	2	4216.9151	0	0	5.16	384.8	286.0	2.4	6.88E-06	4.54E-07	0	0	2.70E-05	1.79E-06	0	0
Biglycan	PGS1_HUMAN	LLQVVYLHSNNITK	301-314	TMT0 [N-Term]; HexNAc(5)Hex(4)Fuc(1)NeuAc(1) [N11]	HexNAc(5)Hex(4)Fuc(1)NeuAc(1)	311	2	3966.8462	0	0	4.17	251.0	227.1	227.1	6.83E-05	2.83E-05	2.72E-05	3.41E-04	2.68E-04	3.08E-05	2.74E-05	3.41E-04
Biglycan	PGS1_HUMAN	LLQVVYLHSNNITK	301-314	TMT0 [N-Term]; HexNAc(5)Hex(4)Fuc(1)NeuAc(2) [N11]	HexNAc(5)Hex(4)Fuc(1)NeuAc(2)	311	1	4257.9416	0	0	5.11	362.5	298.3	7.9	7.78E-06	5.05E-07	0	0	3.05E-05	3.08E-05	3.07E-05	3.77E-04
Biglycan	PGS1_HUMAN	LLQVVYLHSNNITK	301-314	TMT0 [N-Term]; Deamidated [N10]; HexNAc(5)Hex(4)Fuc(1)NeuAc(2) [N11]	HexNAc(5)Hex(4)Fuc(1)NeuAc(2)	311	3	4258.9257	0	-1	2.92	331.4	187.4	8.9	1.19E-03	2.78E-04	2.77E-04	3.15E-04	3.41E-03	5.33E-05	5.09E-05	3.15E-04
Biglycan	PGS1_HUMAN	LLQVVYLHSNNITK	301-314	TMT0 [N-Term]; HexNAc(6)Hex(3)Fuc(1)NeuAc(1) [N11]	HexNAc(6)Hex(3)Fuc(1)NeuAc(1)	311	2	4007.8728	0	0	4.60	294.2	226.9	91.9	2.52E-05	1.39E-06	0	0	9.90E-05	3.08E-05	2.88E-05	3.56E-04
Biglycan	PGS1_HUMAN	LLQVVYLHSNNITK	301-314	TMT0 [N-Term]; Deamidated [N10]; HexNAc(6)Hex(3)Fuc(1)NeuAc(1) [N11]	HexNAc(6)Hex(3)Fuc(1)NeuAc(1)	311	2	4008.8568	0	1	5.41	354.6	285.1	285.1	3.92E-06	2.80E-07	0	0	1.54E-05	1.10E-06	0	0
Biglycan	PGS1_HUMAN	LLQVVYLHSNNITK	301-314	TMT0 [N-Term]; HexNAc(6)Hex(3)Fuc(1)NeuAc(2) [N11]	HexNAc(6)Hex(3)Fuc(1)NeuAc(2)	311	13	4298.9682	0	0	5.62	382.0	298.3	112.4	2.42E-06	1.78E-07	0	0	9.49E-06	6.99E-07	0	0
Biglycan	PGS1_HUMAN	LLQVVYLHSNNITK	301-314	TMT0 [N-Term]; Deamidated [N10]; HexNAc(6)Hex(3)Fuc(1)NeuAc(2) [N11]	HexNAc(6)Hex(3)Fuc(1)NeuAc(2)	311	3	4299.9522	0	0	5.80	396.6	327.1	1.5	1.60E-06	1.18E-07	0	0	6.26E-06	4.63E-07	0	0
Biglycan	PGS1_HUMAN	LLQVVYLHSNNITK	301-314	TMT0 [N-Term]; HexNAc(7)Hex(6) [N11]	HexNAc(7)Hex(6)	311	2	4259.9573	0	-1	2.06	280.2	233.9	5.2	8.65E-03	5.45E-04	5.41E-04	5.90E-04	2.30E-02	1.67E-04	1.66E-04	1.77E-03
Biglycan	PGS1_HUMAN	LLQVVYLHSNNITK	301-314	TMT0 [N-Term]; TMT0 [K14]; HexNAc(8)Hex(5) [N11]	HexNAc(8)Hex(5)	311	1	4525.1363	0	-1	3.97	357.0	238.1	5.9	1.08E-04	2.83E-05	2.67E-05	3.34E-04	4.22E-04	3.08E-05	2.69E-05	3.34E-04
Biglycan	PGS1_HUMAN	LLQVVYLHSNNITK	301-314	TMT0 [N-Term]; HexNAc(8)Hex(5) [N11]	HexNAc(8)Hex(5)	311	2	4300.9838	0	-1	3.22	377.6	318.8	20.1	6.09E-04	2.30E-04	2.29E-04	3.19E-04	1.92E-03	5.33E-05	5.15E-05	3.19E-04
Cathepsin D	CATD_HUMAN	GSLSYLNVTR	257-266	TMT0 [N-Term]; HexNAc(2)Hex(6) [N7]	HexNAc(2)Hex(6)	263	1	2712.2231	0	0	6.99	420.7	420.7	420.7	1.01E-07	6.36E-09	0	0	3.98E-07	2.49E-08	0	0
Clusterin	CLUS_HUMAN	LANLTQGEDQYYLR	372-385	HexNAc(2)Hex(12) [N3]	HexNAc(2)Hex(12)	374	1	4034.6263	0	0	2.09	250.4	136.0	6.9	8.19E-03	5.22E-04	5.22E-04	5.96E-04	2.13E-02	1.45E-04	1.44E-04	1.49E-03
Clusterin	CLUS_HUMAN	LANLTQGEDQYYLR	372-385	TMT0 [N-Term]; HexNAc(4)Hex(5)Fuc(1)NeuAc(2) [N3]	HexNAc(4)Hex(5)Fuc(1)NeuAc(2)	374	4	4258.8165	0	0	4.54	272.1	219.3	23.0	2.91E-05	1.58E-06	0	0	1.14E-04	3.08E-05	2.85E-05	3.53E-04
Clusterin	CLUS_HUMAN	LANLTQGEDQYYLR	372-385	TMT0 [N-Term]; HexNAc(4)Hex(5)NeuAc(2) [N3]	HexNAc(4)Hex(5)NeuAc(2)	374	1	4112.7586	0	0	2.35	255.4	173.5	51.1	4.43E-03	3.91E-04	3.90E-04	3.06E-04	1.15E-02	7.54E-05	7.39E-05	6.12E-04
Collagen alpha-1(XVIII) chain	COIA1_HUMAN	FGVNSSDVPGPAGLPGVPGR	923-942	TMT0 [N-Term]; Hydroxyproline [P15]; Hydroxyproline [P18]; HexNAc(4)Hex(5)Fuc(1)NeuAc(1) [N4]	HexNAc(4)Hex(5)Fuc(1)NeuAc(1)	926	1	4195.8433	0	0	3.84	231.5	165.7	16.7	1.44E-04	2.83E-05	2.63E-05	3.29E-04	5.65E-04	5.33E-05	5.31E-05	3.29E-04
Collagen alpha-1(XVIII) chain	COIA1_HUMAN	FGVNSSDVPGPAGLPGVPGR	923-942	TMT0 [N-Term]; Hydroxyproline [P15]; Hydroxyproline [P18]; HexNAc(4)Hex(5)Fuc(1)NeuAc(2) [N4]	HexNAc(4)Hex(5)Fuc(1)NeuAc(2)	926	1	4486.9387	0	0	4.31	291.0	163.1	0.4	4.94E-05	2.83E-05	2.76E-05	3.45E-04	1.94E-04	3.08E-05	2.78E-05	3.45E-04
Collagen alpha-1(XVIII) chain	COIA1_HUMAN	FGVNSSDVPGPAGLPGVPGR	923-942	TMT0 [N-Term]; Hydroxyproline [P18]; HexNAc(4)Hex(6)NeuAc(1) [N4]	HexNAc(4)Hex(6)NeuAc(1)	926	1	4195.8433	0	0	2.06	214.0	164.8	10.1	8.80E-03	5.45E-04	5.39E-04	5.87E-04	2.35E-02	2.13E-04	2.13E-04	2.05E-03
Collagen alpha-1(XVIII) chain	COIA1_HUMAN	FGVNSSDVPGPAGLPGVPGR	923-942	TMT0 [N-Term]; Hydroxyproline [P18]; HexNAc(4)Hex(6)NeuAc(2) [N4]	HexNAc(4)Hex(6)NeuAc(2)	926	3	4486.9387	0	0	4.23	263.6	202.0	38.0	5.93E-05	2.83E-05	2.74E-05	3.42E-04	2.32E-04	3.08E-05	2.76E-05	3.42E-04
Complement factor B	CFAB_HUMAN	GSANR	139-143	TMT0 [N-Term]; HexNAc(4)Hex(5)NeuAc(2) [N4]	HexNAc(4)Hex(5)NeuAc(2)	142	1	2933.1774	0	0	3.81	245.1	245.1	245.1	1.55E-04	5.26E-05	5.25E-05	3.28E-04	6.07E-04	5.33E-05	5.30E-05	3.28E-04
Complement factor H	CFAH_HUMAN	IPCSQPPQIEHGTINSSR	868-885	TMT0 [N-Term]; Deamidated [Q8]; Carbamidomethyl [C3]; HexNAc(4)Hex(5)NeuAc(2) [N15]	HexNAc(4)Hex(5)NeuAc(2)	882	2	4450.8958	0	-1	4.50	396.1	288.5	34.9	3.13E-05	2.83E-05	2.82E-05	3.52E-04	1.23E-04	3.08E-05	2.84E-05	3.52E-04
Complement factor H	CFAH_HUMAN	IPCSQPPQIEHGTINSSR	868-885	TMT0 [N-Term]; Carbamidomethyl [C3]; HexNAc(4)Hex(5)NeuAc(2) [N15]	HexNAc(4)Hex(5)NeuAc(2)	882	16	4449.9118	0	0	6.57	381.2	350.6	170.6	2.67E-07	1.72E-08	0	0	1.05E-06	6.79E-08	0	0
Complement factor H	CFAH_HUMAN	ISEENETTCYMGK	907-919	TMT0 [N-Term]; TMT0 [K13]; Carbamidomethyl [C9]; HexNAc(4)Hex(5)NeuAc(2) [N5]	HexNAc(4)Hex(5)NeuAc(2)	911	3	4214.7283	0	0	8.44	521.2	451.8	184.2	3.66E-09	3.93E-10	0	0	1.43E-08	1.53E-09	0	0
Complement factor H	CFAH_HUMAN	ISEENETTCYMGK	907-919	TMT0 [N-Term]; TMT0 [K13]; Carbamidomethyl [C9]; HexNAc(4)Hex(6)Fuc(1)NeuAc(1) [N5]	HexNAc(4)Hex(6)Fuc(1)NeuAc(1)	911	1	4231.7436	0	-1	4.20	299.2	251.5	31.0	6.29E-05	2.83E-05	2.73E-05	3.42E-04	2.47E-04	3.08E-05	2.76E-05	3.42E-04
Fibrillin-1	FBN1_HUMAN	VLPVNVTDYCQLVR	444-457	TMT0 [N-Term]; Deamidated [Q11]; Carbamidomethyl [C10]; HexNAc(2)Hex(2) [N5]	HexNAc(2)Hex(2)	448	1	2631.2845	0	0	3.62	131.1	124.4	124.4	2.39E-04	1.30E-04	1.29E-04	3.23E-04	9.36E-04	5.33E-05	5.21E-05	3.23E-04
Fibrinogen beta chain	FIBB_HUMAN	GTAGNALMDGASQLMGENR	377-395	TMT0 [N-Term]; Oxidation [M15]; HexNAc(2)Hex(11) [N18]	HexNAc(2)Hex(11)	394	1	4321.7461	0	-1	3.66	334.7	203.4	7.5	2.16E-04	1.30E-04	1.30E-04	3.25E-04	8.48E-04	5.33E-05	5.23E-05	3.24E-04
Fibrinogen beta chain	FIBB_HUMAN	GTAGNALMDGASQLMGENR	377-395	TMT0 [N-Term]; HexNAc(4)Hex(5)NeuAc(1) [N18]	HexNAc(4)Hex(5)NeuAc(1)	394	1	4030.6884	0	0	5.51	343.1	329.4	26.5	3.07E-06	2.23E-07	0	0	1.20E-05	8.76E-07	0	0
Fibrinogen beta chain	FIBB_HUMAN	GTAGNALMDGASQLMGENR	377-395	TMT0 [N-Term]; HexNAc(4)Hex(5)NeuAc(2) [N18]	HexNAc(4)Hex(5)NeuAc(2)	394	2	4321.7838	0	0	6.92	445.0	344.9	2.7	1.20E-07	7.48E-09	0	0	4.71E-07	2.93E-08	0	0
Fibromodulin	FMOD_HUMAN	LYLDHNNLTR	160-169	TMT0 [N-Term]; HexNAc(2)Hex(12) [N7]	HexNAc(2)Hex(12)	166	2	3833.5990	0	0	4.13	439.6	328.2	11.2	7.36E-05	2.83E-05	2.71E-05	3.39E-04	2.89E-04	3.08E-05	2.74E-05	3.39E-04
Fibromodulin	FMOD_HUMAN	LYLDHNNLTR	160-169	TMT0 [N-Term]; Deamidated [N6]; HexNAc(2)Hex(12) [N7]	HexNAc(2)Hex(12)	166	1	3834.5830	0	0	3.27	378.7	313.0	2.0	5.42E-04	2.30E-04	2.30E-04	3.20E-04	1.74E-03	5.33E-05	5.16E-05	3.19E-04
Fibromodulin	FMOD_HUMAN	LYLDHNNLTR	160-169	HexNAc(4)Hex(5)Fuc(1)NeuAc(1) [N7]	HexNAc(4)Hex(5)Fuc(1)NeuAc(1)	166	1	3318.3888	0	0	2.33	171.1	38.9	38.9	4.63E-03	4.14E-04	4.14E-04	3.06E-04	1.21E-02	7.54E-05	7.38E-05	6.12E-04
Fibromodulin	FMOD_HUMAN	LYLDHNNLTR	160-169	TMT0 [N-Term]; HexNAc(4)Hex(5)Fuc(1)NeuAc(1) [N7]	HexNAc(4)Hex(5)Fuc(1)NeuAc(1)	166	3	3542.5413	0	0	5.72	446.2	327.7	327.7	1.91E-06	1.41E-07	0	0	7.48E-06	5.53E-07	0	0
Fibromodulin	FMOD_HUMAN	LYLDHNNLTR	160-169	TMT0 [N-Term]; HexNAc(4)Hex(5)Fuc(1)NeuAc(2) [N7]	HexNAc(4)Hex(5)Fuc(1)NeuAc(2)	166	18	3833.6367	0	0	6.36	514.8	353.2	16.4	4.36E-07	2.94E-08	0	0	1.71E-06	1.16E-07	0	0
Fibromodulin	FMOD_HUMAN	LYLDHNNLTR	160-169	TMT0 [N-Term]; Deamidated [N6]; HexNAc(4)Hex(5)Fuc(1)NeuAc(2) [N7]	HexNAc(4)Hex(5)Fuc(1)NeuAc(2)	166	4	3834.6207	0	0	7.53	469.9	395.8	23.9	2.97E-08	2.33E-09	0	0	1.17E-07	9.10E-09	0	0
Fibromodulin	FMOD_HUMAN	LYLDHNNLTR	160-169	TMT0 [N-Term]; HexNAc(5)Hex(4)Fuc(1)NeuAc(2) [N7]	HexNAc(5)Hex(4)Fuc(1)NeuAc(2)	166	2	3874.6632	0	0	7.72	500.1	382.4	4.6	1.90E-08	1.64E-09	0	0	7.46E-08	6.44E-09	0	0
Fibronectin	FINC_HUMAN	DQCIVDDITYNVNDTFHK	516-533	TMT0 [N-Term]; Carbamidomethyl [C3]; HexNAc(4)Hex(5)Fuc(1)NeuAc(1) [N13]	HexNAc(4)Hex(5)Fuc(1)NeuAc(1)	528	1	4480.8740	0	0	4.47	301.0	172.8	58.4	3.37E-05	2.83E-05	2.81E-05	3.51E-04	1.32E-04	3.08E-05	2.83E-05	3.51E-04
Fibronectin	FINC_HUMAN	DQCIVDDITYNVNDTFHK	516-533	TMT0 [N-Term]; TMT0 [K18]; Carbamidomethyl [C3]; HexNAc(4)Hex(5)Fuc(1)NeuAc(1) [N13]	HexNAc(4)Hex(5)Fuc(1)NeuAc(1)	528	2	4705.0265	0	0	5.44	301.1	236.1	50.0	3.62E-06	2.61E-07	0	0	1.42E-05	1.02E-06	0	0
Fibronectin	FINC_HUMAN	LDAPTNLQFVNETDSTVLVR	997-1016	TMT0 [N-Term]; HexNAc(4)Hex(5)Fuc(1)NeuAc(1) [N11]	HexNAc(4)Hex(5)Fuc(1)NeuAc(1)	1007	2	4516.0381	0	0	4.92	330.6	192.3	19.1	1.19E-05	7.22E-07	0	0	4.69E-05	3.08E-05	2.99E-05	3.69E-04
Fibronectin	FINC_HUMAN	LDAPTNLQFVNETDSTVLVR	997-1016	HexNAc(4)Hex(5)Fuc(1)NeuAc(1) [N11]	HexNAc(4)Hex(5)Fuc(1)NeuAc(1)	1007	1	4291.8856	0	-1	2.75	289.9	125.5	125.5	1.79E-03	2.78E-04	2.75E-04	3.13E-04	4.96E-03	5.33E-05	5.04E-05	3.13E-04
Fibronectin	FINC_HUMAN	LDAPTNLQFVNETDSTVLVR	997-1016	HexNAc(4)Hex(6)NeuAc(1) [N11]	HexNAc(4)Hex(6)NeuAc(1)	1007	1	4307.8805	0	0	4.26	286.4	147.8	97.4	5.44E-05	2.83E-05	2.75E-05	3.43E-04	2.13E-04	3.08E-05	2.77E-05	3.43E-04
Galectin-3-binding protein	LG3BP_HUMAN	ALGFENATQALGR	64-76	TMT0 [N-Term]; HexNAc(4)Hex(5)Fuc(1)NeuAc(2) [N6]	HexNAc(4)Hex(5)Fuc(1)NeuAc(2)	69	3	3922.6844	0	0	5.47	373.5	242.6	9.4	3.41E-06	2.46E-07	0	0	1.34E-05	9.65E-07	0	0
Haptoglobin	HPT_HUMAN	VVLHPNYSQVDIGLIK	236-251	TMT0 [N-Term]; HexNAc(4)Hex(5)NeuAc(2) [N6]	HexNAc(4)Hex(5)NeuAc(2)	241	1	4223.9362	0	0	3.47	55.4	55.4	55.4	3.36E-04	1.80E-04	1.80E-04	3.21E-04	1.21E-03	5.33E-05	5.19E-05	3.21E-04
Haptoglobin	HPT_HUMAN	VVLHPNYSQVDIGLIK	236-251	TMT0 [K16]; HexNAc(4)Hex(5)NeuAc(2) [N6]	HexNAc(4)Hex(5)NeuAc(2)	241	1	4223.9362	0	0	5.42	405.1	185.5	121.9	3.82E-06	2.73E-07	0	0	1.50E-05	1.07E-06	0	0
Haptoglobin	HPT_HUMAN	VVLHPNYSQVDIGLIK	236-251	TMT0 [N-Term]; HexNAc(4)Hex(6)Fuc(1)NeuAc(1) [N6]	HexNAc(4)Hex(6)Fuc(1)NeuAc(1)	241	1	4240.9515	0	-1	2.86	315.0	216.5	216.5	1.37E-03	2.78E-04	2.76E-04	3.14E-04	3.89E-03	5.33E-05	5.07E-05	3.14E-04
Haptoglobin	HPT_HUMAN	VVLHPNYSQVDIGLIK	236-251	TMT0 [N-Term]; HexNAc(5)Hex(6)NeuAc(3) [N6]	HexNAc(5)Hex(6)NeuAc(3)	241	1	4880.1638	0	0	2.33	189.9	184.8	19.9	4.72E-03	4.14E-04	4.14E-04	3.06E-04	1.23E-02	7.54E-05	7.38E-05	6.11E-04
Hemopexin	HEMO_HUMAN	SWPAVGNCSSALR	181-193	TMT0 [N-Term]; Carbamidomethyl [C8]; HexNAc(4)Hex(5)Fuc(1)NeuAc(2) [N7]	HexNAc(4)Hex(5)Fuc(1)NeuAc(2)	187	4	3979.6517	0	0	6.63	415.5	365.4	6.9	2.37E-07	1.50E-08	0	0	9.29E-07	5.91E-08	0	0
Hemopexin	HEMO_HUMAN	SWPAVGNCSSALR	181-193	TMT0 [N-Term]; Carbamidomethyl [C8]; HexNAc(4)Hex(5)NeuAc(2) [N7]	HexNAc(4)Hex(5)NeuAc(2)	187	24	3833.5938	0	0	11.01	585.6	542.4	542.4	9.74E-12	8.17E-13	0	0	3.82E-11	3.16E-12	0	0
Hemopexin	HEMO_HUMAN	ALPQPQNVTSLLGCTH	447-462	TMT0 [N-Term]; Carbamidomethyl [C14]; HexNAc(4)Hex(5)NeuAc(2) [N7]	HexNAc(4)Hex(5)NeuAc(2)	453	9	4164.8045	0	0	6.15	306.9	306.9	87.7	7.04E-07	5.08E-08	0	0	2.76E-06	2.00E-07	0	0
Hemopexin	HEMO_HUMAN	ALPQPQNVTSLLGCTH	447-462	TMT0 [N-Term]; Carbamidomethyl [C14]; HexNAc(4)Hex(6)Fuc(1)NeuAc(1) [N7]	HexNAc(4)Hex(6)Fuc(1)NeuAc(1)	453	1	4181.8198	0	-1	2.02	210.1	210.1	210.1	9.58E-03	5.45E-04	5.33E-04	5.81E-04	2.61E-02	2.59E-04	2.58E-04	2.32E-03
Hyaluronan and proteoglycan link protein 1	HPLN1_HUMAN	GGNVTLPCK	54-62	TMT0 [N-Term]; TMT0 [K9]; Carbamidomethyl [C8]; HexNAc(4)Hex(5)Fuc(1)NeuAc(1) [N3]	HexNAc(4)Hex(5)Fuc(1)NeuAc(1)	56	4	3453.5221	0	0	4.45	262.3	207.5	207.5	3.58E-05	2.83E-05	2.80E-05	3.49E-04	1.40E-04	3.08E-05	2.82E-05	3.49E-04
Hyaluronan and proteoglycan link protein 1	HPLN1_HUMAN	GGNVTLPCK	54-62	TMT0 [N-Term]; TMT0 [K9]; Carbamidomethyl [C8]; HexNAc(4)Hex(5)Fuc(1)NeuAc(2) [N3]	HexNAc(4)Hex(5)Fuc(1)NeuAc(2)	56	10	3744.6175	0	0	4.15	270.5	210.3	29.9	7.13E-05	2.83E-05	2.71E-05	3.40E-04	2.80E-04	3.08E-05	2.74E-05	3.40E-04
Hyaluronan and proteoglycan link protein 1	HPLN1_HUMAN	GGNVTLPCK	54-62	TMT0 [N-Term]; TMT0 [K9]; Carbamidomethyl [C8]; HexNAc(5)Hex(4)Fuc(1)NeuAc(2) [N3]	HexNAc(5)Hex(4)Fuc(1)NeuAc(2)	56	2	3785.6441	0	0	3.31	228.0	194.7	4.6	4.92E-04	2.05E-04	2.05E-04	3.20E-04	1.61E-03	5.33E-05	5.17E-05	3.20E-04
Ig gamma-1 chain C region	IGHG1_HUMAN	TKPREEQYNSTYR	172-184	TMT0 [N-Term]; HexNAc(4)Hex(3)Fuc(1) [N9]	HexNAc(4)Hex(3)Fuc(1)	180	3	3340.4949	1	0	3.91	348.1	7.4	7.4	1.24E-04	2.83E-05	2.65E-05	3.32E-04	4.85E-04	3.08E-05	2.67E-05	3.32E-04
Ig gamma-1 chain C region	IGHG1_HUMAN	TKPREEQYNSTYR	172-184	TMT0 [N-Term]; HexNAc(4)Hex(4)Fuc(1) [N9]	HexNAc(4)Hex(4)Fuc(1)	180	1	3502.5477	1	0	2.08	248.2	14.6	14.6	8.27E-03	5.22E-04	5.21E-04	5.95E-04	2.16E-02	1.45E-04	1.44E-04	1.49E-03
Ig gamma-1 chain C region	IGHG1_HUMAN	TKPREEQYNSTYR	172-184	TMT0 [N-Term]; TMT0 [K2]; HexNAc(4)Hex(4)Fuc(1) [N9]	HexNAc(4)Hex(4)Fuc(1)	180	1	3726.7002	1	0	2.63	313.2	12.8	12.8	2.35E-03	2.98E-04	2.97E-04	3.11E-04	6.39E-03	7.54E-05	7.51E-05	6.22E-04
Ig gamma-1 chain C region	IGHG1_HUMAN	TKPREEQYNSTYR	172-184	TMT0 [N-Term]; HexNAc(4)Hex(5)Fuc(1) [N9]	HexNAc(4)Hex(5)Fuc(1)	180	2	3664.6005	1	0	2.11	232.5	0.0	0.0	7.76E-03	4.57E-04	4.55E-04	6.02E-04	1.97E-02	1.45E-04	1.45E-04	1.50E-03
Ig gamma-1 chain C region	IGHG1_HUMAN	EEQYNSTYR	176-184	TMT0 [N-Term]; HexNAc(4)Hex(3) [N5]	HexNAc(4)Hex(3)	180	2	2712.1405	0	0	4.80	292.0	291.5	291.5	1.59E-05	9.34E-07	0	0	6.24E-05	3.08E-05	2.95E-05	3.64E-04
Ig gamma-1 chain C region	IGHG1_HUMAN	EEQYNSTYR	176-184	TMT0 [N-Term]; HexNAc(4)Hex(3)Fuc(1) [N5]	HexNAc(4)Hex(3)Fuc(1)	180	2	2858.1984	0	0	2.09	250.2	54.6	54.6	8.10E-03	4.77E-04	4.75E-04	5.98E-04	2.10E-02	1.45E-04	1.44E-04	1.49E-03
Ig gamma-1 chain C region	IGHG1_HUMAN	EEQYNSTYR	176-184	TMT0 [N-Term]; HexNAc(4)Hex(4) [N5]	HexNAc(4)Hex(4)	180	3	2874.1933	0	0	4.97	287.0	287.0	287.0	1.08E-05	6.59E-07	0	0	4.22E-05	3.08E-05	3.01E-05	3.71E-04
Ig gamma-1 chain C region	IGHG1_HUMAN	EEQYNSTYR	176-184	TMT0 [N-Term]; HexNAc(4)Hex(5) [N5]	HexNAc(4)Hex(5)	180	1	3036.2461	0	0	2.16	212.4	212.4	212.4	6.89E-03	4.36E-04	4.34E-04	6.05E-04	1.75E-02	9.77E-05	9.73E-05	9.07E-04
Ig gamma-2 chain C region	IGHG2_HUMAN	TKPREEQFNSTFR	168-180	TMT0 [N-Term]; HexNAc(4)Hex(3)Fuc(1) [N9]	HexNAc(4)Hex(3)Fuc(1)	176	1	3308.5050	1	0	5.03	225.2	225.2	99.2	9.33E-06	5.91E-07	0	0	3.66E-05	3.08E-05	3.03E-05	3.73E-04
Ig gamma-2 chain C region	IGHG2_HUMAN	TKPREEQFNSTFR	168-180	HexNAc(4)Hex(3)Fuc(1) [N9]	HexNAc(4)Hex(3)Fuc(1)	176	1	3084.3526	1	0	3.95	205.6	204.1	204.1	1.13E-04	2.83E-05	2.66E-05	3.33E-04	4.43E-04	3.08E-05	2.69E-05	3.33E-04
Ig gamma-2 chain C region	IGHG2_HUMAN	TKPREEQFNSTFR	168-180	TMT0 [N-Term]; TMT0 [K2]; HexNAc(4)Hex(3)Fuc(1) [N9]	HexNAc(4)Hex(3)Fuc(1)	176	1	3532.6575	1	0	5.93	272.3	272.3	272.3	1.17E-06	8.59E-08	0	0	4.57E-06	3.38E-07	0	0
Ig gamma-2 chain C region	IGHG2_HUMAN	TKPREEQFNSTFR	168-180	TMT0 [N-Term]; HexNAc(4)Hex(4)Fuc(1) [N9]	HexNAc(4)Hex(4)Fuc(1)	176	1	3470.5579	1	0	2.19	208.5	167.6	102.6	6.47E-03	4.36E-04	4.35E-04	6.06E-04	1.65E-02	9.77E-05	9.75E-05	9.09E-04

Ig gamma-2 chain C region	IGHG2_HUMAN	EEQFNSTFR	172-180	TMT0 [N-Term]; HexNAc(4)Hex(3)Fuc(1) [N5]	HexNAc(4)Hex(3)Fuc(1)	176	9	2826.2085	0	0	5.72	321.1	321.1	321.1	1.91E-06	1.41E-07	0	0	7.48E-06	5.54E-07	0	0
Ig gamma-2 chain C region	IGHG2_HUMAN	EEQFNSTFR	172-180	TMT0 [N-Term]; HexNAc(4)Hex(4)Fuc(1)NeuAc(1) [N5]	HexNAc(4)Hex(4)Fuc(1)NeuAc(1)	176	1	3279.3568	0	0	4.06	231.4	223.9	223.9	8.68E-05	2.83E-05	2.69E-05	3.37E-04	3.40E-04	3.08E-05	2.72E-05	3.37E-04
Ig gamma-2 chain C region	IGHG2_HUMAN	EEQFNSTFR	172-180	TMT0 [N-Term]; HexNAc(4)Hex(5)Fuc(1) [N5]	HexNAc(4)Hex(5)Fuc(1)	176	1	3150.3142	0	0	2.04	209.3	199.0	199.0	9.15E-03	5.45E-04	5.36E-04	5.84E-04	2.47E-02	2.59E-04	2.59E-04	2.33E-03
Ig gamma-2 chain C region	IGHG2_HUMAN	EEQFNSTFR	172-180	TMT0 [N-Term]; HexNAc(6)Hex(7)Fuc(1) [N5]	HexNAc(6)Hex(7)Fuc(1)	176	1	3880.5786	0	0	3.32	253.1	208.8	208.8	4.81E-04	2.05E-04	2.05E-04	3.20E-04	1.58E-03	5.33E-05	5.17E-05	3.20E-04
Ig gamma-4 chain C region	IGHG4_HUMAN	TKPREEQFNSTYR	169-181	TMT0 [N-Term]; HexNAc(4)Hex(4) [N9]	HexNAc(4)Hex(4)	177	4	3340.4949	1	0	4.09	342.0	4.5	4.5	8.04E-05	2.83E-05	2.70E-05	3.38E-04	3.15E-04	3.08E-05	2.72E-05	3.38E-04
Ig gamma-4 chain C region	IGHG4_HUMAN	TKPREEQFNSTYR	169-181	TMT0 [N-Term]; TMT0 [K2]; HexNAc(4)Hex(5) [N9]	HexNAc(4)Hex(5)	177	2	3726.7002	1	0	2.65	274.9	0.0	0.0	2.23E-03	2.78E-04	2.73E-04	3.12E-04	6.10E-03	7.54E-05	7.52E-05	6.23E-04
Ig gamma-4 chain C region	IGHG4_HUMAN	TKPREEQFNSTYR	169-181	TMT0 [N-Term]; HexNAc(4)Hex(5) [N9]	HexNAc(4)Hex(5)	177	2	3502.5477	1	0	2.61	259.1	0.0	0.0	2.45E-03	2.98E-04	2.97E-04	3.10E-04	6.66E-03	7.54E-05	7.50E-05	6.21E-04
Ig mu heavy chain disease protein	MUCB_HUMAN	GLTFQQNASSMCGPDQDTAIR	141-161	TMT0 [N-Term]; Carbamidomethyl [C12]; HexNAc(6)Hex(4)Fuc(1)NeuAc(1) [N7]	HexNAc(6)Hex(4)Fuc(1)NeuAc(1)	147	1	4825.0218	0	1	3.34	222.1	220.9	54.9	4.60E-04	2.05E-04	2.05E-04	3.21E-04	1.53E-03	5.33E-05	5.17E-05	3.20E-04
Insulin-like growth factor-binding protein 7	IBP7_HUMAN	DIWNVTGAQVYLSCEVIGIPTPVLIWNK	168-195	TMT0 [N-Term]; TMT0 [K28]; Carbamidomethyl [C14]; HexNAc(4)Hex(5)NeuAc(2) [N4]	HexNAc(4)Hex(5)NeuAc(2)	171	1	5838.7478	0	1	3.24	362.9	177.3	40.8	5.77E-04	2.30E-04	2.30E-04	3.19E-04	1.83E-03	5.33E-05	5.15E-05	3.19E-04
Lactadherin	MFGM_HUMAN	VAYSNDSANWTEYQDPR	321-337	TMT0 [N-Term]; HexNAc(4)Hex(5)Fuc(1)NeuAc(1) [N5]	HexNAc(4)Hex(5)Fuc(1)NeuAc(1)	325	1	4299.7604	0	0	6.46	393.7	327.4	34.9	3.46E-07	2.27E-08	0	0	1.36E-06	8.96E-08	0	0
Lumican	LUM_HUMAN	LHINHNNLTESVGPLPK	121-137	TMT0 [N-Term]; Deamidated [N6]; HexNAc(4)Hex(5)Fuc(1)NeuAc(1) [N7]	HexNAc(4)Hex(5)Fuc(1)NeuAc(1)	127	2	4167.8848	0	0	5.57	316.4	242.9	56.0	2.71E-06	1.99E-07	0	0	1.06E-05	7.82E-07	0	0
Lumican	LUM_HUMAN	LHINHNNLTESVGPLPK	121-137	HexNAc(4)Hex(5)Fuc(1)NeuAc(1) [N7]	HexNAc(4)Hex(5)Fuc(1)NeuAc(1)	127	3	3942.7483	0	0	5.72	241.3	241.3	241.3	1.91E-06	1.41E-07	0	0	7.50E-06	5.56E-07	0	0
Lumican	LUM_HUMAN	LHINHNNLTESVGPLPK	121-137	TMT0 [N-Term]; HexNAc(4)Hex(5)Fuc(1)NeuAc(1) [N7]	HexNAc(4)Hex(5)Fuc(1)NeuAc(1)	127	6	4166.9008	0	0	6.92	390.0	307.6	132.9	1.19E-07	7.44E-09	0	0	4.68E-07	2.92E-08	0	0
Lumican	LUM_HUMAN	LHINHNNLTESVGPLPK	121-137	TMT0 [N-Term]; Deamidated [N4]; HexNAc(4)Hex(5)Fuc(1)NeuAc(2) [N7]	HexNAc(4)Hex(5)Fuc(1)NeuAc(2)	127	1	4234.8277	0	-1	4.16	272.6	204.5	25.9	6.96E-05	2.83E-05	2.72E-05	3.40E-04	2.73E-04	3.08E-05	2.74E-05	3.40E-04
Lumican	LUM_HUMAN	LHINHNNLTESVGPLPK	121-137	HexNAc(4)Hex(5)Fuc(1)NeuAc(2) [N7]	HexNAc(4)Hex(5)Fuc(1)NeuAc(2)	127	2	4233.8437	0	-1	3.68	249.2	153.5	12.4	2.09E-04	7.80E-05	7.78E-05	3.25E-04	8.20E-04	5.33E-05	5.24E-05	3.25E-04
Lumican	LUM_HUMAN	LHINHNNLTESVGPLPK	121-137	TMT0 [N-Term]; HexNAc(4)Hex(5)Fuc(1)NeuAc(2) [N7]	HexNAc(4)Hex(5)Fuc(1)NeuAc(2)	127	3	4457.9962	0	0	6.87	363.5	315.5	22.3	1.35E-07	8.45E-09	0	0	5.29E-07	3.32E-08	0	0
Lumican	LUM_HUMAN	LHINHNNLTESVGPLPK	121-137	TMT0 [N-Term]; HexNAc(6)Hex(7) [N7]	HexNAc(6)Hex(7)	127	1	4460.0119	0	-1	3.89	267.1	210.3	24.3	1.27E-04	2.83E-05	2.65E-05	3.31E-04	5.00E-04	3.08E-05	2.67E-05	3.31E-04
Lysosome-associated membrane glycoprotein 1	LAMP1_HUMAN	GHTLTLNFTR	97-106	TMT0 [N-Term]; HexNAc(2)Hex(5) [N7]	HexNAc(2)Hex(5)	103	7	2600.1972	0	0	9.00	445.9	445.9	445.9	9.91E-10	1.35E-10	0	0	3.89E-09	5.23E-10	0	0
Lysosome-associated membrane glycoprotein 1	LAMP1_HUMAN	GHTLTLNFTR	97-106	TMT0 [N-Term]; HexNAc(2)Hex(6) [N7]	HexNAc(2)Hex(6)	103	1	2762.2500	0	0	4.07	225.4	225.4	225.4	8.42E-05	2.83E-05	2.69E-05	3.37E-04	3.30E-04	3.08E-05	2.72E-05	3.37E-04
Lysosome-associated membrane glycoprotein 1	LAMP1_HUMAN	GHTLTLNFTR	97-106	TMT0 [N-Term]; HexNAc(2)Hex(7) [N7]	HexNAc(2)Hex(7)	103	1	2924.3028	0	0	5.08	248.6	248.6	248.6	8.32E-06	5.29E-07	0	0	3.26E-05	3.08E-05	3.06E-05	3.76E-04
Microfibril-associated glycoprotein 4	MFAP4_HUMAN	RFNGSVSFFR	85-94	TMT0 [N-Term]; HexNAc(2)Hex(5) [N3]	HexNAc(2)Hex(5)	87	24	2657.1975	1	0	6.66	378.7	378.7	378.7	2.17E-07	1.36E-08	0	0	8.49E-07	5.36E-08	0	0
Microfibril-associated glycoprotein 4	MFAP4_HUMAN	RFNGSVSFFR	85-94	HexNAc(2)Hex(5) [N3]	HexNAc(2)Hex(5)	87	33	2433.0450	1	0	9.42	431.6	431.6	431.6	3.80E-10	6.08E-11	0	0	1.49E-09	2.39E-10	0	0
Microfibril-associated glycoprotein 4	MFAP4_HUMAN	RFNGSVSFFR	85-94	TMT0 [N-Term]; HexNAc(2)Hex(6) [N3]	HexNAc(2)Hex(6)	87	2	2819.2503	1	0	5.76	262.4	262.4	262.4	1.72E-06	1.27E-07	0	0	6.76E-06	4.99E-07	0	0
Microfibril-associated glycoprotein 4	MFAP4_HUMAN	RFNGSVSFFR	85-94	HexNAc(2)Hex(6) [N3]	HexNAc(2)Hex(6)	87	23	2595.0979	1	0	7.95	366.1	366.1	366.1	1.12E-08	1.08E-09	0	0	4.38E-08	4.22E-09	0	0
Microfibril-associated glycoprotein 4	MFAP4_HUMAN	RFNGSVSFFR	85-94	TMT0 [N-Term]; HexNAc(2)Hex(7) [N3]	HexNAc(2)Hex(7)	87	1	2981.3032	1	0	2.92	63.0	63.0	63.0	1.20E-03	2.78E-04	2.77E-04	3.15E-04	3.44E-03	5.33E-05	5.09E-05	3.15E-04
Microfibril-associated glycoprotein 4	MFAP4_HUMAN	RFNGSVSFFR	85-94	HexNAc(2)Hex(7) [N3]	HexNAc(2)Hex(7)	87	4	2757.1507	1	0	6.22	319.4	319.4	319.4	6.07E-07	4.22E-08	0	0	2.38E-06	1.67E-07	0	0
Microfibril-associated glycoprotein 4	MFAP4_HUMAN	RFNGSVSFFR	85-94	HexNAc(2)Hex(8) [N3]	HexNAc(2)Hex(8)	87	1	2919.2035	1	0	2.11	196.5	196.5	196.5	7.76E-03	4.57E-04	4.55E-04	6.02E-04	1.97E-02	1.45E-04	1.45E-04	1.50E-03
Microfibril-associated glycoprotein 4	MFAP4_HUMAN	FNGSVSFFR	86-94	TMT0 [N-Term]; HexNAc(2)Hex(4) [N2]	HexNAc(2)Hex(4)	87	4	2339.0436	0	0	4.91	309.5	305.2	305.2	1.24E-05	7.41E-07	0	0	4.85E-05	3.08E-05	2.99E-05	3.69E-04
Microfibril-associated glycoprotein 4	MFAP4_HUMAN	FNGSVSFFR	86-94	HexNAc(2)Hex(5) [N2]	HexNAc(2)Hex(5)	87	5	2276.9439	0	0	5.79	347.3	347.3	347.3	1.63E-06	1.20E-07	0	0	6.40E-06	4.72E-07	0	0
Microfibril-associated glycoprotein 4	MFAP4_HUMAN	FNGSVSFFR	86-94	TMT0 [N-Term]; HexNAc(2)Hex(5) [N2]	HexNAc(2)Hex(5)	87	13	2501.0964	0	0	8.76	483.1	443.3	443.3	1.72E-09	2.04E-10	0	0	6.76E-09	7.93E-10	0	0
Microfibril-associated glycoprotein 4	MFAP4_HUMAN	FNGSVSFFR	86-94	HexNAc(2)Hex(6) [N2]	HexNAc(2)Hex(6)	87	4	2438.9967	0	0	5.19	295.6	295.6	295.6	6.41E-06	4.29E-07	0	0	2.51E-05	1.69E-06	0	0
Microfibril-associated glycoprotein 4	MFAP4_HUMAN	FNGSVSFFR	86-94	TMT0 [N-Term]; HexNAc(2)Hex(6) [N2]	HexNAc(2)Hex(6)	87	16	2663.1492	0	0	7.55	414.4	386.3	386.3	2.80E-08	2.24E-09	0	0	1.10E-07	8.76E-09	0	0
Microfibril-associated glycoprotein 4	MFAP4_HUMAN	FNGSVSFFR	86-94	HexNAc(2)Hex(7) [N2]	HexNAc(2)Hex(7)	87	1	2601.0496	0	0	2.05	212.9	212.9	212.9	8.94E-03	5.45E-04	5.38E-04	5.85E-04	2.40E-02	2.36E-04	2.36E-04	2.34E-03
Microfibril-associated glycoprotein 4	MFAP4_HUMAN	FNGSVSFFR	86-94	TMT0 [N-Term]; HexNAc(2)Hex(7) [N2]	HexNAc(2)Hex(7)	87	12	2825.2020	0	0	5.36	301.3	301.3	301.3	4.35E-06	3.07E-07	0	0	1.70E-05	1.21E-06	0	0
Microfibril-associated glycoprotein 4	MFAP4_HUMAN	FNGSVSFFR	86-94	TMT0 [N-Term]; HexNAc(2)Hex(8) [N2]	HexNAc(2)Hex(8)	87	3	2987.2549	0	0	3.71	223.4	223.4	223.4	1.95E-04	5.26E-05	5.20E-05	3.25E-04	7.63E-04	5.33E-05	5.25E-05	3.25E-04
Microfibril-associated glycoprotein 4	MFAP4_HUMAN	VDLEDFENNTAYAK	130-143	TMT0 [N-Term]; HexNAc(2)Hex(10)Fuc(1)NeuAc(1) [N8]	HexNAc(2)Hex(10)Fuc(1)NeuAc(1)	137	1	4316.7366	0	1	3.09	307.6	250.5	15.1	8.11E-04	2.30E-04	2.29E-04	3.18E-04	2.44E-03	5.33E-05	5.13E-05	3.17E-04
Microfibril-associated glycoprotein 4	MFAP4_HUMAN	VDLEDFENNTAYAK	130-143	TMT0 [N-Term]; HexNAc(2)Hex(12) [N8]	HexNAc(2)Hex(12)	137	1	4203.6890	0	0	6.81	538.1	509.9	3.5	1.55E-07	9.59E-09	0	0	6.09E-07	3.77E-08	0	0
Microfibril-associated glycoprotein 4	MFAP4_HUMAN	VDLEDFENNTAYAK	130-143	TMT0 [N-Term]; HexNAc(3)Hex(11) [N8]	HexNAc(3)Hex(11)	137	2	4244.7155	0	0	6.20	439.7	405.9	3.6	6.36E-07	4.49E-08	0	0	2.50E-06	1.77E-07	0	0
Microfibril-associated glycoprotein 4	MFAP4_HUMAN	VDLEDFENNTAYAK	130-143	TMT0 [N-Term]; TMT0 [K14]; HexNAc(4)Hex(4)Fuc(1) [N8]	HexNAc(4)Hex(4)Fuc(1)	137	1	3683.6355	0	0	5.92	377.8	231.1	231.1	1.19E-06	8.77E-08	0	0	4.68E-06	3.45E-07	0	0
Microfibril-associated glycoprotein 4	MFAP4_HUMAN	VDLEDFENNTAYAK	130-143	TMT0 [N-Term]; HexNAc(4)Hex(4)Fuc(1)NeuAc(1) [N8]	HexNAc(4)Hex(4)Fuc(1)NeuAc(1)	137	8	3750.5784	0	0	9.48	490.3	458.0	458.0	3.27E-10	5.20E-11	0	0	1.28E-09	2.04E-10	0	0
Microfibril-associated glycoprotein 4	MFAP4_HUMAN	VDLEDFENNTAYAK	130-143	TMT0 [N-Term]; TMT0 [K14]; HexNAc(4)Hex(4)Fuc(1)NeuAc(1) [N8]	HexNAc(4)Hex(4)Fuc(1)NeuAc(1)	137	4	3974.7309	0	0	12.04	633.0	489.6	489.6	9.12E-13	1.34E-13	0	0	3.58E-12	5.27E-13	0	0
Microfibril-associated glycoprotein 4	MFAP4_HUMAN	VDLEDFENNTAYAK	130-143	TMT0 [N-Term]; TMT0 [K14]; HexNAc(4)Hex(5)Fuc(1) [N8]	HexNAc(4)Hex(5)Fuc(1)	137	1	3845.6883	0	0	6.99	498.1	356.8	356.8	1.03E-07	6.49E-09	0	0	4.02E-07	2.54E-08	0	0
Microfibril-associated glycoprotein 4	MFAP4_HUMAN	VDLEDFENNTAYAK	130-143	TMT0 [N-Term]; HexNAc(4)Hex(5)Fuc(1) [N8]	HexNAc(4)Hex(5)Fuc(1)	137	5	3621.5359	0	0	8.35	481.0	439.3	439.3	4.48E-09	4.80E-10	0	0	1.75E-08	1.88E-09	0	0
Microfibril-associated glycoprotein 4	MFAP4_HUMAN	VDLEDFENNTAYAK	130-143	TMT0 [N-Term]; TMT0 [K14]; Deamidated [N9]; HexNAc(4)Hex(5)Fuc(1)NeuAc(1) [N8]	HexNAc(4)Hex(5)Fuc(1)NeuAc(1)	137	3	4137.7678	0	0	6.05	447.3	322.6	3.6	8.97E-07	6.58E-08	0	0	3.52E-06	2.59E-07	0	0
Microfibril-associated glycoprotein 4	MFAP4_HUMAN	VDLEDFENNTAYAK	130-143	TMT0 [N-Term]; HexNAc(4)Hex(5)Fuc(1)NeuAc(1) [N8]	HexNAc(4)Hex(5)Fuc(1)NeuAc(1)	137	19	3912.6313	0	0	9.95	561.9	505.1	505.1	1.13E-10	1.87E-11	0	0	4.44E-10	7.36E-11	0	0
Microfibril-associated glycoprotein 4	MFAP4_HUMAN	VDLEDFENNTAYAK	130-143	TMT0 [N-Term]; TMT0 [K14]; HexNAc(4)Hex(5)Fuc(1)NeuAc(1) [N8]	HexNAc(4)Hex(5)Fuc(1)NeuAc(1)	137	11	4136.7838	0	0	12.87	674.8	590.6	126.4	1.35E-13	2.66E-14	0	0	5.30E-13	1.04E-13	0	0
Microfibril-associated glycoprotein 4	MFAP4_HUMAN	VDLEDFENNTAYAK	130-143	TMT0 [N-Term]; Deamidated [N9]; HexNAc(4)Hex(5)Fuc(1)NeuAc(2) [N8]	HexNAc(4)Hex(5)Fuc(1)NeuAc(2)	137	2	4204.7107	0	-1	5.34	351.0	351.0	11.6	4.58E-06	3.22E-07	0	0	1.79E-05	1.27E-06	0	0
Microfibril-associated glycoprotein 4	MFAP4_HUMAN	VDLEDFENNTAYAK	130-143	TMT0 [N-Term]; TMT0 [K14]; HexNAc(4)Hex(5)Fuc(1)NeuAc(2) [N8]	HexNAc(4)Hex(5)Fuc(1)NeuAc(2)	137	8	4427.8792	0	0	8.65	538.1	405.9	12.0	2.22E-09	2.51E-10	0	0	8.70E-09	9.77E-10	0	0
Microfibril-associated glycoprotein 4	MFAP4_HUMAN	VDLEDFENNTAYAK	130-143	TMT0 [N-Term]; HexNAc(4)Hex(5)Fuc(1)NeuAc(2) [N8]	HexNAc(4)Hex(5)Fuc(1)NeuAc(2)	137	9	4203.7267	0	0	8.80	551.0	495.0	4.6	1.57E-09	1.89E-10	0	0	6.15E-09	7.34E-10	0	0
Microfibril-associated glycoprotein 4	MFAP4_HUMAN	VDLEDFENNTAYAK	130-143	TMT0 [N-Term]; TMT0 [K14]; HexNAc(4)Hex(6)NeuAc(1) [N8]	HexNAc(4)Hex(6)NeuAc(1)	137	1	4152.7787	0	0	2.58	352.5	175.8	27.5	2.64E-03	3.22E-04	3.21E-04	3.10E-04	7.13E-03	7.54E-05	7.48E-05	6.20E-04
Microfibril-associated glycoprotein 4	MFAP4_HUMAN	VDLEDFENNTAYAK	130-143	TMT0 [N-Term]; Deamidated [N9]; HexNAc(4)Hex(6)NeuAc(1) [N8]	HexNAc(4)Hex(6)NeuAc(1)	137	2	3929.6102	0	0	3.65	300.0	257.0	257.0	2.26E-04	1.30E-04	1.29E-04	3.24E-04	8.85E-04	5.33E-05	5.22E-05	3.24E-04
Microfibril-associated glycoprotein 4	MFAP4_HUMAN	VDLEDFENNTAYAK	130-143	TMT0 [N-Term]; HexNAc(4)Hex(6)NeuAc(1) [N8]	HexNAc(4)Hex(6)NeuAc(1)	137	3	3928.6262	0	0	8.13	407.1	407.1	407.1	7.44E-09	7.71E-10	0	0	2.92E-08	3.02E-09	0	0
Microfibril-associated glycoprotein 4	MFAP4_HUMAN	VDLEDFENNTAYAK	130-143	TMT0 [N-Term]; HexNAc(4)Hex(6)NeuAc(2) [N8]	HexNAc(4)Hex(6)NeuAc(2)	137	1	4219.7216	0	0	4.65	275.2	231.0	3.3	2.24E-05	1.27E-06	0	0	8.76E-05	3.08E-05	2.89E-05	3.58E-04
Microfibril-associated glycoprotein 4	MFAP4_HUMAN	VDLEDFENNTAYAK	130-143	TMT0 [N-Term]; TMT0 [K14]; HexNAc(5)Hex(4)Fuc(1)NeuAc(1) [N8]	HexNAc(5)Hex(4)Fuc(1)NeuAc(1)	137	4	4177.8103	0	0	8.53	531.9	448.6	76.5	2.95E-09	3.27E-10	0	0	1.16E-08	1.28E-09	0	0
Microfibril-associated glycoprotein 4	MFAP4_HUMAN	VDLEDFENNTAYAK	130-143	TMT0 [N-Term]; HexNAc(5)Hex(4)Fuc(1)NeuAc(1) [N8]	HexNAc(5)Hex(4)Fuc(1)NeuAc(1)	137	1	3953.6578	0	0	9.83	545.8	510.1	510.1	1.49E-10	2.40E-11	0	0	5.84E-10	9.45E-11	0	0
Microfibril-associated glycoprotein 4	MFAP4_HUMAN	VDLEDFENNTAYAK	130-143	TMT0 [N-Term]; TMT0 [K14]; HexNAc(5)Hex(4)Fuc(1)NeuAc(2) [N8]	HexNAc(5)Hex(4)Fuc(1)NeuAc(2)	137	3	4468.9057	0	-1	7.42	491.1	384.9	3.9	3.77E-08	2.85E-09	0	0	1.48E-07	1.11E-08	0	0
Microfibril-associated glycoprotein 4	MFAP4_HUMAN	VDLEDFENNTAYAK	130-143	TMT0 [N-Term]; HexNAc(5)Hex(4)Fuc(1)NeuAc(2) [N8]	HexNAc(5)Hex(4)Fuc(1)NeuAc(2)	137	2	4244.7532	0	0	8.58	512.4	453.4	6.6	2.61E-09	2.91E-10	0	0	1.02E-08	1.14E-09	0	0
Microfibril-associated glycoprotein 4	MFAP4_HUMAN	VDLEDFENNTAYAK	130-143	TMT0 [N-Term]; HexNAc(5)Hex(5)Fuc(1)NeuAc(1) [N8]	HexNAc(5)Hex(5)Fuc(1)NeuAc(1)	137	2	4115.7106	0	0	6.59	393.1	371.9	99.1	2.56E-07	1.65E-08	0	0	1.00E-06	6.49E-08	0	0
Microfibril-associated glycoprotein 4	MFAP4_HUMAN	VDLEDFENNTAYAK	130-143	TMT0 [N-Term]; HexNAc(5)Hex(5)Fuc(1)NeuAc(2) [N8]	HexNAc(5)Hex(5)Fuc(1)NeuAc(2)	137	1	4406.8061	0	0	4.30	311.1	166.9	68.1	4.97E-05	2.83E-05	2.76E-05	3.45E-04	1.95E-04	3.08E-05	2.78E-05	3.44E-04
Microfibril-associated glycoprotein 4	MFAP4_HUMAN	VDLEDFENNTAYAK	130-143	TMT0 [N-Term]; Deamidated [N9]; HexNAc(5)Hex(5)NeuAc(1) [N8]	HexNAc(5)Hex(5)NeuAc(1)	137	1	3970.6368	0	0	3.91	265.8	258.2	258.2	1.22E-04	2.83E-05	2.65E-05	3.32E-04	4.79E-04	3.08E-05	2.68E-05	3.32E-04
Microfibril-associated glycoprotein 4	MFAP4_HUMAN	VDLEDFENNTAYAK	130-143	TMT0 [N-Term]; HexNAc(5)Hex(5)NeuAc(1) [N8]	HexNAc(5)Hex(5)NeuAc(1)	137	8	3969.6527	0	0	8.06	416.0	412.5	412.5	8.65E-09	8.73E-10	0	0	3.39E-08	3.42E-09	0	0
Microfibril-associated glycoprotein 4	MFAP4_HUMAN	VDLEDFENNTAYAK	130-143	TMT0 [N-Term]; HexNAc(5)Hex(5)NeuAc(2) [N8]	HexNAc(5)Hex(5)NeuAc(2)	137	2	4260.7482	0	-1	4.82	345.8	293.4	293.4	1.53E-05	9.01E-07	0	0	6.00E-05	3.08E-05	2.95E-05	3.65E-04
Microfibril-associated glycoprotein 4	MFAP4_HUMAN	VDLEDFENNTAYAK	130-143	TMT0 [N-Term]; HexNAc(5)Hex(6)Fuc(1)NeuAc(1) [N8]	HexNAc(5)Hex(6)Fuc(1)NeuAc(1)	137	6	4277.7635	0	0	5.34	343.7	216.1	64.9	4.61E-06	3.24E-07	0	0	1.81E-05	1.27E-06	0	0
Microfibril-associated glycoprotein 4	MFAP4_HUMAN	VDLEDFENNTAYAK	130-143	TMT0 [N-Term]; TMT0 [K14]; HexNAc(5)Hex(6)Fuc(1)NeuAc(2) [N8]	HexNAc(5)Hex(6)Fuc(1)NeuAc(2)	137	1	4793.0114	0	0	2.20	238.0	118.7	41.2	6.38E-03	4.36E-04	4.35E-04	6.06E-04	1.63E-02	9.77E-05	9.76E-05	9.10E-04
Microfibril-associated glycoprotein 4	MFAP4_HUMAN	VDLEDFENNTAYAK	130-143	TMT0 [N-Term]; Deamidated [N9]; HexNAc(5)Hex(6)Fuc(1)NeuAc(2) [N8]	HexNAc(5)Hex(6)Fuc(1)NeuAc(2)	137	1	4569.8429	0	-1	4.59	381.6	204.5	4.7	2.56E-05	1.41E-06	0	0	1.00E-04	3.08E-05	2.87E-05	3.55E-04
Microfibril-associated glycoprotein 4	MFAP4_HUMAN	VDLEDFENNTAYAK	130-143	TMT0 [N-Term]; HexNAc(5)Hex(6)Fuc(1)NeuAc(2) [N8]	HexNAc(5)Hex(6)Fuc(1)NeuAc(2)	137	2	4568.8589	0	0	5.26	323.5	242.0	35.4	5.48E-06	3.78E-07	0	0	2.15E-05	1.48E-06	0	0
Microfibril-associated glycoprotein 4	MFAP4_HUMAN	VDLEDFENNTAYAK	130-143	TMT0 [N-Term]; Deamidated [N9]; HexNAc(6)Hex(5)NeuAc(1) [N8]	HexNAc(6)Hex(5)NeuAc(1)	137	4	4173.7161	0	1	4.00	286.6	198.4	181.3	1.00E-04	2.83E-05	2.67E-05	3.35E-04	3.94E-04	3.08E-05	2.70E-05	3.35E-04
Microfibril-associated glycoprotein 4	MFAP4_HUMAN	VDLEDFENNTAYAK	130-143	TMT0 [N-Term]; Deamidated [N9]; HexNAc(6)Hex(6)NeuAc(2) [N8]	HexNAc(6)Hex(6)NeuAc(2)	137	1	4626.8644	0	1	2.09	286.9	132.1	132.1	8.04E-03	4.77E-04	4.76E-04	5.99E-04	2.07E-02	1.45E-04	1.44E-04	1.50E-03
Microfibril-associated glycoprotein 4	MFAP4_HUMAN	VDLEDFENNTAYAK	130-143	TMT0 [N-Term]; TMT0 [K14]; HexNAc(6)Hex(7) [N8]	HexNAc(6)Hex(7)	137	1	4429.8948	0	-1	3.87	281.4	228.7	12.6	1.36E-04	2.83E-05	2.64E-05	3.31E-04	5.32E-04	5.33E-05	5.33E-05	3.30E-04
Periostin	POSTN_HUMAN	EVNDTLLVNELK	597-608	TMT0 [N-Term]; TMT0 [K12]; HexNAc(4)Hex(5)Fuc(1)NeuAc(1) [N3]	HexNAc(4)Hex(5)Fuc(1)NeuAc(1)	599	1	3894.7874	0	0	4.35	269.1	223.3	223.3	4.47E-05	2.83E-05	2.77E-05	3.46E-04	1.75E-04	3.08E-05	2.80E-05	3.46E-04
Periostin	POSTN_HUMAN	EVNDTLLVNELK	597-608	TMT0 [N-Term]; HexNAc(4)Hex(5)Fuc(1)NeuAc(1) [N3]	HexNAc(4)Hex(5)Fuc(1)NeuAc(1)	599	1	3670.6349	0	0	4.30	230.7	230.7	230.7	4.98E-05	2.83E-05	2.76E-05	3.44E-04	1.95E-04	3.08E-05	2.78E-05	3.44E-04
Periostin	POSTN_HUMAN	EVNDTLLVNELK	597-608	TMT0 [N-Term]; HexNAc(5)Hex(4)Fuc(1)NeuAc(1) [N3]	HexNAc(5)Hex(4)Fuc(1)NeuAc(1)	599	1	3711.6615	0	0	4.58	252.5	233.4	233.4	2.61E-05	1.43E-06	0	0	1.02E-04	3.08E-05	2.87E-05	3.55E-04
Periostin	POSTN_HUMAN	EVNDTLLVNELK	597-608	TMT0 [N-Term]; TMT0 [K12]; HexNAc(5)Hex(4)Fuc(1)NeuAc(1) [N3]	HexNAc(5)Hex(4)Fuc(1)NeuAc(1)	599	1	3935.8139	0	0	2.84	246.1	155.1	155.1	1.44E-03	2.78E-04	2.76E-04	3.14E-04	4.05E-03	5.33E-05	5.07E-05	3.14E-04
Prosaposin	SAP_HUMAN	NLEKNSTK	422-429	TMT0 [N-Term]; HexNAc(2)Hex(2) [N5]	HexNAc(2)Hex(2)	426	1	1887.9169	1	0	4.81	245.8	245.8	153.6	1.56E-05	9.18E-07	0	0	6.11E-05	3.08E-05	2.95E-05	3.64E-04
Prosaposin	SAP_HUMAN	NLEKNSTK	422-429	TMT0 [K4]; HexNAc(2)Hex(2)Fuc(1) [N5]	HexNAc(2)Hex(2)Fuc(1)	426	6	2033.9748	1	0	3.95	176.7	176.7	49.1	1.11E-04	2.83E-05	2.66E-05	3.34E-04	4.36E-04	3.08E-05	2.69E-05	3.34E-04
Prosaposin	SAP_HUMAN	NLEKNSTK	422-429	TMT0 [N-Term]; HexNAc(2)Hex(2)Fuc(1) [N5]	HexNAc(2)Hex(2)Fuc(1)	426	28	2033.9748	1	0	5.27	250.7	250.7	231.6	5.41E-06	3.72E-07	0	0	2.12E-05	1.46E-06	0	0
Prosaposin	SAP_HUMAN	NLEKNSTK	422-429	TMT0 [K4]; TMT0 [K8]; HexNAc(2)Hex(2)Fuc(1) [N5]	HexNAc(2)Hex(2)Fuc(1)	426	1	2258.1272	1	0	5.44	236.3	232.1	1.5	3.66E-06	2.63E-07	0	0	1.43E-05	1.03E-06	0	0
Prosaposin	SAP_HUMAN	NLEKNSTK	422-429	TMT0 [N-Term]; TMT0 [K4]; TMT0 [K8]; HexNAc(2)Hex(2)Fuc(1) [N5]	HexNAc(2)Hex(2)Fuc(1)	426	8	2482.2797	1	0	5.59	363.1	338.6	338.6	2.55E-06	1.88E-07	0	0	1.00E-05	7.39E-07	0	0
Prosaposin	SAP_HUMAN	NLEKNSTK	422-429	TMT0 [N-Term]; TMT0 [K8]; HexNAc(2)Hex(2)Fuc(1) [N5]	HexNAc(2)Hex(2)Fuc(1)	426	11	2258.1272	1	0	7.22	391.5	391.5	240.0	6.04E-08	4.11E-09	0	0	2.37E-07	1.61E-08	0	0
Prosaposin	SAP_HUMAN	NLEKNSTK	422-429	TMT0 [N-Term]; TMT0 [K4]; TMT0 [K8]; HexNAc(2)Hex(3)Fuc(1) [N5]	HexNAc(2)Hex(3)Fuc(1)	426	3	2644.3325	1	0	5.00	285.6	285.6	285.6	9.90E-06	6.19E-07	0	0	3.88E-05	3.08E-05	3.02E-05	3.72E-04
Prosaposin	SAP_HUMAN	NLEKNSTK	422-429	TMT0 [N-Term]; HexNAc(2)Hex(3)Fuc(1) [N5]	HexNAc(2)Hex(3)Fuc(1)	426	27	2196.0276	1	0	4.83	303.8	180.9	180.9	1.47E-05	8.71E-07	0	0	5.78E-05	3.08E-05	2.96E-05	3.66E-04
Prosaposin	SAP_HUMAN	NLEKNSTK	422-429	TMT0 [K4]; TMT0 [K8]; HexNAc(2)Hex(3)Fuc(1) [N5]	HexNAc(2)Hex(3)Fuc(1)	426	1	2420.1801	1	0	4.52	209.4	209.4	209.4	3.01E-05	2.83E-05	2.82E-05	3.53E-04	1.18E-04	3.08E-05	2.85E-05	3.53E-04
Prosaposin	SAP_HUMAN	NLEKNSTK	422-429	TMT0 [K4]; HexNAc(2)Hex(3)Fuc(1) [N5]	HexNAc(2)Hex(3)Fuc(1)	426	6	2196.0276	1	0	6.09	220.5	220.5	220.5	8.11E-07	5.87E-08	0	0	3.18E-06	2.32E-07	0	0
Prosaposin	SAP_HUMAN	NLEKNSTK	422-429	TMT0 [N-Term]; TMT0 [K8]; HexNAc(2)Hex(3)Fuc(1) [N5]	HexNAc(2)Hex(3)Fuc(1)	426	1	2420.1801	1	0	6.22	430.3	370.4	256.6	5.99E-07	4.16E-08	0	0	2.35E-06	1.64E-07	0	0
Prosaposin	SAP_HUMAN	NLEKNSTK	422-429	TMT0 [N-Term]; TMT0 [K4]; HexNAc(2)Hex(4) [N5]	HexNAc(2)Hex(4)	426	1	2436.1750	1	0	4.38	244.1	244.1	43.3	4.16E-05	2.83E-05	2.78E-05	3.47E-04	1.63E-04	3.08E-05	2.80E-05	3.47E-04
Prosaposin	SAP_HUMAN	NLEKNSTK	422-429	TMT0 [K4]; HexNAc(2)Hex(4) [N5]	HexNAc(2)Hex(4)	426	3	2212.0225	1	0	3.59	165.9	165.9	49.1	2.55E-04	1.30E-04	1.29E-04	3.22E-04	1.00E-03	5.33E-05	5.20E-05	3.22E-04
Prosaposin	SAP_HUMAN	NLEKNSTK	422-429	TMT0 [N-Term]; HexNAc(2)Hex(4) [N5]	HexNAc(2)Hex(4)	426	11	2212.0225	1	0	5.33	326.0	285.5	256.1	4.72E-06	3.29E-07	0	0	1.85E-05	1.29E-06	0	0
Prosaposin	SAP_HUMAN	NLEKNSTK	422-429	TMT0 [K4]; HexNAc(2)Hex(4)Fuc(1) [N5]	HexNAc(2)Hex(4)Fuc(1)	426	1	2358.0804	1	0	2.00	94.6	94.6	47.9	9.89E-03	5.45E-04	5.31E-04	5.78E-04	2.71E-02	2.80E-04	2.80E-04	2.31E-03
Prosaposin	SAP_HUMAN	NLEKNSTK	422-429	TMT0 [N-Term]; TMT0 [K4]; HexNAc(2)Hex(4)Fuc(1) [N5]	HexNAc(2)Hex(4)Fuc(1)	426	1	2582.2329	1	0	2.10	201.5	201.5	101.4	7.94E-03	4.57E-04	4.53E-04	6.00E-04	2.04E-02	1.45E-04	1.45E-04	1.50E-03
Prosaposin	SAP_HUMAN	NLEKNSTK	422-429	TMT0 [N-Term]; TMT0 [K8]; HexNAc(2)Hex(4)Fuc(1) [N5]	HexNAc(2)Hex(4)Fuc(1)	426	1	2582.2329	1	0	4.44	260.7	260.7	127.1	3.63E-05	2.83E-05	2.80E-05	3.49E-04	1.42E-04	3.08E-05	2.82E-05	3.49E-04
Prosaposin	SAP_HUMAN	NLEKNSTK	422-429	TMT0 [N-Term]; HexNAc(2)Hex(4)Fuc(1) [N5]	HexNAc(2)Hex(4)Fuc(1)	426	10	2358.0804	1	0	4.41	241.6	241.6	241.6	3.89E-05	2.83E-05	2.79E-05	3.48E-04	1.53E-04	3.08E-05	2.81E-05	3.48E-04
Prosaposin	SAP_HUMAN	NLEKNSTK	422-429	TMT0 [N-Term]; TMT0 [K4]; TMT0 [K8]; HexNAc(2)Hex(4)Fuc(1) [N5]	HexNAc(2)Hex(4)Fuc(1)	426	1	2806.3854	1	0	3.64	239.9	239.9	239.9	2.28E-04	1.30E-04	1.29E-04	3.23E-04	8.95E-04	5.33E-05	5.22E-05	3.23E-04
Prosaposin	SAP_HUMAN	NLEKNSTK	422-429	TMT0 [K8]; HexNAc(2)Hex(4)Fuc(1) [N5]	HexNAc(2)Hex(4)Fuc(1)	426	1	2358.0804	1	0	2.95	136.3	136.3	27.0	1.13E-03	2.78E-04	2.77E-04	3.16E-04	3.27E-03	5.33E-05	5.10E-05	3.16E-04
Prosaposin	SAP_HUMAN	NLEKNSTK	422-429	TMT0 [N-Term]; HexNAc(2)Hex(5) [N5]	HexNAc(2)Hex(5)	426	10	2374.0753	1	0	4.59	287.2	265.5	265.5	2.55E-05	1.41E-06	0	0	9.99E-05	3.08E-05	2.87E-05	3.56E-04
Prosaposin	SAP_HUMAN	NLEKNSTK	422-429	TMT0 [K4]; HexNAc(2)Hex(5) [N5]	HexNAc(2)Hex(5)	426	3	2374.0753	1	0	2.74	109.9	109.9	47.9	1.84E-03	2.78E-04	2.75E-04	3.13E-04	5.07E-03	5.33E-05	5.04E-05	3.13E-04
Prosaposin	SAP_HUMAN	NLEKNSTK	422-429	TMT0 [N-Term]; HexNAc(2)Hex(5)Fuc(1) [N5]	HexNAc(2)Hex(5)Fuc(1)	426	5	2520.1332	1	0	4.05	223.3	223.3	223.3	8.87E-05	2.83E-05	2.69E-05	3.37E-04	3.48E-04	3.08E-05	2.71E-05	3.36E-04
Prosaposin	SAP_HUMAN	NLEKNSTK	422-429	TMT0 [K4]; HexNAc(2)Hex(6) [N5]	HexNAc(2)Hex(6)	426	1	2536.1282	1	0	2.04	88.2	88.2	46.7	9.06E-03	5.45E-04	5.37E-04	5.84E-04	2.45E-02	2.36E-04	2.36E-04	2.33E-03
Prosaposin	SAP_HUMAN	NLEKNSTK	422-429	TMT0 [N-Term]; HexNAc(2)Hex(6) [N5]	HexNAc(2)Hex(6)	426	2	2536.1282	1	0	4.59	227.8	227.8	227.8	2.56E-05	1.41E-06	0	0	1.01E-04	3.08E-05	2.87E-05	3.55E-04
Protein AMBP	AMBP_HUMAN	SKWNITMESYVVHTNYDEYAIFLTK	112-136	TMT0 [N-Term]; TMT0 [K2]; TMT0 [K25]; Deamidated [N15]; Oxidation [M7]; HexNAc(4)Hex(5)NeuAc(2) [N4]	HexNAc(4)Hex(5)NeuAc(2)	115	1	5946.6849	1	-1	2.79	277.7	178.1	6.3	1.61E-03	2.78E-04	2.75E-04	3.14E-04	4.49E-03	5.33E-05	5.06E-05	3.13E-04
Protein AMBP	AMBP_HUMAN	SKWNITMESYVVHTNYDEYAIFLTK	112-136	TMT0 [N-Term]; TMT0 [K2]; TMT0 [K25]; HexNAc(4)Hex(6)Fuc(1)NeuAc(1) [N4]	HexNAc(4)Hex(6)Fuc(1)NeuAc(1)	115	1	5946.7213	1	-1	2.81	305.8	175.6	14.5	1.56E-03	2.78E-04	2.76E-04	3.14E-04	4.36E-03	5.33E-05	5.06E-05	3.14E-04
Secreted frizzled-related protein 3	SFRP3_HUMAN	SLPWNMTK	45-52	TMT0 [N-Term]; TMT0 [K8]; Oxidation [M6]; HexNAc(3)Hex(11) [N5]	HexNAc(3)Hex(11)	49	1	3832.6111	0	0	2.00	232.0	125.1	125.1	9.92E-03	5.45E-04	5.31E-04	5.77E-04	2.72E-02	2.80E-04	2.80E-04	2.31E-03
Secreted frizzled-related protein 3	SFRP3_HUMAN	SLPWNMTK	45-52	TMT0 [N-Term]; TMT0 [K8]; HexNAc(4)Hex(5)Fuc(1)NeuAc(2) [N5]	HexNAc(4)Hex(5)Fuc(1)NeuAc(2)	49	5	3775.6274	0	0	4.43	294.4	205.8	23.8	3.76E-05	2.83E-05	2.79E-05	3.48E-04	1.47E-04	3.08E-05	2.82E-05	3.48E-04
Secreted frizzled-related protein 3	SFRP3_HUMAN	SLPWNMTK	45-52	TMT0 [N-Term]; TMT0 [K8]; HexNAc(4)Hex(6)NeuAc(2) [N5]	HexNAc(4)Hex(6)NeuAc(2)	49	1	3791.6223	0	0	2.10	243.6	173.1	6.7	7.93E-03	4.57E-04	4.53E-04	6.00E-04	2.03E-02	1.45E-04	1.45E-04	1.50E-03
Secreted frizzled-related protein 3	SFRP3_HUMAN	SLPWNMTK	45-52	TMT0 [N-Term]; TMT0 [K8]; HexNAc(5)Hex(4)Fuc(1)NeuAc(2) [N5]	HexNAc(5)Hex(4)Fuc(1)NeuAc(2)	49	6	3816.6539	0	0	4.52	331.3	222.8	22.3	3.02E-05	2.83E-05	2.82E-05	3.53E-04	1.18E-04	3.08E-05	2.85E-05	3.53E-04
Secreted frizzled-related protein 3	SFRP3_HUMAN	SLPWNMTK	45-52	TMT0 [N-Term]; TMT0 [K8]; HexNAc(6)Hex(3)Fuc(1)NeuAc(2) [N5]	HexNAc(6)Hex(3)Fuc(1)NeuAc(2)	49	4	3857.6805	0	0	4.49	315.3	246.0	246.0	3.27E-05	2.83E-05	2.81E-05	3.51E-04	1.28E-04	3.08E-05	2.84E-05	3.51E-04
Serotransferrin	TRFE_HUMAN	CGLVPVLAENYNK	421-433	TMT0 [N-Term]; Carbamidomethyl [C1]; HexNAc(4)Hex(5)NeuAc(2) [N12]	HexNAc(4)Hex(5)NeuAc(2)	432	7	3905.6764	0	0	6.09	356.9	356.9	356.9	8.08E-07	5.86E-08	0	0	3.17E-06	2.31E-07	0	0

Serotransferrin	TRFE_HUMAN	CGLVPVLAENYNK	421-433	TMT0 [N-Term]; TMT0 [K13]; Carbamidomethyl [C1]; HexNAc(4)Hex(5)NeuAc(2) [N12]	HexNAc(4)Hex(5)NeuAc(2)	432	15	4129.8289	0	0	8.49	492.8	451.1	97.4	3.23E-09	3.54E-10	0	0	1.27E-08	1.38E-09	0	0
Tubulointerstitial nephritis antigen-like	TINAL_HUMAN	AINQGNYGWQAGNHSAFWGMTLDEG	149-175	TMT0 [N-Term]; Oxidation [M20]; HexNAc(2)Hex(5) [N13]	HexNAc(2)Hex(5)	161	1	4449.9450	0	0	4.43	283.4	175.9	76.5	3.73E-05	2.83E-05	2.79E-05	3.49E-04	1.46E-04	3.08E-05	2.82E-05	3.49E-04
Versican core protein	CSPG2_HUMAN	FENQTGFPPPDSR	328-340	TMT0 [N-Term]; HexNAc(4)Hex(5)Fuc(1)NeuAc(1) [N3]	HexNAc(4)Hex(5)Fuc(1)NeuAc(1)	330	39	3775.5737	0	0	6.31	351.2	329.2	329.2	4.94E-07	3.43E-08	0	0	1.94E-06	1.35E-07	0	0
Versican core protein	CSPG2_HUMAN	FENQTGFPPPDSR	328-340	TMT0 [N-Term]; HexNAc(4)Hex(5)Fuc(1)NeuAc(2) [N3]	HexNAc(4)Hex(5)Fuc(1)NeuAc(2)	330	9	4066.6691	0	0	5.16	276.6	218.6	0.7	6.97E-06	4.59E-07	0	0	2.73E-05	1.80E-06	0	0
Versican core protein	CSPG2_HUMAN	GQFESVAPSQNFSDSSESDTHPFVIAK	1432-1458	TMT0 [N-Term]; HexNAc(4)Hex(5)Fuc(1)NeuAc(2) [N11]	HexNAc(4)Hex(5)Fuc(1)NeuAc(2)	1442	1	5486.3209	0	0	2.09	201.3	166.0	5.0	8.04E-03	4.77E-04	4.76E-04	5.99E-04	2.08E-02	1.45E-04	1.44E-04	1.50E-03
Versican core protein	CSPG2_HUMAN	VVAENITQTSR	1894-1904	TMT0 [N-Term]; HexNAc(5)Hex(6)Fuc(1)NeuAc(1) [N5]	HexNAc(5)Hex(6)Fuc(1)NeuAc(1)	1898	1	3866.6681	0	0	6.37	465.1	373.6	373.6	4.26E-07	2.88E-08	0	0	1.67E-06	1.14E-07	0	0
Versican core protein	CSPG2_HUMAN	VVAENITQTSR	1894-1904	TMT0 [N-Term]; HexNAc(5)Hex(6)Fuc(1)NeuAc(2) [N5]	HexNAc(5)Hex(6)Fuc(1)NeuAc(2)	1898	2	4157.7635	0	0	5.53	420.0	273.7	87.7	2.98E-06	2.17E-07	0	0	1.17E-05	8.53E-07	0	0
Versican core protein	CSPG2_HUMAN	VVAENITQTSR	1894-1904	TMT0 [N-Term]; HexNAc(5)Hex(6)Fuc(1)NeuAc(3) [N5]	HexNAc(5)Hex(6)Fuc(1)NeuAc(3)	1898	1	4448.8589	0	-1	3.77	290.5	290.5	290.5	1.72E-04	5.26E-05	5.22E-05	3.27E-04	6.73E-04	5.33E-05	5.27E-05	3.27E-04
Vitronectin	VTNC_HUMAN	NGSLFAFR	169-176	TMT0 [N-Term]; HexNAc(3)Hex(4)NeuAc(1) [N1]	HexNAc(3)Hex(4)NeuAc(1)	169	1	2684.1707	0	0	4.29	244.5	244.5	244.5	5.17E-05	2.83E-05	2.75E-05	3.44E-04	2.03E-04	3.08E-05	2.78E-05	3.44E-04
Vitronectin	VTNC_HUMAN	NGSLFAFR	169-176	HexNAc(3)Hex(5)NeuAc(1) [N1]	HexNAc(3)Hex(5)NeuAc(1)	169	1	2622.0710	0	0	4.05	235.0	235.0	235.0	9.00E-05	2.83E-05	2.68E-05	3.36E-04	3.53E-04	3.08E-05	2.71E-05	3.36E-04

Supplemental Table IV. Quantification of Glycopeptides

Protein Name	UniProt ID	Peptide	Glycan composition	Start	End	Glycosylation site	Control Mean (Log2 intensity)	Control SD (Log2 intensity)	MFS Mean (Log2 intensity)	MFS SD (Log2 intensity)	Log2 Fold Change (MFS/ Control)	Adjusted P-value *
Beta-2-glycoprotein 1	APOH_HUMAN	[TMT0]VYK[TMT0]PSAGN[dHex1Hex5HexNAc4NeuAc2]N[Deamid]SLYR	dHex1Hex5HexNAc4NeuAc2	155	167	162	12.96	9.58	20.23	1.67	7.28	0.0386
Beta-2-glycoprotein 1	APOH_HUMAN	[TMT0]VYK[TMT0]PSAGN[dHex1Hex5HexNAc4NeuAc2]NSLYR	dHex1Hex5HexNAc4NeuAc2	155	167	162	13.79	10.22	21.44	1.79	7.65	0.0398
Beta-2-glycoprotein 1	APOH_HUMAN	[TMT0]VYK[TMT0]PSAGN[Hex5HexNAc4NeuAc2]N[Deamid]SLYR	Hex5HexNAc4NeuAc2	155	167	162	19.36	3.69	22.64	1.09	3.29	0.0089
Beta-2-glycoprotein 1	APOH_HUMAN	[TMT0]VYK[TMT0]PSAGN[Hex5HexNAc4NeuAc2]NSLYR	Hex5HexNAc4NeuAc2	155	167	162	21.16	3.12	23.58	1.33	2.42	0.0228
Beta-2-glycoprotein 1	APOH_HUMAN	[TMT0]VYKPSAGN[dHex1Hex5HexNAc4NeuAc2]NSLYR	dHex1Hex5HexNAc4NeuAc2	155	167	162	15.19	9.26	21.15	1.39	5.97	0.0293
Beta-2-glycoprotein 1	APOH_HUMAN	[TMT0]VYKPSAGN[Hex5HexNAc4NeuAc2]N[Deamid]SLYR	Hex5HexNAc4NeuAc2	155	167	162	19.92	2.42	22.18	0.98	2.26	0.0127
Beta-2-glycoprotein 1	APOH_HUMAN	[TMT0]VYKPSAGN[Hex5HexNAc4NeuAc2]NSLYR	Hex5HexNAc4NeuAc2	155	167	162	18.18	8.72	23.32	0.94	5.14	0.0276
Beta-2-glycoprotein 1	APOH_HUMAN	[TMT0]LGN[Hex5HexNAc4NeuAc1]WSAM[Oxid]PSC[Carbamidomethyl]K[TMT0]	Hex5HexNAc4NeuAc1	251	261	253	12.58	7.64	18.91	1.30	6.32	0.0008
Beta-2-glycoprotein 1	APOH_HUMAN	[TMT0]LGN[Hex5HexNAc4NeuAc1]WSAMPSC[Carbamidomethyl]K[TMT0]	Hex5HexNAc4NeuAc1	251	261	253	17.35	1.88	19.78	0.68	2.42	0.0011
Beta-2-glycoprotein 1	APOH_HUMAN	[TMT0]LGN[Hex5HexNAc4NeuAc2]WSAM[Oxid]PSC[Carbamidomethyl]K	Hex5HexNAc4NeuAc2	251	261	253	15.02	7.37	20.73	0.99	5.71	0.0052
Beta-2-glycoprotein 1	APOH_HUMAN	[TMT0]LGN[Hex5HexNAc4NeuAc2]WSAM[Oxid]PSC[Carbamidomethyl]K[TMT0]	Hex5HexNAc4NeuAc2	251	261	253	20.65	1.77	22.70	0.88	2.05	0.0064
Beta-2-glycoprotein 1	APOH_HUMAN	[TMT0]LGN[Hex5HexNAc4NeuAc2]WSAMPSC[Carbamidomethyl]K	Hex5HexNAc4NeuAc2	251	261	253	18.45	6.26	21.28	0.78	2.83	0.2309
Beta-2-glycoprotein 1	APOH_HUMAN	[TMT0]LGN[Hex5HexNAc4NeuAc2]WSAMPSC[Carbamidomethyl]K[TMT0]	Hex5HexNAc4NeuAc2	251	261	253	22.04	1.18	23.26	0.90	1.22	0.0130
Cathepsin D	CATD_HUMAN	[TMT0]GSLSYLN[Hex6HexNAc2]VTR	Hex6HexNAc2	257	266	263	17.51	5.93	19.28	1.46	1.78	0.8000
Complement factor H	CFAH_HUMAN	[TMT0]IPC[Carbamidomethyl]SQPPQIEHGTIN[Hex5HexNAc4NeuAc2]SSR	Hex5HexNAc4NeuAc2	868	885	882	21.57	1.91	23.70	1.79	2.13	0.0095
Complement factor H	CFAH_HUMAN	[TMT0]ISEEN[Hex5HexNAc4NeuAc2]ETTC[Carbamidomethyl]YMGK[TMT0]	Hex5HexNAc4NeuAc2	907	919	911	18.25	2.03	21.69	1.05	3.43	0.0005
Clusterin	CLUS_HUMAN	[TMT0]LAN[dHex1Hex5HexNAc4NeuAc2]LTQGEDQYYLR	dHex1Hex5HexNAc4NeuAc2	372	385	374	16.43	8.31	19.15	1.79	2.71	0.9295
Clusterin	CLUS_HUMAN	[TMT0]LAN[Hex5HexNAc4NeuAc2]LTQGEDQYYLR	Hex5HexNAc4NeuAc2	372	385	374	11.63	8.72	15.81	5.33	4.18	0.3659
Collagen alpha-1(XVIII) chain	COIA1_HUMAN	[TMT0]FGVN[dHex1Hex5HexNAc4NeuAc1]SSDVPGPAGLP[Hydroxyl]GVP[Hydroxyl]GR	dHex1Hex5HexNAc4NeuAc1	923	942	926	10.76	9.72	18.84	1.23	8.08	0.2210
Collagen alpha-1(XVIII) chain	COIA1_HUMAN	[TMT0]FGVN[dHex1Hex5HexNAc4NeuAc2]SSDVPGPAGLP[Hydroxyl]GVP[Hydroxyl]GR	dHex1Hex5HexNAc4NeuAc2	923	942	926	18.68	2.60	20.53	0.85	1.85	0.1181
Collagen alpha-1(XVIII) chain	COIA1_HUMAN	[TMT0]FGVN[Hex6HexNAc4NeuAc1]SSDVPGPAGLPGVP[Hydroxyl]GR	Hex6HexNAc4NeuAc1	923	942	926	13.29	8.38	18.83	1.22	5.53	0.2309
Collagen alpha-1(XVIII) chain	COIA1_HUMAN	[TMT0]FGVN[Hex6HexNAc4NeuAc2]SSDVPGPAGLPGVP[Hydroxyl]GR	Hex6HexNAc4NeuAc2	923	942	926	18.78	2.36	20.54	0.86	1.76	0.0854
**Versican core protein**	**CSPG2_HUMAN**	**[TMT0]FEN[dHex1Hex5HexNAc4NeuAc1]QTGFPPPDSR**	**dHex1Hex5HexNAc4NeuAc1**	**328**	**340**	**330**	**23.51**	**2.01**	**25.62**	**0.84**	**2.10**	**0.0052**
**Versican core protein**	**CSPG2_HUMAN**	**[TMT0]FEN[dHex1Hex5HexNAc4NeuAc2]QTGFPPPDSR**	**dHex1Hex5HexNAc4NeuAc2**	**328**	**340**	**330**	**22.69**	**2.53**	**25.17**	**0.67**	**2.47**	**0.0026**
Versican core protein	CSPG2_HUMAN	[TMT0]VVAEN[dHex1Hex6HexNAc5NeuAc1]ITQTSR	dHex1Hex6HexNAc5NeuAc1	1894	1904	1898	18.10	6.25	20.25	1.28	2.15	0.9650
Versican core protein	CSPG2_HUMAN	[TMT0]VVAEN[dHex1Hex6HexNAc5NeuAc2]ITQTSR	dHex1Hex6HexNAc5NeuAc2	1894	1904	1898	18.42	8.67	22.26	0.70	3.84	0.4870
Versican core protein	CSPG2_HUMAN	[TMT0]VVAEN[dHex1Hex6HexNAc5NeuAc3]ITQTSR	dHex1Hex6HexNAc5NeuAc3	1894	1904	1898	18.78	6.20	21.39	0.68	2.62	0.2210
Alpha-2-HS-glycoprotein	FETUA_HUMAN	[TMT0]VC[Carbamidomethyl]QDC[Carbamidomethyl]PLLAPLN[Hex5HexNAc4NeuAc2]DTR	Hex5HexNAc4NeuAc2	145	159	156	20.33	1.44	21.53	0.88	1.20	0.0386
Alpha-2-HS-glycoprotein	FETUA_HUMAN	[TMT0]AALAAFNAQNN[Hex5HexNAc4NeuAc2]GSNFQLEEISR	Hex5HexNAc4NeuAc2	166	187	176	14.68	9.25	5.85	6.67	-8.83	0.0159
Fibromodulin	FMOD_HUMAN	[TMT0]LYLDHNN[dHex1Hex4HexNAc5NeuAc2]LTR	dHex1Hex4HexNAc5NeuAc2	160	169	166	19.62	2.41	20.95	1.41	1.33	0.2092
Fibromodulin	FMOD_HUMAN	[TMT0]LYLDHNN[dHex1Hex5HexNAc4NeuAc1]LTR	dHex1Hex5HexNAc4NeuAc1	160	169	166	24.01	0.95	23.64	0.74	-0.37	0.2210
Fibromodulin	FMOD_HUMAN	[TMT0]LYLDHNN[dHex1Hex5HexNAc4NeuAc2]LTR	dHex1Hex5HexNAc4NeuAc2	160	169	166	21.55	1.45	22.52	0.89	0.98	0.0994
Hemopexin	HEMO_HUMAN	[TMT0]SWPAVGN[dHex1Hex5HexNAc4NeuAc2]C[Carbamidomethyl]SSALR	dHex1Hex5HexNAc4NeuAc2	181	193	187	19.26	1.45	19.54	1.18	0.28	0.5669
Hemopexin	HEMO_HUMAN	[TMT0]SWPAVGN[Hex5HexNAc4NeuAc2]C[Carbamidomethyl]SSALR	Hex5HexNAc4NeuAc2	181	193	187	22.62	1.01	23.14	0.67	0.52	0.3084
Hemopexin	HEMO_HUMAN	[TMT0]ALPQPQN[Hex5HexNAc4NeuAc2]VTSLLGC[Carbamidomethyl]TH	Hex5HexNAc4NeuAc2	447	462	453	18.91	2.30	20.72	1.44	1.82	0.0743
Hyaluronan and proteoglycan link protein 1	HPLN1_HUMAN	[TMT0]GGN[dHex1Hex4HexNAc5NeuAc2]VTLPC[Carbamidomethyl]K[TMT0]	dHex1Hex4HexNAc5NeuAc2	54	62	56	19.54	1.65	20.32	1.94	0.78	0.3631
Hyaluronan and proteoglycan link protein 1	HPLN1_HUMAN	[TMT0]GGN[dHex1Hex5HexNAc4NeuAc1]VTLPC[Carbamidomethyl]K[TMT0]	dHex1Hex5HexNAc4NeuAc1	54	62	56	17.23	8.19	20.05	2.83	2.83	0.4797
Hyaluronan and proteoglycan link protein 1	HPLN1_HUMAN	[TMT0]GGN[dHex1Hex5HexNAc4NeuAc2]VTLPC[Carbamidomethyl]K[TMT0]	dHex1Hex5HexNAc4NeuAc2	54	62	56	20.48	2.04	21.38	2.21	0.89	0.2967
Haptoglobin	HPT_HUMAN	[TMT0]VVLHPN[dHex1Hex6HexNAc4NeuAc1]YSQVDIGLIK	dHex1Hex6HexNAc4NeuAc1	236	251	241	8.65	7.65	8.96	7.85	0.31	0.8000
Haptoglobin	HPT_HUMAN	[TMT0]VVLHPN[Hex5HexNAc4NeuAc2]YSQVDIGLIK	Hex5HexNAc4NeuAc2	236	251	241	3.95	7.20	2.52	6.72	-1.44	0.6841
Haptoglobin	HPT_HUMAN	[TMT0]VVLHPN[Hex6HexNAc5NeuAc3]YSQVDIGLIK	Hex6HexNAc5NeuAc3	236	251	241	6.30	7.90	8.88	7.26	2.58	0.5692
Ig gamma-1 chain C region	IGHG1_HUMAN	[TMT0]EEQYN[dHex1Hex3HexNAc4]STYR	dHex1Hex3HexNAc4	176	184	180	22.65	2.43	24.05	1.64	1.40	0.2210
Ig gamma-1 chain C region	IGHG1_HUMAN	[TMT0]EEQYN[Hex3HexNAc4]STYR	Hex3HexNAc4	176	184	180	19.77	2.50	20.71	1.58	0.94	0.1637
Ig gamma-1 chain C region	IGHG1_HUMAN	[TMT0]EEQYN[Hex4HexNAc4]STYR	Hex4HexNAc4	176	184	180	22.46	1.69	23.58	0.73	1.12	0.0499
Ig gamma-1 chain C region	IGHG1_HUMAN	[TMT0]EEQYN[Hex5HexNAc4]STYR	Hex5HexNAc4	176	184	180	20.40	3.07	22.53	0.80	2.13	0.1717
Ig gamma-1 chain C region	IGHG1_HUMAN	[TMT0]TK[TMT0]PREEQYN[dHex1Hex4HexNAc4]STYR	dHex1Hex4HexNAc4	172	184	180	16.22	5.49	18.41	4.86	2.19	0.1035
Ig gamma-1 chain C region	IGHG1_HUMAN	[TMT0]TKPREEQYN[dHex1Hex3HexNAc4]STYR	dHex1Hex3HexNAc4	172	184	180	13.92	8.54	17.56	6.07	3.65	0.2909
Ig gamma-1 chain C region	IGHG1_HUMAN	[TMT0]TKPREEQYN[dHex1Hex4HexNAc4]STYR	dHex1Hex4HexNAc4	172	184	180	20.51	1.19	20.54	1.64	0.03	0.7778
Ig gamma-1 chain C region	IGHG1_HUMAN	[TMT0]TKPREEQYN[dHex1Hex5HexNAc4]STYR	dHex1Hex5HexNAc4	172	184	180	7.55	9.45	16.93	5.89	9.37	0.0234
Ig gamma-2 chain C region	IGHG2_HUMAN	[TMT0]EEQFN[dHex1Hex3HexNAc4]STFR	dHex1Hex3HexNAc4	172	180	176	24.12	1.05	25.48	0.66	1.36	0.0046
Ig gamma-2 chain C region	IGHG2_HUMAN	[TMT0]EEQFN[dHex1Hex4HexNAc4NeuAc1]STFR	dHex1Hex4HexNAc4NeuAc1	172	180	176	22.07	0.91	23.96	0.66	1.89	0.0005
Ig gamma-2 chain C region	IGHG2_HUMAN	[TMT0]EEQFN[dHex1Hex5HexNAc4]STFR	dHex1Hex5HexNAc4	172	180	176	22.61	1.14	24.95	0.80	2.34	0.0005
Ig gamma-2 chain C region	IGHG2_HUMAN	[TMT0]EEQFN[dHex1Hex7HexNAc6]STFR	dHex1Hex7HexNAc6	172	180	176	21.23	0.90	22.24	1.21	1.01	0.0320
Ig gamma-2 chain C region	IGHG2_HUMAN	[TMT0]TK[TMT0]PREEQFN[dHex1Hex3HexNAc4]STFR	dHex1Hex3HexNAc4	168	180	176	6.73	8.33	19.22	4.56	12.49	0.0005
Ig gamma-2 chain C region	IGHG2_HUMAN	[TMT0]TKPREEQFN[dHex1Hex3HexNAc4]STFR	dHex1Hex3HexNAc4	168	180	176	16.34	5.49	19.88	2.31	3.54	0.0123
Ig gamma-2 chain C region	IGHG2_HUMAN	[TMT0]TKPREEQFN[dHex1Hex4HexNAc4]STFR	dHex1Hex4HexNAc4	168	180	176	10.11	9.12	16.30	8.17	6.19	0.0402
Ig gamma-4 chain C region	IGHG4_HUMAN	[TMT0]TK[TMT0]PREEQFN[Hex5HexNAc4]STYR	Hex5HexNAc4	169	181	177	16.34	5.49	19.50	2.18	3.16	0.0402
Ig gamma-4 chain C region	IGHG4_HUMAN	[TMT0]TKPREEQFN[Hex4HexNAc4]STYR	Hex4HexNAc4	169	181	177	15.20	7.34	17.52	6.08	2.32	0.3659
Ig gamma-4 chain C region	IGHG4_HUMAN	[TMT0]TKPREEQFN[Hex5HexNAc4]STYR	Hex5HexNAc4	169	181	177	14.36	8.83	20.39	1.89	6.03	0.0228
Lysosome-associated membrane glycoprotein 1	LAMP1_HUMAN	[TMT0]GHTLTLN[Hex5HexNAc2]FTR	Hex5HexNAc2	97	106	103	19.76	2.06	19.47	0.63	-0.30	0.1677
Microfibril-associated glycoprotein 4	MFAP4_HUMAN	[TMT0]FN[Hex4HexNAc2]GSVSFFR	Hex4HexNAc2	86	94	87	15.58	5.15	17.49	4.04	1.92	0.0538
Microfibril-associated glycoprotein 4	MFAP4_HUMAN	[TMT0]FN[Hex5HexNAc2]GSVSFFR	Hex5HexNAc2	86	94	87	21.78	1.50	22.66	1.02	0.88	0.0764
**Microfibril-associated glycoprotein 4**	**MFAP4_HUMAN**	**[TMT0]FN[Hex6HexNAc2]GSVSFFR**	**Hex6HexNAc2**	**86**	**94**	**87**	**21.35**	**1.09**	**22.40**	**0.60**	**1.05**	**0.0159**
**Microfibril-associated glycoprotein 4**	**MFAP4_HUMAN**	**[TMT0]FN[Hex7HexNAc2]GSVSFFR**	**Hex7HexNAc2**	**86**	**94**	**87**	**20.44**	**0.99**	**21.86**	**0.56**	**1.42**	**0.0025**
**Microfibril-associated glycoprotein 4**	**MFAP4_HUMAN**	**[TMT0]FN[Hex8HexNAc2]GSVSFFR**	**Hex8HexNAc2**	**86**	**94**	**87**	**20.02**	**1.21**	**21.66**	**0.66**	**1.64**	**0.0016**
Microfibril-associated glycoprotein 4	MFAP4_HUMAN	[TMT0]RFN[Hex5HexNAc2]GSVSFFR	Hex5HexNAc2	85	94	87	21.41	2.23	22.52	1.20	1.11	0.2359
Microfibril-associated glycoprotein 4	MFAP4_HUMAN	[TMT0]RFN[Hex6HexNAc2]GSVSFFR	Hex6HexNAc2	85	94	87	23.04	0.94	23.24	0.66	0.20	0.5669
**Microfibril-associated glycoprotein 4**	**MFAP4_HUMAN**	**[TMT0]RFN[Hex7HexNAc2]GSVSFFR**	**Hex7HexNAc2**	**85**	**94**	**87**	**20.19**	**0.94**	**21.39**	**0.74**	**1.20**	**0.0036**
**Microfibril-associated glycoprotein 4**	**MFAP4_HUMAN**	**[TMT0]VDLEDFEN[dHex1Hex4HexNAc4NeuAc1]NTAYAK**	**dHex1Hex4HexNAc4NeuAc1**	**130**	**143**	**137**	**16.37**	**5.51**	**20.65**	**1.42**	**4.29**	**0.0025**
**Microfibril-associated glycoprotein 4**	**MFAP4_HUMAN**	**[TMT0]VDLEDFEN[dHex1Hex4HexNAc4NeuAc1]NTAYAK[TMT0]**	**dHex1Hex4HexNAc4NeuAc1**	**130**	**143**	**137**	**12.53**	**7.64**	**17.02**	**5.85**	**4.49**	**0.0204**
**Microfibril-associated glycoprotein 4**	**MFAP4_HUMAN**	**[TMT0]VDLEDFEN[dHex1Hex4HexNAc5NeuAc1]NTAYAK**	**dHex1Hex4HexNAc5NeuAc1**	**130**	**143**	**137**	**16.60**	**7.89**	**21.21**	**1.57**	**4.61**	**0.0478**

**Microfibril-associated glycoprotein 4**	**MFAP4_HUMAN**	**[TMT0]VDLEDFEN[dHex1Hex4HexNAc5NeuAc1]NTAYAK[TMT0]**	**dHex1Hex4HexNAc5NeuAc1**	**130**	**143**	**137**	**15.96**	**5.61**	**18.57**	**4.48**	**2.61**	**0.0499**
**Microfibril-associated glycoprotein 4**	**MFAP4_HUMAN**	**[TMT0]VDLEDFEN[dHex1Hex4HexNAc5NeuAc2]NTAYAK**	**dHex1Hex4HexNAc5NeuAc2**	**130**	**143**	**137**	**15.45**	**7.51**	**19.62**	**4.54**	**4.18**	**0.0293**
Microfibril-associated glycoprotein 4	MFAP4_HUMAN	[TMT0]VDLEDFEN[dHex1Hex4HexNAc5NeuAc2]NTAYAK[TMT0]	dHex1Hex4HexNAc5NeuAc2	130	143	137	14.48	6.93	17.39	4.17	2.91	0.1560
**Microfibril-associated glycoprotein 4**	**MFAP4_HUMAN**	**[TMT0]VDLEDFEN[dHex1Hex5HexNAc4]NTAYAK**	**dHex1Hex5HexNAc4**	**130**	**143**	**137**	**15.22**	**7.27**	**19.71**	**0.86**	**4.49**	**0.0120**
Microfibril-associated glycoprotein 4	MFAP4_HUMAN	[TMT0]VDLEDFEN[dHex1Hex5HexNAc4]NTAYAK[TMT0]	dHex1Hex5HexNAc4	130	143	137	14.24	6.80	17.20	4.09	2.96	0.1007
Microfibril-associated glycoprotein 4	MFAP4_HUMAN	[TMT0]VDLEDFEN[dHex1Hex5HexNAc4NeuAc1]N[Deamid]TAYAK[TMT0]	dHex1Hex5HexNAc4NeuAc1	130	143	137	9.60	8.56	15.13	6.39	5.53	0.0587
**Microfibril-associated glycoprotein 4**	**MFAP4_HUMAN**	**[TMT0]VDLEDFEN[dHex1Hex5HexNAc4NeuAc1]NTAYAK**	**dHex1Hex5HexNAc4NeuAc1**	**130**	**143**	**137**	**22.52**	**2.25**	**24.41**	**0.69**	**1.89**	**0.0095**
**Microfibril-associated glycoprotein 4**	**MFAP4_HUMAN**	**[TMT0]VDLEDFEN[dHex1Hex5HexNAc4NeuAc1]NTAYAK[TMT0]**	**dHex1Hex5HexNAc4NeuAc1**	**130**	**143**	**137**	**20.25**	**2.57**	**22.43**	**1.08**	**2.17**	**0.0320**
**Microfibril-associated glycoprotein 4**	**MFAP4_HUMAN**	**[TMT0]VDLEDFEN[dHex1Hex5HexNAc4NeuAc2]NTAYAK**	**dHex1Hex5HexNAc4NeuAc2**	**130**	**143**	**137**	**21.05**	**2.95**	**23.53**	**0.60**	**2.48**	**0.0076**
**Microfibril-associated glycoprotein 4**	**MFAP4_HUMAN**	**[TMT0]VDLEDFEN[dHex1Hex5HexNAc4NeuAc2]NTAYAK[TMT0]**	**dHex1Hex5HexNAc4NeuAc2**	**130**	**143**	**137**	**17.67**	**5.88**	**21.12**	**1.09**	**3.45**	**0.0110**
**Microfibril-associated glycoprotein 4**	**MFAP4_HUMAN**	**[TMT0]VDLEDFEN[dHex1Hex5HexNAc5NeuAc1]NTAYAK**	**dHex1Hex5HexNAc5NeuAc1**	**130**	**143**	**137**	**9.74**	**8.62**	**17.48**	**4.40**	**7.74**	**0.0080**
Microfibril-associated glycoprotein 4	MFAP4_HUMAN	[TMT0]VDLEDFEN[dHex1Hex6HexNAc5NeuAc1]NTAYAK	dHex1Hex6HexNAc5NeuAc1	130	143	137	17.78	5.89	20.54	0.97	2.76	0.0786
**Microfibril-associated glycoprotein 4**	**MFAP4_HUMAN**	**[TMT0]VDLEDFEN[dHex1Hex6HexNAc5NeuAc2]NTAYAK**	**dHex1Hex6HexNAc5NeuAc2**	**130**	**143**	**137**	**19.83**	**6.45**	**22.76**	**0.88**	**2.93**	**0.0320**
Microfibril-associated glycoprotein 4	MFAP4_HUMAN	[TMT0]VDLEDFEN[dHex1Hex6HexNAc5NeuAc2]NTAYAK[TMT0]	dHex1Hex6HexNAc5NeuAc2	130	143	137	17.39	5.86	20.18	1.53	2.79	0.0796
**Microfibril-associated glycoprotein 4**	**MFAP4_HUMAN**	**[TMT0]VDLEDFEN[Hex12HexNAc2]NTAYAK**	**Hex12HexNAc2**	**130**	**143**	**137**	**21.09**	**2.74**	**23.52**	**0.60**	**2.43**	**0.0076**
**Microfibril-associated glycoprotein 4**	**MFAP4_HUMAN**	**[TMT0]VDLEDFEN[Hex5HexNAc5NeuAc1]NTAYAK**	**Hex5HexNAc5NeuAc1**	**130**	**143**	**137**	**12.17**	**9.10**	**20.06**	**0.92**	**7.89**	**0.0032**
**Microfibril-associated glycoprotein 4**	**MFAP4_HUMAN**	**[TMT0]VDLEDFEN[Hex6HexNAc4NeuAc1]N[Deamid]TAYAK**	**Hex6HexNAc4NeuAc1**	**130**	**143**	**137**	**15.24**	**7.56**	**20.65**	**0.79**	**5.41**	**0.0080**
**Microfibril-associated glycoprotein 4**	**MFAP4_HUMAN**	**[TMT0]VDLEDFEN[Hex6HexNAc4NeuAc1]NTAYAK**	**Hex6HexNAc4NeuAc1**	**130**	**143**	**137**	**18.32**	**6.10**	**21.74**	**0.61**	**3.42**	**0.0076**
**Microfibril-associated glycoprotein 4**	**MFAP4_HUMAN**	**[TMT0]VDLEDFEN[Hex6HexNAc4NeuAc1]NTAYAK[TMT0]**	**Hex6HexNAc4NeuAc1**	**130**	**143**	**137**	**17.80**	**5.85**	**20.86**	**1.04**	**3.06**	**0.0383**
**Microfibril-associated glycoprotein 4**	**MFAP4_HUMAN**	**[TMT0]VDLEDFEN[Hex6HexNAc4NeuAc2]NTAYAK**	**Hex6HexNAc4NeuAc2**	**130**	**143**	**137**	**15.66**	**7.45**	**20.39**	**0.68**	**4.73**	**0.0080**
Basement membrane-specific heparan sulfate proteoglycan core protein	PGBM_HUMAN	[TMT0]ALVN[Hex4HexNAc3NeuAc1]FTR	Hex4HexNAc3NeuAc1	86	92	89	19.06	1.21	19.68	4.66	0.62	0.0089
Basement membrane-specific heparan sulfate proteoglycan core protein	PGBM_HUMAN	[TMT0]ALVN[Hex5HexNAc4NeuAc1]FTR	Hex5HexNAc4NeuAc1	86	92	89	23.75	1.31	24.67	0.51	0.92	0.1260
Basement membrane-specific heparan sulfate proteoglycan core protein	PGBM_HUMAN	[TMT0]ALVN[Hex5HexNAc4NeuAc2]FTR	Hex5HexNAc4NeuAc2	86	92	89	25.32	1.65	26.43	0.95	1.11	0.0605
**Basement membrane-specific heparan sulfate proteoglycan core protein**	**PGBM_HUMAN**	**[TMT0]ALVN[Hex6HexNAc5NeuAc2]FTR**	**Hex6HexNAc5NeuAc2**	**86**	**92**	**89**	**17.79**	**5.94**	**20.98**	**0.56**	**3.19**	**0.0276**
Basement membrane-specific heparan sulfate proteoglycan core protein	PGBM_HUMAN	[TMT0]ALVN[Hex6HexNAc5NeuAc3]FTR	Hex6HexNAc5NeuAc3	86	92	89	20.17	1.79	21.53	0.57	1.37	0.0796
Basement membrane-specific heparan sulfate proteoglycan core protein	PGBM_HUMAN	[TMT0]SLTQGSLIVGDLAPVN[dHex1Hex5HexNAc4NeuAc1]GTSQGK	dHex1Hex5HexNAc4NeuAc1	3765	3786	3780	18.01	6.37	20.87	0.94	2.87	0.4150
**Aggrecan core protein**	**PGCA_HUMAN**	**[TMT0]SN[Hex5HexNAc2]DSGVYR**	**Hex5HexNAc2**	**125**	**132**	**126**	**17.53**	**5.89**	**20.84**	**1.67**	**3.31**	**0.0159**
**Aggrecan core protein**	**PGCA_HUMAN**	**[TMT0]SN[Hex6HexNAc2]DSGVYR**	**Hex6HexNAc2**	**125**	**132**	**126**	**19.45**	**1.37**	**20.78**	**0.84**	**1.33**	**0.0076**
Aggrecan core protein	PGCA_HUMAN	[TMT0]TVYVHAN[dHex1Hex5HexNAc4NeuAc1]QTGYPDPSSR	dHex1Hex5HexNAc4NeuAc1	327	343	333	17.35	8.13	21.07	1.75	3.71	0.1007
Aggrecan core protein	PGCA_HUMAN	[TMT0]TVYVHAN[dHex1Hex5HexNAc4NeuAc2]QTGYPDPSSR	dHex1Hex5HexNAc4NeuAc2	327	343	333	17.73	5.78	20.49	1.46	2.76	0.0545
**Aggrecan core protein**	**PGCA_HUMAN**	**[TMT0]TVYVHAN[Hex12HexNAc2]QTGYPDPSSR**	**Hex12HexNAc2**	**327**	**343**	**333**	**17.68**	**5.76**	**20.45**	**1.47**	**2.78**	**0.0493**
Biglycan	PGS1_HUMAN	[TMT0]LLQVVYLHSN[Deamid]N[dHex1Hex3HexNAc6NeuAc1]ITK	dHex1Hex3HexNAc6NeuAc1	301	314	311	12.68	9.58	14.07	6.23	1.39	0.5442
Biglycan	PGS1_HUMAN	[TMT0]LLQVVYLHSN[Deamid]N[dHex1Hex3HexNAc6NeuAc2]ITK	dHex1Hex3HexNAc6NeuAc2	301	314	311	16.00	9.80	19.98	1.77	3.98	0.7853
Biglycan	PGS1_HUMAN	[TMT0]LLQVVYLHSN[Deamid]N[dHex1Hex4HexNAc5NeuAc2]ITK	dHex1Hex4HexNAc5NeuAc2	301	314	311	16.43	8.04	17.95	4.38	1.52	0.5669
Biglycan	PGS1_HUMAN	[TMT0]LLQVVYLHSNN[dHex1Hex3HexNAc6NeuAc1]ITK	dHex1Hex3HexNAc6NeuAc1	301	314	311	12.05	9.20	13.03	6.73	0.99	0.6855
Biglycan	PGS1_HUMAN	[TMT0]LLQVVYLHSNN[dHex1Hex3HexNAc6NeuAc2]ITK	dHex1Hex3HexNAc6NeuAc2	301	314	311	16.75	8.37	19.86	1.78	3.10	1.0000
Biglycan	PGS1_HUMAN	[TMT0]LLQVVYLHSNN[dHex1Hex4HexNAc5NeuAc2]ITK	dHex1Hex4HexNAc5NeuAc2	301	314	311	15.72	7.92	16.29	5.94	0.57	0.6855
Biglycan	PGS1_HUMAN	[TMT0]LLQVVYLHSNN[Hex5HexNAc8]ITK	Hex5HexNAc8	301	314	311	18.09	6.59	19.75	2.04	1.66	0.8215
Biglycan	PGS1_HUMAN	[TMT0]LLQVVYLHSNN[Hex6HexNAc7]ITK	Hex6HexNAc7	301	314	311	17.22	6.51	16.04	6.75	-1.18	0.3025
Prosaposin	SAP_HUMAN	[TMT0]NLEK[TMT0]N[dHex1Hex2HexNAc2]STK	dHex1Hex2HexNAc2	422	429	426	18.00	5.85	15.30	7.53	-2.70	0.1598
Prosaposin	SAP_HUMAN	[TMT0]NLEK[TMT0]N[dHex1Hex2HexNAc2]STK[TMT0]	dHex1Hex2HexNAc2	422	429	426	18.34	2.48	15.77	6.72	-2.57	0.5692
Prosaposin	SAP_HUMAN	[TMT0]NLEKN[dHex1Hex2HexNAc2]STK[TMT0]	dHex1Hex2HexNAc2	422	429	426	16.65	5.79	13.62	7.68	-3.03	0.1344
Secreted frizzled-related protein 3	SFRP3_HUMAN	[TMT0]SLPWN[dHex1Hex3HexNAc6NeuAc2]MTK[TMT0]	dHex1Hex3HexNAc6NeuAc2	45	52	49	14.51	7.01	17.57	4.33	3.06	0.1413
Secreted frizzled-related protein 3	SFRP3_HUMAN	[TMT0]SLPWN[dHex1Hex4HexNAc5NeuAc2]MTK[TMT0]	dHex1Hex4HexNAc5NeuAc2	45	52	49	18.41	1.76	18.68	4.38	0.27	0.1181
Secreted frizzled-related protein 3	SFRP3_HUMAN	[TMT0]SLPWN[dHex1Hex5HexNAc4NeuAc2]MTK[TMT0]	dHex1Hex5HexNAc4NeuAc2	45	52	49	13.53	8.24	18.78	1.43	5.25	0.0545
Serotransferrin	TRFE_HUMAN	[TMT0]C[Carbamidomethyl]GLVPVLAENYN[Hex5HexNAc4NeuAc2]K	Hex5HexNAc4NeuAc2	421	433	432	22.61	0.71	23.47	0.62	0.86	0.0095
Serotransferrin	TRFE_HUMAN	[TMT0]C[Carbamidomethyl]GLVPVLAENYN[Hex5HexNAc4NeuAc2]K[TMT0]	Hex5HexNAc4NeuAc2	421	433	432	19.49	1.30	21.26	0.84	1.77	0.0038
Vitronectin	VTNC_HUMAN	[TMT0]N[Hex4HexNAc3NeuAc1]GSLFAFR	Hex4HexNAc3NeuAc1	169	176	169	17.62	5.83	17.11	4.09	-0.51	0.1413

* *P* -values were calculated using non-parametric test and with multiple testing adjustment.

ECM glycopeptides showed significant difference (*P* <0.05, Log2 fold change <-1 or >1) were highlighted using bold font.

Supplemental Table V. mRNA level of selected genes in aortic tissue from MFS patients.

Gene Name	Log2 Fold change (MFS / Control)	Unadjusted P-value *	Adjusted P-value †
ACAN	0.81	0.126	0.289
ADAMTS1	1.43	0.062	0.226
ADAMTS4	0.96	0.234	0.416
ADAMTS5	-0.01	0.533	0.652
ADAMTS7	0.07	0.396	0.558
AEBP1	1.02	0.079	0.229
BGN	1.06	0.100	0.240
CLU	0.72	0.079	0.229
COL12A1	0.76	0.079	0.229
COL4A1	0.81	0.193	0.378
COL6A3	0.28	0.777	0.806
CTGF	0.91	0.100	0.240
CTSA	-0.27	0.692	0.761
CTSG	0.17	0.955	0.955
CTSK	0.09	0.865	0.881
CTSL	-0.58	0.282	0.484
CTSS	-0.54	0.777	0.806
DCN	0.48	0.462	0.604
EFEMP1	0.41	0.396	0.558
ELN	1.06	0.100	0.240
FBLN1	0.98	0.193	0.378
FBLN5	**1.41**	**0.004**	0.099
FBN1	0.69	0.462	0.604
FMOD	0.78	0.079	0.229
GSN	1.01	0.062	0.226
HTRA1	0.73	0.027	0.136
ITGA1	**1.04**	**0.036**	0.165
ITGB1	0.97	0.015	0.117
LRP1	0.42	0.396	0.558
LTBP1	**1.31**	**0.011**	0.099
LTBP2	0.99	0.062	0.226
LTBP4	**1.62**	**0.011**	0.099
LUM	-0.31	0.396	0.558
MFAP4	**1.52**	**0.003**	0.099
MFGE8	**1.23**	**0.027**	0.136
MGP	0.45	0.234	0.416
MMP14	0.15	0.692	0.761
MMP2	0.46	0.193	0.378
MMP9	-0.42	0.692	0.761
NID1	0.70	0.336	0.527
OGN	**1.11**	**0.011**	0.099
POSTN	-0.07	0.533	0.652
PRELP	0.63	0.020	0.136
TGFB1	0.44	0.336	0.527
TGFB2	0.25	0.234	0.416
TGFB3	0.57	0.692	0.761
TGFBR1	0.10	0.777	0.806
TGFBR2	0.68	0.100	0.240
TIMP1	-0.47	0.610	0.730
TIMP2	0.84	0.027	0.136
TIMP3	0.27	0.533	0.652
TNC	-0.50	0.462	0.604
TPSB1	0.35	0.336	0.527
VCAN	0.89	0.157	0.345
ZYX	0.84	0.011	0.099

* P-values were calculated using Mann-Whitney U test.
† P-values were adjusted using the Benjamini-Hochberg method.
Proteins with unadjusted p<0.05, Log2 fold change > 1, were highlighted using bold font.

Supplemental Table VI. Proteomic analysis of aortas from non-aneurysmal BAV patients.

Protein Name	UniProt Entry Name	Average Fold change (Convex / Concave)	Standard Deviation	Unadjusted P-value *	Adjusted P-value †
Adipocyte enhancer-binding protein 1	AEBP1_HUMAN	1.16	0.54	0.779	0.955
Agrin	AGRIN_HUMAN	0.47	0.44	0.401	0.786
Annexin A2	ANXA2_HUMAN	1.08	0.24	0.779	0.955
Apolipoprotein A-I	APOA1_HUMAN	1.12	0.56	0.889	0.957
Apolipoprotein A-IV	APOA4_HUMAN	1.09	0.65	0.779	0.955
Apolipoprotein E	APOE_HUMAN	1.00	0.91	0.161	0.565
Beta-2-glycoprotein 1	APOH_HUMAN	1.22	0.82	1.000	1.000
Asporin	ASPN_HUMAN	1.52	1.37	1.000	1.000
Transforming growth factor-beta-induced protein ig-h3	BGH3_HUMAN	0.92	0.35	0.575	0.910
Cadherin-13	CAD13_HUMAN	1.54	0.83	0.161	0.565
Cathepsin D	CATD_HUMAN	1.90	0.77	0.161	0.565
Cathepsin F	CATF_HUMAN	1.83	2.00	0.889	0.957
Clusterin	CLUS_HUMAN	0.81	0.42	0.093	0.565
Chymase	CMA1_HUMAN	1.53	1.49	0.779	0.955
Collagen alpha-1(I) chain	CO1A1_HUMAN	0.92	0.32	0.263	0.660
Collagen alpha-2(I) chain	CO1A2_HUMAN	1.00	0.32	0.674	0.955
Collagen alpha-1(II) chain	CO2A1_HUMAN	1.18	0.67	0.889	0.957
Collagen alpha-1(III) chain	CO3A1_HUMAN	0.80	0.27	0.093	0.565
Collagen alpha-1(IV) chain	CO4A1_HUMAN	1.18	0.63	0.779	0.955
Collagen alpha-2(IV) chain	CO4A2_HUMAN	1.12	0.77	0.401	0.786
Collagen alpha-1(V) chain	CO5A1_HUMAN	1.59	1.19	0.208	0.616
Collagen alpha-2(V) chain	CO5A2_HUMAN	1.25	0.79	0.889	0.957
Collagen alpha-1(VI) chain	CO6A1_HUMAN	1.13	0.54	1.000	1.000
Collagen alpha-2(VI) chain	CO6A2_HUMAN	0.98	0.30	0.779	0.955
Collagen alpha-3(VI) chain	CO6A3_HUMAN	0.97	0.22	0.674	0.955
Collagen alpha-1(VIII) chain	CO8A1_HUMAN	1.27	1.17	0.889	0.957
Collagen alpha-2(VIII) chain	CO8A2_HUMAN	1.26	0.60	1.000	1.000
Collagen alpha-1(XII) chain	COCA1_HUMAN	1.03	0.50	0.674	0.955
Collagen alpha-1(XIV) chain	COEA1_HUMAN	0.96	0.67	0.401	0.786
Collagen alpha-1(XV) chain	COFA1_HUMAN	1.31	0.79	0.484	0.895
Collagen alpha-1(XVIII) chain	COIA1_HUMAN	0.91	0.25	0.123	0.565
Collagen alpha-1(XXI) chain	COLA1_HUMAN	1.77	2.99	0.123	0.565
Versican core protein	CSPG2_HUMAN	1.28	0.50	0.161	0.565
Connective tissue growth factor	CTGF_HUMAN	1.35	1.00	0.779	0.955
Collagen triple helix repeat-containing protein 1	CTHR1_HUMAN	1.89	2.05	0.779	0.955
**Dermatopontin**	**DERM_HUMAN**	**2.26**	**1.62**	**0.012**	**0.565**
Elastin	ELN_HUMAN	0.98	0.75	0.401	0.786
EMILIN-1	EMIL1_HUMAN	1.27	0.62	0.575	0.910
Fibulin-1	FBLN1_HUMAN	1.65	1.11	0.123	0.565
EGF-containing fibulin-like extracellular matrix protein 1	FBLN3_HUMAN	1.46	0.81	0.208	0.616
Fibulin-5	FBLN5_HUMAN	1.88	1.74	0.401	0.786
**Fibrillin-1**	**FBN1_HUMAN**	**1.68**	**0.97**	**0.017**	**0.565**
Fibronectin	FINC_HUMAN	1.11	0.25	0.263	0.660
Fibromodulin	FMOD_HUMAN	1.19	0.62	0.889	0.957
Gelsolin	GELS_HUMAN	1.06	0.24	0.575	0.910
Hyaluronan and proteoglycan link protein 1	HPLN1_HUMAN	1.26	1.35	0.327	0.782
Serine protease HTRA1	HTRA1_HUMAN	0.97	0.25	0.208	0.616
Insulin-like growth factor-binding protein 7	IBP7_HUMAN	0.71	0.33	0.069	0.565
Laminin subunit alpha-5	LAMA5_HUMAN	0.91	0.27	0.327	0.782
Laminin subunit beta-2	LAMB2_HUMAN	0.70	0.45	0.208	0.616
Laminin subunit gamma-1	LAMC1_HUMAN	0.83	0.36	0.093	0.565
Galectin-1	LEG1_HUMAN	0.88	0.16	0.069	0.565
Galectin-3	LEG3_HUMAN	0.81	0.70	0.161	0.565
Galectin-3-binding protein	LG3BP_HUMAN	0.92	0.37	0.263	0.660
Latent-transforming growth factor beta-binding protein 1	LTBP1_HUMAN	1.11	0.61	0.889	0.957
Latent-transforming growth factor beta-binding protein 2	LTBP2_HUMAN	1.12	0.35	0.674	0.955
Latent-transforming growth factor beta-binding protein 4	LTBP4_HUMAN	1.16	0.67	0.889	0.957
Lumican	LUM_HUMAN	1.18	0.52	0.401	0.786
Microfibrillar-associated protein 2	MFAP2_HUMAN	0.97	0.69	0.401	0.786
**Microfibril-associated glycoprotein 4**	**MFAP4_HUMAN**	**1.64**	**0.72**	**0.017**	**0.565**
Microfibrillar-associated protein 5	MFAP5_HUMAN	3.10	3.14	0.069	0.565
Lactadherin	MFGM_HUMAN	1.01	0.52	0.779	0.955
Matrix Gla protein	MGP_HUMAN	1.01	0.53	0.575	0.910
Mimecan	MIME_HUMAN	0.91	0.29	0.161	0.565
72 kDa type IV collagenase	MMP2_HUMAN	1.72	0.82	0.208	0.616
Cell surface glycoprotein MUC18	MUC18_HUMAN	1.15	0.62	0.674	0.955
Nidogen-1	NID1_HUMAN	0.91	0.10	0.043	0.565
Nidogen-2	NID2_HUMAN	0.89	0.37	0.484	0.895
Basement membrane-specific heparan sulfate proteoglycan core protein	PGBM_HUMAN	0.95	0.17	0.484	0.895
Aggrecan core protein	PGCA_HUMAN	0.97	0.63	0.401	0.786
Biglycan	PGS1_HUMAN	1.11	0.33	0.779	0.955
Decorin	PGS2_HUMAN	1.10	0.41	1.000	1.000
Periostin	POSTN_HUMAN	0.97	0.52	0.575	0.910
Prolargin	PRELP_HUMAN	1.15	0.47	0.779	0.955
Protein S100-A4	S10A4_HUMAN	2.09	1.84	0.779	0.955
Protein S100-A10	S10AA_HUMAN	1.15	0.85	0.889	0.957
Protein S100-A11	S10AB_HUMAN	1.20	0.70	0.575	0.910
Serum amyloid P-component	SAMP_HUMAN	0.89	0.56	0.263	0.660
Secreted frizzled-related protein 1	SFRP1_HUMAN	0.89	0.44	0.161	0.565
SPARC-related modular calcium-binding protein 2	SMOC2_HUMAN	2.02	1.52	0.161	0.565
Extracellular superoxide dismutase [Cu-Zn]	SODE_HUMAN	1.39	0.45	0.069	0.565
Sclerostin	SOST_HUMAN	1.80	1.48	0.263	0.660
Target of Nesh-SH3	TARSH_HUMAN	1.43	0.66	0.161	0.565
Tenascin	TENA_HUMAN	1.55	1.03	0.575	0.910
Tenascin-X	TENX_HUMAN	1.47	0.44	0.025	0.565
Tetranectin	TETN_HUMAN	1.60	1.68	0.889	0.957
Transforming growth factor beta-1	TGFB1_HUMAN	0.64	0.28	0.069	0.565
Transforming growth factor beta-1-induced transcript 1 protein	TGFI1_HUMAN	0.88	0.42	0.401	0.786
**Thrombospondin type-1 domain-containing protein 4**	**THSD4_HUMAN**	**5.47**	**9.05**	**0.036**	**0.565**
Metalloproteinase inhibitor 3	TIMP3_HUMAN	0.86	0.75	0.093	0.565
Tubulointerstitial nephritis antigen-like	TINAL_HUMAN	0.90	0.21	0.123	0.565
Serotransferrin	TRFE_HUMAN	1.31	1.05	1.000	1.000
Thrombospondin-1	TSP1_HUMAN	1.77	2.21	0.889	0.957
Vitronectin	VTNC_HUMAN	1.27	0.92	0.779	0.955

* P-values were calculated using Wilcoxon signed-rank test.
† P-values were adjusted using the Benjamini-Hockberg method.
Proteins with unadjusted p<0.05, fold change > 1.5, were highlighted using bold font.

Supplemental Table VII. Quantification of selected ECM proteins in non-aneurysm BAV and TAV samples using MRM.

Non-aneurysmal BAV patients

Protein Name	UniProt Entry	Average Fold Change Convex / Concav	Paired t-Test P-value	Peptide Sequence		Total fragment peak area (n=8)							
						P1	P2	P3	P4	P5	P6	P7	P8
Fibrillin-1	FBN1_HUMAN	1.67	0.029	sum of 3 peptides	**Concave**	4.78E+05	6.19E+05	9.64E+05	9.29E+05	7.19E+05	8.63E+05	9.29E+05	6.12E+05
					Convex	*1.44E+06*	*1.11E+06*	*1.77E+06*	*2.07E+06*	*1.21E+06*	*4.89E+05*	*7.83E+05*	*1.32E+06*
				C[+57]PTGYYLNEDTR	**Concave**	2.57E+04	3.70E+04	5.88E+04	7.02E+04	3.61E+04	4.53E+04	1.03E+05	3.61E+04
					Convex	*1.06E+05*	*8.45E+04*	*1.44E+05*	*1.49E+05*	*8.03E+04*	*4.60E+04*	*5.73E+04*	*1.36E+05*
				TC[+57]VDINEC[+57]LLEPR	**Concave**	1.59E+05	2.10E+05	3.29E+05	3.18E+05	2.67E+05	2.56E+05	3.51E+05	1.88E+05
					Convex	*5.19E+05*	*3.91E+05*	*6.05E+05*	*6.83E+05*	*4.67E+05*	*1.81E+05*	*2.07E+05*	*4.59E+05*
				YEDEEC[+57]TLPIAGR	**Concave**	2.94E+05	3.72E+05	5.75E+05	5.40E+05	4.16E+05	5.61E+05	4.75E+05	3.87E+05
					Convex	*8.20E+05*	*6.33E+05*	*1.02E+06*	*1.23E+06*	*6.63E+05*	*2.62E+05*	*5.18E+05*	*7.23E+05*
Microfibril-associated glycoprotein 4	MFAP4_HUMAN	1.54	0.025	FN[+3]GSVSFFR	**Concave**	2.04E+07	4.27E+07	2.22E+07	1.60E+07	2.94E+07	8.79E+06	1.61E+07	7.37E+06
					Convex	*3.92E+07*	*7.20E+07*	*2.44E+07*	*2.24E+07*	*3.00E+07*	*6.46E+06*	*3.21E+07*	*2.35E+07*

Non-aneurysmal TAV patients

Protein Name	UniProt Entry	Average Fold Change Convex / Concav	Paired t-Test P-value	Peptide Sequence		Total fragment peak area (n=7)						
						P1	P2	P3	P4	P5	P6	P7
Fibrillin-1	FBN1_HUMAN	2.19	0.075	sum of 3 peptides	**Concave**	3.76E+05	4.55E+05	7.43E+05	4.32E+05	5.92E+05	5.90E+05	7.51E+05
					Convex	*3.77E+05*	*6.92E+05*	*2.98E+06*	*1.46E+06*	*6.50E+05*	*6.79E+05*	*1.81E+06*
				C[+57]PTGYYLNEDTR	**Concave**	1.77E+04	2.40E+04	5.37E+04	3.25E+04	4.74E+04	3.92E+04	5.15E+04
					Convex	*1.69E+04*	*3.80E+04*	*4.21E+05*	*1.76E+05*	*4.20E+04*	*4.78E+04*	*1.18E+05*
				TC[+57]VDINEC[+57]LLEPR	**Concave**	1.71E+05	1.97E+05	2.83E+05	1.35E+05	2.29E+05	1.78E+05	3.02E+05
					Convex	*1.44E+05*	*2.33E+05*	*1.50E+06*	*4.63E+05*	*2.62E+05*	*2.32E+05*	*8.08E+05*
				YEDEEC[+57]TLPIAGR	**Concave**	1.87E+05	2.34E+05	4.07E+05	2.64E+05	3.16E+05	3.73E+05	3.98E+05
					Convex	*2.16E+05*	*4.21E+05*	*1.07E+06*	*8.16E+05*	*3.46E+05*	*3.98E+05*	*8.81E+05*
Microfibril-associated glycoprotein 4	MFAP4_HUMAN	1.35	0.231	FN[+3]GSVSFFR	**Concave**	5.19E+06	1.23E+07	3.92E+07	1.23E+06	1.95E+07	2.77E+07	9.16E+06
					Convex	*1.16E+07*	*2.11E+07*	*2.59E+07*	*3.59E+06*	*3.32E+07*	*2.70E+07*	*3.23E+07*

[+57] carbamidomethylation
[+3] O18-deglycosylation

Supplemental Table VIII. MFAP4 mRNA level in human and murine aortic SMC after TGF-β1 and ALKi treatment.

Gene Name	TGF-β1			TGF-β1 + ALKi			
	Fold change vs Control	Standard deviation	P-value vs Control	Fold change vs Control	Standard deviation	P-value vs Control	P-value vs TGF-β1
**Human *MFAP4***	1.45	0.50	0.015	0.77	0.19	0.122	0.036
**Mouse *Mfap4***	13.06	3.71	<0.001	0.25	0.02	<0.001	<0.001

P-values were calculated using Student's t-test

Supplemental Table IX. mRNA level of selected genes in human aortic SMCs after si-MFAP4 treatment.

Gene Name	Log2 Fold change (si-MFAP4 / Control)	Standard deviation of Log2 Fold Change	Unadjusted P-value *	Adjusted P-value †
ACAN	-0.139	0.790	0.470	0.495
	-0.264	*1.152*	-	-
ADAMTS1	-0.799	0.536	0.001	0.004
	-1.257	*0.060*	*0.031*	*0.038*
ADAMTS4	0.541	0.817	0.041	0.055
	0.072	*0.121*	*0.313*	*0.313*
ADAMTS5	-1.569	0.523	0.001	0.004
EFEMP1	-0.401	0.443	0.003	0.006
ELN	2.348	1.311	0.001	0.004
	1.038	*0.343*	*0.031*	*0.038*
FBLN5	0.531	0.500	0.004	0.006
FBN1	-0.493	0.613	0.002	0.005
	-0.699	*0.082*	*0.031*	*0.038*
LTBP2	0.857	0.871	0.001	0.004
LTBP4	0.615	0.638	0.002	0.005
MFAP4	-4.339	0.964	0.001	0.004
	-5.446	*0.224*	*0.031*	*0.038*
MFGE8	0.677	0.495	0.001	0.004
MMP2	0.310	0.600	0.056	0.065
MMP3	-0.302	0.506	0.048	0.060
MMP12	-0.270	1.149	0.730	0.730
TGFB1	0.335	0.426	0.016	0.022
TGFB2	0.161	0.621	0.331	0.367
TGFB3	0.672	0.624	0.005	0.008
TGFBR1	1.488	0.562	0.001	0.004
VCAN	-0.502	0.396	0.004	0.007
	-1.505	*0.132*	*0.031*	*0.038*

* P-values were calculated using Wilcoxon signed-rank test.
† P-values were adjusted using the Benjamini-Hockberg method.
Results from the second si-MFAP4 were shown in *Italic fonts*

Supplemental Table X: Patient demographics of the MFS patients included in the plasma MFAP4 analysis.

	Lower tertile	Middle tertile	Upper tertile	P-value
n	32	32	32	
Plasma MFAP4 concentration (ng/ml)	54.65 (40.8-60.6)	65.20 (60.6-69.9) †	79.05 (69.9-123.8) †‡	<0.0001
Age (years)*	36.0 (17-61)	30.5 (18-62)	38.0 (20-62)	0.23
Gender				
Male	50%	53%	69%	0.26
Female	50%	47%	31%	
Blood pressure lowering agent*				
β-blockers	69%	78%	72%	0.69
Calcium channel blocker	3%	0%	6%	0.36
ACE inhibitor	0%	0%	3%	0.36
Losartan during follow-up	56%	56%	47%	0.68
Prophylactic aortic surgery before follow-up	25%	28%	34%	0.70
Prophylactic aortic surgery during follow-up	28%	16%	19%	0.44
Dissection during follow-up				
type A	0%	0%	0%	--
type B	0%	0%	16%	<0.01
Aortic root diameter (mm)* (n=68)	45 (35-49)	42 (32-49)	46 (38-53) ‡	0.04
Aortic root diameter cut-off $\leq$ 50 mm* (n=68)	0%	0%	19%	0.02
Aortic growth rate (mm/yr) (n=64)	0.62 (0.00-1.37)	0.59 (0.00-1.33)	0.55 (0.00-1.33)	0.97
Distensibility (mmHg (-1)*10^{-3}) at Level 1* (n=79)	2.90 (0.79-7.02)	3.34 (1.30-6.52)	2.60 (0.93-5.45)	0.18
Distensibility (mmHg (-1)*10^{-3}) at Level 2* (n=78)	3.00 (1.28-8.55)	3.35 (1.75-6.36)	2.49 (0.92-5.92) ‡	0.04
Distensibility (mmHg (-1)*10^{-3}) at Level 3* (n=78)	6.65 (4.15-14.35)	6.33 (3.54-15.50)	4.39 (2.06-11.90) †‡	0.01
Distensibility (mmHg (-1)*10^{-3}) at Level 4* (n=70)	5.17 (2.73-34.38)	6.36 (2.18-12.50)	5.82 (2.14-18.31)	0.88

The MFS patients included in the plasma MFAP4 analysis (n=96) were subdivided into tertiles. The patient demographics of the tertiles are summarized. Continuous data are presented as median with range (Kruskal Wallis test) and categorical data as percentages (chi square test where appropriate). When statistical significance was reached with the aforementioned analysis, a subanalysis with a Mann Whitney U or chi square/ Fisher exact test was performed to analyze differences between two groups.

*= at time of inclusion

†= significantly different from tertile 1

‡= significantly different from tertile 2

ACE = Angiotensin-converting enzyme

Supplemental Table XI. Transition list of MRM method used for ECM proteins quantification.

UniProt Entry	Peptide Sequence	Parent ion m/z	Parent ion charge	Collision energy (V)	Product Ion m/z			
FBN1_HUMAN	C[+57]PTGYYLNEDTR	744.8248	2	27.8	y2: 276.1666	y5: 634.2791	y7: 910.4265	y9: 1130.5113
FBN1_HUMAN	TC[+57]VDINEC[+57]LLEPR	809.8818	2	30.1	y6: 787.4131	y8: 1030.4986	y10: 1258.6096	y11: 1357.6780
FBN1_HUMAN	YEDEEC[+57]TLPIAGR	776.8510	2	28.9	y5: 513.3144	y8: 887.4767	y9: 1016.5193	y11: 1260.5889
MFAP4_HUMAN	FN[+3]GSVSFFR	532.2583	2	20.1	y4: 556.2878	y5: 655.3562	y7: 799.4097	y8: 916.4409

[+57] carbamidomethylation
[+3] O18-deamidation
